# Conditional knockdown of hepatic PCSK9 ameliorates high-fat diet-induced liver inflammation in mice

**DOI:** 10.3389/fphar.2025.1528250

**Published:** 2025-02-03

**Authors:** Xue-Ying Zhang, Qing-Qing Lu, Yan-Jie Li, Shan-Rui Shi, Chao-Nan Ma, Miao Miao, Shou-Dong Guo

**Affiliations:** Institute of Lipid Metabolism and Atherosclerosis, School of Pharmacy, Shandong Second Medical University, Weifang, China

**Keywords:** inflammation, MAPK, PCSK9 inhibitor, PCSK9 siRNA, Toll-like receptor

## Abstract

**Instruction:**

Accumulating evidence has shown that proprotein convertase subtilisin/kexin type 9 (PCSK9) is associated with inflammation in the vascular system. However, the roles of PCSK9 in hepatic inflammation remain unclear. Because PCSK9 is mainly expressed in the liver and modulates lipid uptake through low-density lipoprotein receptor family members, the present study aimed to elucidate the effect of conditional knockdown of hepatic PCSK9 on hyperlipidemia-induced inflammation and the underlying mechanisms of action.

**Methods:**

PCSK9flox/flox mice were bred with ALB-Cre^+^ mice to obtain hepatic PCSK9^*(−/−)*^, PCSK9^*(+/−)*^, and PCSK9^*(+/+)*^ mice. These mice were fed with a high-fat diet for 9 weeks to induce inflammation. The effects of conditional knockdown of hepatic PCSK9 on inflammation and the underlying mechanisms were investigated by molecular biological techniques. Moreover, the findings were verified in vitro using HepG2 cells.

**Results and Discussion:**

Conditional knockdown of hepatic PCSK9 remarkably decreased plasma levels of total cholesterol and alleviated hyperlipidemia-induced liver injury. Mechanistically, conditional knockdown of hepatic PCSK9 significantly reduced the levels of pro-inflammatory factors by downregulating the expression of Toll-like receptors, mitogen-activated protein kinase (MAPK), and phosphoinositide-3 kinase/protein kinase B, which subsequently attenuated the expression of downstream molecules, namely nuclear factor kappa-B and activator protein-1. The related mechanisms were confirmed using lipid-loaded HepG2 cells together with PCSK9 siRNA, alirocumab (anti-PCSK9 antibody), and/or a p38-MAPK inhibitor. These findings confirmed that conditional knockdown of hepatic PCSK9 attenuates liver inflammation following hyperlipidemia induction by modulating multiple signaling pathways; this suggests that targeting PCSK9 knockdown/inhibition with appropriate agents is useful not only for treating hyperlipidemia but also for ameliorating hyperlipidemia-induced liver inflammation.

## Introduction

Loss-of-function mutations in the hepatic protein proprotein convertase subtilisin/kexin type 9 (PCSK9) reduce plasma low-density lipoprotein (LDL) cholesterol (LDL-c) levels, and vice verse (Meng et al., 2023). Mechanistically, PCSK9 interacts with LDL receptor (LDLR) and other LDLR family members to induce their degradation, leading to development of hyperlipidemia. Compared to statins, PCSK9 inhibitors, such as monoclonal antibodies evolocumab and alirocumab, exhibit more robust effects in reducing LDL-c levels (Zhang et al., 2007; Dixon et al., 2016; Coppinger et al., 2022; Momtazi-Borojeni et al., 2022). Therefore, together with statin therapy, anti-PCSK9 antibodies are currently used for the clinical treatment of hyperlipidemia to decrease the risk of residual cardiovascular diseases (CVDs), a leading cause of death in humans. With advancement in research on PCSK9, this hepatic protein was shown to possess multifaceted functions; furthermore, PCSK9 inhibitors were found to exhibit LDL-c-independent effects (Ferri and Ruscica, 2016; Ugovšek and Šebeštjen, 2022). Moreover, given that atherosclerosis, the basic pathological change occurring in CVD, is primarily promoted by chronic inflammation induced by hyperlipidemia (Zhao et al., 2021), the role of PCSK9 in inflammation has attracted the attention of many researchers in recent years.

In African Americans with CVD, the fasting levels of inflammatory cytokine interleukin (IL)-8 is positively associated with PCSK9 (Baginski et al., 2022). In a rat model, hepatic PCSK9 synthesis level and circulating PCSK9 level are related to plasma IL-13 levels (Low et al., 2020). Moreover, plasma PCSK9 levels show a positive association with the levels of proinflammatory factors, including IL-6, IL-1β, and tumor necrosis factor (TNF)-α (Ding et al., 2020a; Punch et al., 2022). Based on these observations, PCSK9 and inflammation appear to have a mutual promoting effect. On the one hand, inflammatory stimuli increase PCSK9 secretion in macrophages and some tissues, including aorta and heart (Andreadou et al., 2020). For example, TNF-α and interferon γ synergistically promote PCSK9 expression by upregulating sterol regulatory element binding protein 2 *in vitro* (Zhang et al., 2023). In addition to proinflammatory cytokines, high-fat diet (HFD)-induced hyperlipidemia also enhances PCSK9 expression potentially by modulating Toll-like receptor (TLR) 4 and its downstream signaling factors myeloid differentiation primary response gene 88 (MyD88) and nuclear factor kappa-B (NF-κB) (Liu et al., 2020). On the other hand, PCSK9 can also induce inflammation by elevating lipid accumulation in macrophages and enhancing the TLR/NF-κB signaling pathway (Andreadou et al., 2020); this process is mediated by LDLR-related protein 5 (Badimon et al., 2021). Consistent with this observation, adeno-associated virus-mediated overexpression of PCSK9 promotes atherosclerosis development and increases the numbers of proinflammatory monocytes/macrophages in C57BL/6J mice (Peled et al., 2017; Keeter et al., 2022). Furthermore, lentivirus carrying PCSK9 small hairpin RNA attenuates aortic inflammation by suppressing the TLR-4/NF-κB signaling pathway (Tang et al., 2017; Yurtseven et al., 2020). These findings indicate that PCSK9 is closely associated with vascular inflammation.

HFD-induced hyperlipidemia is a known high-risk factor for the development of nonalcoholic steatohepatitis and nonalcoholic fatty liver disease; the former has been recently renamed as metabolic dysfunction-associated steatohepatitis (MASH) and the latter has been recently renamed as metabolic dysfunction-associated fatty liver disease (MAFLD) (Lebeau et al., 2019; Wu et al., 2023; Fang et al., 2024). Although PCSK9 shows a positive relationship with body mass index and is considered to be associated with metabolic syndrome (Ferri and Ruscica, 2016), the effects of PCSK9 on MASH and MAFLD are contradictory (Momtazi-Borojeni et al., 2022). Importantly, the role of PCSK9 in hepatic inflammation and the underlying mechanisms of action have been seldomly reported because previous studies mainly focused on hepatic PCSK9-mediated lipid metabolism. Notably, PCSK9-deificency was shown to increase liver weight, hepatic TG accumulation, and inflammation in mice by upregulating CD36-mediated fatty acid internalization and TG storage (Demers et al., 2015). This was particularly observed in PCSK9-deficent mice fed an HFD (Lebeau et al., 2019), thus suggesting that the complete deletion of PCSK9 induces MAFLD and MASH (Lebeau et al., 2022). However, because PCSK9 deficiency is an extreme condition, the related studies with PCSK9-deficent models may have inaccurately predicted the role of PCSK9 in hyperlipidemia-induced inflammation. Because the secretory PCSK9 protein is also produced by the intestine and other tissues (Zaid et al., 2008), we hypothesized that conditional knockdown of hepatic PCSK9 could attenuate hyperlipidemia-induced liver inflammation as PCSK9 secreted by extrahepatic tissues is transported to the liver, thereby partially compensating PCSK9 deficiency. The present study aimed to validate this hypothesis by using PCSK9^liver*(−/−)*^, PCSK9^liver*(+/−)*^, and PCSK9^liver*(+/+)*^ mice.

## Materials and methods

### Materials

Isoflurane (R510-22-10) was bought from RWD Life science Co., Ltd. (Shenzhen, China). Adezmapimod (SB203580), a potent p38-MAPK inhibitor, was purchased from Selleck chemicals, LLC, United States. An anti-PCSK9 monoclonal antibody, alirocumab, was obtained from Sanofi S.A. Penicillin-streptomycin liquid (P1400), 0.25% trypsin-EDTA solution (T1300), palmitic acid (N-16-A), and cis-9-octadecenoic acid (IC1350) were purchased from Solarbio Life Sciences (Beijing, China). Human hepatocellular carcinoma cell line HepG2 (SCSP-150) was bought from the Cell Bank of Chinese Academy of Sciences (Shanghai, China). Lipofectamine 3,000 reagent (L3000008) was purchased from Invitrogen Corporation, United States. Dulbecco′s modified Eagle′s medium (DMEM) (C11995500BT) and Opti-MEM I reduced serum medium (31,985,062) were the products of Gibco ThermoFisher Scientific Corporation (CA, United States). Fetal bovine serum (FBS) (BS1612-105) was obtained from Bioexplorer Life Sciences (C.A., China). High-fat diet (License No.: SCXK 2020–0004) was the product of Keao Xieli Feed Co., Ltd. (Tianjin, China). Heparinized capillary tubes (2501) were purchased from Kimble Chase Life Science and Research Products LLC. (NJ, United States). NEG-50™ frozen section medium (6502) was bought from Richard-Allan Scientific LLC. (Kalamazoo, MI, United States). The assay kits for total cholesterol (TC, 100,000,180) and TG (100,000,220) were provided by Biosino Bio-technology and Science Inc. (Beijing, China). The antibody information was provided in Supplementary Table S1. The rest reagents used in this study were of analytical grade.

### Mouse breeding

Animal experiments were approved by the Laboratory Animal Ethical Committee of Shandong Second Medical University and carried out according to NIH guidelines for the Care and Use of Animals (2020SDL106). In this study, PCSK9^*flox/flox*^ mice and albumin (Alb)-Cre mice were purchased from GemPharmatech LLC. (Jiangsu, China). The mice were kept in a temperature-controlled room (23°C) with controlled light/dark cycles (12 h/12 h). PCSK9^*flox/flox*^ mice were bred with Alb-Cre mice to produce Alb-Cre^-^/PCSK9^*flox/flox*^ (PCSK9^liver*(+/+)*^ or wild type) mice, Alb-Cre^*+*^/PCSK9^*flox/flox*^ (hepatic-specific PCSK9 knockout, PCSK9^liver*(−/−)*^) mice; and Alb-Cre^*+*^/PCSK9^*flox/WT*^ (PCSK9^liver*(+/−)*^) mice. The primers for genotyping are listed in Table 1.

**TABLE 1:** The primers for identification of ALB and PCSK9.

Primers	Sequence (5ʹ-3ʹ)	Molecular weight
ALB primer 1-Forward 1	GGG​CAG​TCT​GGT​ACT​TCC​AAG​CT	Target: 340 bp
ALB primer 1-Reverse 1	TAG​CTA​CCT​ATG​CGA​TCC​AAA​CAA​C	Wild type: None
ALB primer 2- Forward 2	CCT​GCT​GTC​CAT​TCC​TTA​TTC​CAT	Target: 356 bp
ALB primer 2-Reverse 2	ATA​TCC​CCT​TGT​TCC​CTT​TCT​GC	Wild type: None
ALB primer 3-Forward 3	CAG​CAA​AAC​CTG​GCT​GTG​GAT​C	Target: None
ALB primer 3-Reverse 3	ATG​AGC​CAC​CAT​GTG​GGT​GTC	Wild type: 412 bp
PCSK9 primer 1- Forward 1	TGG​CAG​TCA​GGA​GCA​GTA​TGT​CCA​T	Target: 378 bp
PCSK9 primer 1-Reverse 1	GAA​TCT​TGA​GTT​GCA​CCA​ATC​ATT​GG	Wild type: 278 bp
PCSK9 primer 2- Forward 2	CTA​AAC​CCG​ACT​GAC​CCA​CTA​TTT​G	Target: 346 bp
PCSK9 primer 2-Reverse 2	CAG​TCC​TAC​CAG​ATC​CAG​ACC​ACC​TT	Wild type: 245bp

### Animal grouping and intervention with a high-fat diet

Approximately 10-week-old male PCSK9^liver*(+/+)*^ (n = 10), PCSK9^liver*(−/−)*^ (n = 10), and PCSK9^liver*(+/−)*^ (n = 6) mice were fed an HFD containing 21% fat and 0.15% cholesterol for 9 weeks. On the last day of HFD feeding, mice were fasted for 6 h, weighted, and anaesthetized using isoflurane before sampling. Briefly, mouse blood was collected from the retro-orbital venous plexus by using heparinized capillary tubes, and plasma was obtained by centrifugation of the blood samples at 1,100 × *g* for 30 min. Next, mouse heart was exposed, and the auricle was cut using surgical scissors. The mouse was perfused with phosphate buffer saline (PBS, pH = 7.4) through the left ventricle to remove the residual blood before sampling. For comparative analyses, 6 age-matched PCSK9^*flox/flox*^ mice fed a chow diet were randomly selected for plasma levels of TC and TG determination and Western blotting experiments.

### Plasma and liver analysis

Plasma TC and TG levels were determined using appropriate assay kits in accordance with the manufacturers’ instructions. To determine TC and TG distributions in very low-density lipoprotein (VLDL), LDL, and high-density lipoprotein (HDL) particles, mixed plasma (100 μL) from each group was analyzed using a Superose™ 6 10/300 gel chromatography column linked to an ÄKTA Fast Protein Liquid Chromatography (FPLC) system. The column was eluted with 0.9% sodium chloride solution, and the eluate was collected using an automatic collector with 0.5 mL in each fraction. Hepatic lipids were extracted as reported previously with minor modifications (Tran et al., 2020; Li et al., 2022). Briefly, approximately 100 mg liver samples were homogenized in 1 mL PBS, and 4 mL chloroform and methanol mixture (2:1, v/v) was added and shaken well. This suspension was centrifuged at 1800 × *g* for 10 min to obtain the bottom chloroform phase containing lipid, which was subjected to blow-drying with nitrogen. The obtained lipids were resolved in 100 μL ethanol and used for determining TC and TG levels by using commercially available assay kits as described above.

### Haematoxylin and eosin (H & E) staining and Oil Red O staining

Liver tissues were fixed with freshly prepared 4% paraformaldehyde for 24 h, embedded in paraffin by using a JB-P5 embedding machine (Wuhan, China), and cut into 7-µm-thick sections by using a RM2016 pathology slicer (Shanghai, China). Next, the sections were dewaxed in xylene, passed through a gradient of ethanol concentrations, and rinsed with tap water. Subsequently, the sections were stained with hematoxylin solution for 4 min, rinsed with tap water, and stained with eosin for 15 s. Finally, the sections were dehydrated and sealed with neutral gum (Zhang et al., 2022). For Oil Red O staining, fresh liver tissues were dissected, dried with a filter paper, trimmed into a cube shape (side length: 5 mm), and embedded in NEG-50™ frozen section medium. Next, the tissues were fixed on the slicer, and 8-µm-thick tissue sections were obtained using a Cryostar NX50 machine (Thermo, United States). The sections were subsequently stained with freshly prepared Oil Red O dye solution (Yin et al., 2019). The stained images were recorded on an Axiocam 506 color camera (Zeiss, Jena, Germany), and the staining areas were analyzed by using ImageJ software.

### Lipid accumulation induced inflammation in HepG2 cells

HepG2 cells were seeded in 6-well plates at a density of 2.0 × 10^5^/well. The cells were cultured in a humidified 5% CO_2_ at 37°C. These cells were randomly divided into blank, model, alirocumab (Pm, 10 μg/mL), SB203580 (p38i, 30 µM), and combination (Pm + i, alirocumab 10 μg/mL and SB203580 30 µM) groups. Except for the blank group, the rest groups were treated with 0.5 mM cis-9-octadecenoic acid and 0.25 mM palmitic acid for 24 h to establish a lipid-loaded cell model (Li et al., 2020; Zulkapli et al., 2023; Zhang et al., 2024). Next, the cells were treated with the corresponding drugs for another 24 h prior to sampling.

For the siRNA transfection experiments, the HepG2 cells were initially incubated with 0.5 mM cis-9-octadecenoic acid and 0.25 mM palmitic acid for 24 h to establish a lipid-loaded cell model. Next, the cells were randomly divided into negative control (NC), PCSK9 siRNA1 (siR-1), and PCSK9 siRNA2 (siR-2) groups. The siRNAs against PCSK9 were synthesized by Guangzhou RiboBio Co., Ltd. (Guangzhou, China) and the sequences were listed in Table 2 (Tang et al., 2012; Xiao et al., 2019). Briefly, the cells were transfected with the corresponding siRNA by using Lipofectamine 3,000 as reported previously (Tang et al., 2012; Deng et al., 2020). The cells were cultivated for another 24 h prior to sampling. In this study, the normal HepG2 cells were used as blank control. Moreover, the expression levels of several inflammation-related proteins in the blank control, model, and NC groups were investigated to confirm lipid accumulation-induced inflammation and to rule out the potential effects caused by the control plasmid.

**TABLE 2:** The primers used for polymerase chain reaction (PCR).

Name	Primers	Sequence (5ʹ-3ʹ)
*h-PCSK9* *siRNA1*	Forward	GGCAGAGACUGAUCCACUUdTdT
	Reverse	dTdTCCGUCUCUGACUAGGUGAA
*h-PCSK9* *siRNA2*	Forward	GGG​UCA​UGG​UCA​CCG​ACU​Utt
	Reverse	AAG​UCG​GUG​ACC​AUG​ACC​Ctg
*m-GAPDH*	Forward	AAG​AAG​GTG​GTG​AAG​CAG​GCA​TC
	Reverse	CGG​CAT​CGA​AGG​TGG​AAG​AGT​G
*m-PCSK9*	Forward	AGGCACAGGCTGA TCCACTTCT
	Reverse	AGC​AGC​CCA​ACA​ACT​CCT​CAT​C
*m-LDLR*	Forward	GCA​GCC​ACA​TGG​TAT​GAG​GTT​CC
	Reverse	TGA​TGT​TCT​TCA​GCC​GCC​AGT​TC
*m-P65*	Forward	CTC​CTG​TTC​ATC​CGA​CTC​CC
	Reverse	AGT​CAG​TGC​CTG​TTT​TAC​GTT
*m-P38*	Forward	GGC​AGG​AGC​TGA​ACA​AGA​CCA​TC
	Reverse	AGC​AGA​CGC​AAC​TCT​CGG​TAG​G
*m-ERK1/2*	Forward	GCC​TTC​CAA​CCT​CCT​GCT​GAA​C
	Reverse	CGT​ACT​CTG​TCA​AGA​ACC​CTG​TGT​G
*m-JNK*	Forward	CGC​CTT​ATG​TGG​TGA​CTC​GCT​AC
	Reverse	CTC​CCA​TGA​TGC​ACC​CAA​CTG​AC
*m-TLR2*	Forward	AAG​ATG​CGC​TTC​CTG​AAT​TTG
	Reverse	TCC​AGC​GTC​TGA​GGA​ATG​C
*m-TLR4*	Forward	ATG​GCA​TGG​CTT​ACA​CCA​CC
	Reverse	GAG​GCC​AAT​TTT​GTC​TCC​ACA
*m-PI3K*	Forward	TGCAGCACAATGACTCCC
	Reverse	TTC​ATC​GCC​TCT​GTT​GTG​CAT
*m-AKT*	Forward	TTC​ACA​ACC​AGG​ACC​AGC​AGA
	Reverse	ATC​CAT​GAG​GAT​CAG​CTC​GAA​C
*m-mTOR*	Forward	ACC​GTC​CGC​CTT​CAC​AGA​TAC
	Reverse	CGT​TCC​TTC​TCC​TTC​TTG​ACA​CAG
*m-IL-6*	Forward	CTT​CTT​GGG​ACT​GAT​GCT​GGT​GAC
	Reverse	TCT​GTT​GGG​AGT​GGT​ATC​CTC​TGT​G
*m-IL-1β*	Forward	CCA​GGA​TGA​GGA​CAT​GAG​CA
	Reverse	CGG​AGC​CTG​TAG​TGC​AGT​TG
*m-TNFα*	Forward	ACT​CCA​GGC​GGT​GCC​TAT​GT
	Reverse	AGT​GTG​AGG​GTC​TGG​GCC​AT

### Immunoblotting

Approximately 50 mg liver tissues were homogenized in 500 µL RIPA lysis buffer and allowed to digest on ice for 30 min. The protein concentration was adjusted to approximately 3.0 g/mL according to the results of the bicinchoninic acid assay. The cell sample in each well was treated with 60 µL RIPA lysis buffer to obtain total proteins. This experiment was conducted by following previously reported methods (Li et al., 2020; Fu et al., 2021; Zhang et al., 2022; Zulkapli et al., 2023; Zhang et al., 2024). In brief, equal amounts of proteins were separated by sodium dodecyl sulfate-polyacrylamide gel electrophoresis on gels with different concentrations (6%–12%). The separated proteins were transferred onto 0.45 μm polyvinylidene fluoride (PVDF) membranes (or 0.22 μm PVDF membranes for proteins with a molecular weight of <30 kDa) by electroblotting. These PVDF membranes were blocked with 5% nonfat dry milk dissolved in Tris-buffered saline (TBS) at room temperature for 2–4 h. Next, these PVDF membranes were incubated with the corresponding primary antibodies at 4°C for 12 h. The PVDF membranes were then washed with TBS containing 0.1% Tween for 3–5 times and incubated with the corresponding secondary antibodies for 2 h at room temperature. Finally, immunoblots were visualized by enhanced chemiluminescence reaction, and the images were captured with an SH-Compact 523 ECL imaging system, a product of Shenhua Science Technology Co., Ltd. (Hangzhou, China). Densitometry analysis was conducted using ImageJ software. The protein levels were normalized by housekeeping protein glyceraldehyde-3-phosphate dehydrogenase (GAPDH) or β-actin.

### Quantitative reverse transcription-polymerase chain reaction (RT-qPCR)

An appropriate amount of liver tissue (∼50 mg) was ground to powder under liquid nitrogen protection in a RNase free mortar. The obtained powder was extracted with Trizol (Spark Jade, Qingdao, China) in accordance with the manufacturer’s instructions. The concentration and purity of total RNA were determined by estimating the absorbance at 260 nm and 280 nm with a NanoDrop™ OneC UV spectrophotometer (Thermo Fisher, United States). cDNA was subsequently synthesized in an ABI Veriti™ 96-well thermal cycler (MA, United States) in accordance with the manufacturer’s instructions. RT-qPCR was performed in an ABI QuantStudio3 PCR system (Waltham, MA, United States) as reported previously (Yang et al., 2019; Li et al., 2020; Li et al., 2022). Gene-specific primers (Table 2) were designed and synthesized by Sangon Biotechnology Co., Ltd. (Shanghai, China). The relative expression of target genes was adjusted based on the *C*_*t*_ number of GAPDH and calculated according to the 2^−ΔΔCt^ method.

### Data analysis

Statistical analysis was performed using one-way analysis of variance (ANOVA). The results were presented as the mean ± standard deviation (SD) for at least three independent experiments. Calculations were performed using GraphPad Prism sofeware, version 7.0 (San Diego, CA, United States). Differences were considered to be significant at a *P* < 0.05.

## Results

### Generation of hepatic PCSK9 conditional knockdown mice

After several rounds of breeding, we obtained colonies of hepatic PCSK9^liver*(+/+)*^, PCSK9^liver*(+/−)*^, and PCSK9^liver*(−/−)*^ mice based on the genotyping results (Supplementary Figure S1). We used three and two pairs of primers to determine the gene phenotype of ALB-Cre^+^ and PCSK9^*flox/flox*^, respectively. Table 1 shows the primers and target molecular weight for identifying PCSK9^liver*(+/+)*^, PCSK9^liver*(+/−)*^, and PCSK9^liver*(−/−)*^ mice. Taking the mice numbered as 1–9 for example: mice number 3, 4, and 6–9 were determined as PCSK9^liver*(−/−)*^ mice, while mice number 1, 2, and 5 were determined as PCSK9^liver*(+/−)*^ mice according to the molecular weight of the observed bands following agarose gel electrophoresis (Supplementary Figures S1A–C). In summary, hepatic PCSK9 conditional knockdown mice were successfully bred in our laboratory. Next, we investigated whether hepatic PCSK9 knockdown ameliorates hyperlipidemia-induced inflammation. The *in vivo* experimental design was shown in Supplementary Figure S1D.

### Effects of conditional knockdown of hepatic PCSK9 on PCSK9 and LDLR expression levels, lipid profile, and liver injury following HFD challenge

**FIGURE 1:**
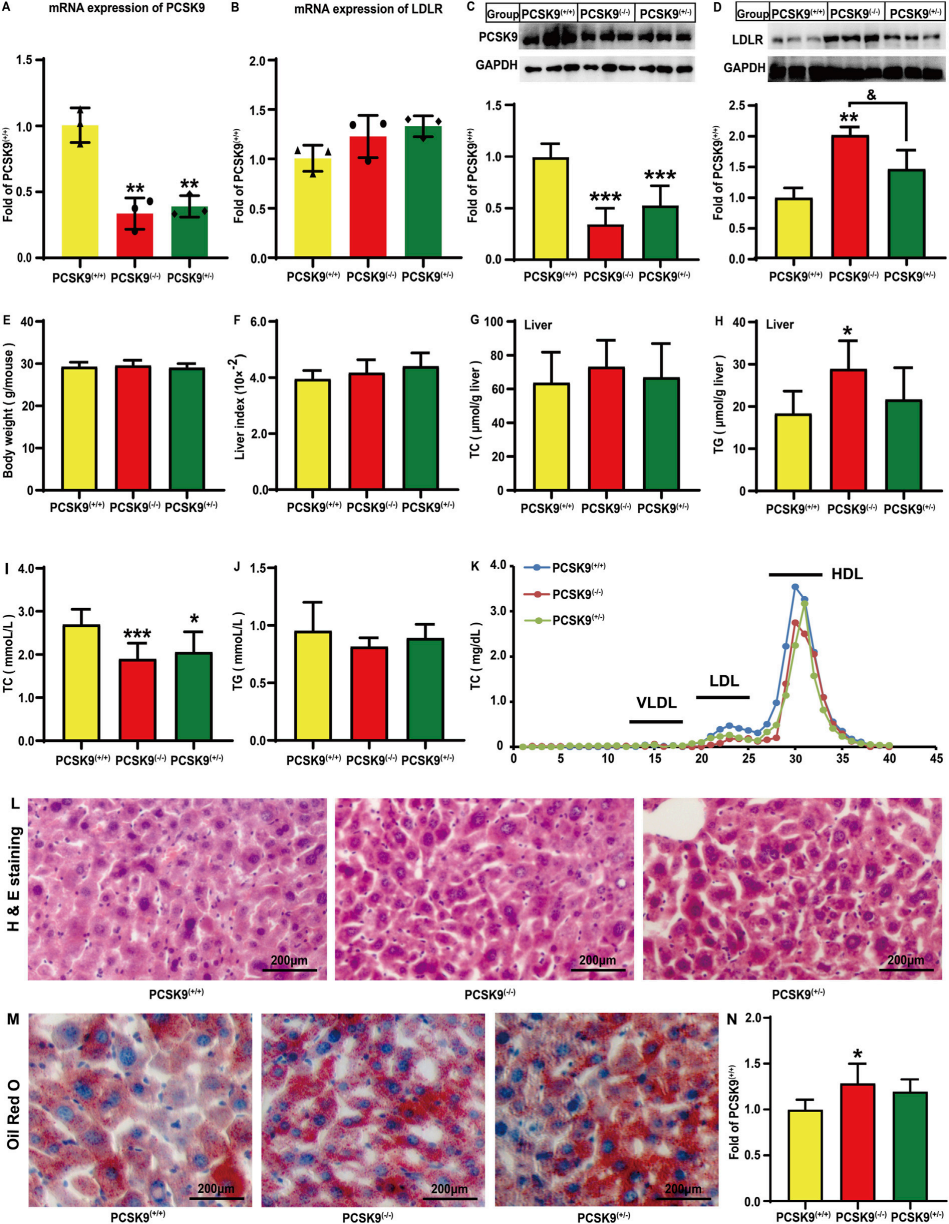
Effects of conditional knockdown of hepatic PCSK9 on the expression levels of PCSK9 and LDLR, plasma and hepatic lipid profiles, body weight, liver indices, H & E staining, and Oil Red O staining. Expression of **(A)**, PCSK9 (n = 3); **(B)**, LDLR (n = 3). Levels of **(C)**, PCSK9 (n = 6); **(D)**, LDLR (n = 6) proteins. **(E)**, Body weights and **(F)**, liver indices. Hepatic levels of **(G)**, TC; and **(H)**, TG (n = 6). Plasma levels of **(I)**, TC; and **(J)**, TG. **(K)**, Plasma TC profiles in different lipoprotein fractions after ÄKTA-FPLC separation; **(L)**, Typical histological images obtained by H & E staining. **(M)**, Typical histological images obtained by oil red O staining. **(N)**, Statistical analysis of Oil Red O staining areas (n = 5). PCSK9^*(+/+)*^: PCSK9^liver*(+/+)*^ mice; PCSK9^*(−/−)*^: PCSK9^liver*(−/−)*^ mice; PCSK9^*(+/−)*^: PCSK9^liver*(+/−)*^ mice. For **(E, F, I, J)**, PCSK9^liver*(+/+)*^ (n = 10), PCSK9^liver*(−/−)*^ (n = 10), PCSK9^liver*(+/−)*^ (n = 6). These abbreviations are suitable for the rest figures. ^*^means *p* < 0.05 vs*.* PCSK9^liver*(+/+)*^ group; ^**^means *p* < 0.01 vs*.* PCSK9^liver*(+/+)*^ group; ^***^means *p* < 0.001 vs*.* PCSK9^liver*(+/+)*^ group; ^&^means *p* < 0.05 vs*.* PCSK9^liver*(+/−)*^ group.

First of all, we demonstrated that HFD successfully induced hyperlipidemia in mice compared to those fed a chow diet (Figures 1I, J, Supplementary Figures S2A, B). Importantly, the levels of PCSK9, TNF-α, IL-1β, TLR-2, and TLR-4, and the levels of phosphorylated NF-κB, AP-1, MyD88, phosphoinositide 3-kinase (PI3K), protein kinase B (PKB/AKT), mTOR, p38-MAPK, and ERK1/2 proteins were significantly upregulated in the liver of PCSK9^liver*(+/+)*^ mice fed a high-fat diet, as compared to the mice fed a chow diet (Supplementary Figure S2 and Supplementary Material S1). These results revealed the hyperlipidemia induces inflammation in liver by affecting multiple signaling pathways. In the following sections, we aimed to determine the effects of conditional knockdown of hepatic PCSK9 and the underlying mechanisms of action on modulating hyperlipidemia-induced chronic liver inflammation.

The gene and protein expression levels of hepatic PCSK9 and LDLR were determined in mice fed an HFD. As expected, compared to PCSK9^liver*(+/+)*^ mice, PCSK9^liver*(−/−)*^ and PCSK9^liver*(+/−)*^ mice exhibited a decrease in the mRNA expression level of *PCSK9* by 66.4% and 60.9%, respectively (*P* < 0.01, Figure 1A). Conditional knockdown of hepatic PCSK9 did not affect the *LDLR* gene expression (Figure 1B). Consistent with this observation, PCSK9^liver*(−/−)*^ and PCSK9^liver*(+/−)*^ mice showed a significant decrease in the hepatic PCSK9 levels by approximately 65.5% and 47.0%, respectively, as compared to PCSK9^liver*(+/+)*^ mice (*P* < 0.05, Figure 1C). The hepatic LDLR levels were significantly increased by approximately 102% (*P* < 0.01) and 46.7% in PCSK9^liver*(−/−)*^ and PCSK9^liver*(+/−)*^ mice, respectively, as compared to those in PCSK9^liver*(+/+)*^ mice (Figure 1D). Notably, PCSK9^liver*(−/−)*^ mice exhibited significantly higher LDLR level than PCSK9^liver*(+/−)*^ mice. As shown in Figures 1E, F, mice body weight and liver indices showed no significant changes (Supplementary Material S2). We also did not find alterations in hepatic TC levels among the mice groups (Figure 1G); however, the hepatic TG levels were increased by 57.8% in PCSK9^liver*(−/−)*^ mice as compared to that in PCSK9^liver*(+/+)*^ mice (Figure 1H). A noteworthy finding is PCSK9^liver*(−/−)*^ and PCSK9^liver*(+/−)*^ mice showed a decrease in plasma TC levels by 29.7% (*P* < 0.001) and 23.4% (*P* < 0.05), respectively, as compared to PCSK9^liver*(+/+)*^ mice (Figure 1I). Consistent with this finding, ÄKTA-FPLC revealed a reduction in TC levels in LDL and HDL particles in hepatic PCSK9 knockdown mice, particularly in PCSK9^liver*(−/−)*^ mice (Figure 1K and Supplementary Material S2). However, the groups showed no significant differences in plasma TG levels (Figure 1J). Additionally, H & E staining revealed no apparent alterations in the liver among the groups (Figure 1L and Supplementary Material S3). The Oil Red O staining areas increased by 28.6% and 19.7%, respectively, in the liver sections of the PCSK9^liver*(−/−)*^ and PCSK9^liver*(+/−)*^mice as compared to those of the PCSK9^liver*(+/+)*^ mice (Figures 1M, N, Supplementary Material S3). This finding was consistent with the higher levels of TG accumulation in the liver of hepatic PCSK9^liver*(−/−)*^ mice as compared with those of PCSK9^liver*(+/+)*^ mice (Figure 1H).

### Effects of conditional knockdown of hepatic PCSK9 on hyperlipidemia-induced liver inflammation and the TLR-2/4 signaling pathway

**FIGURE 2:**
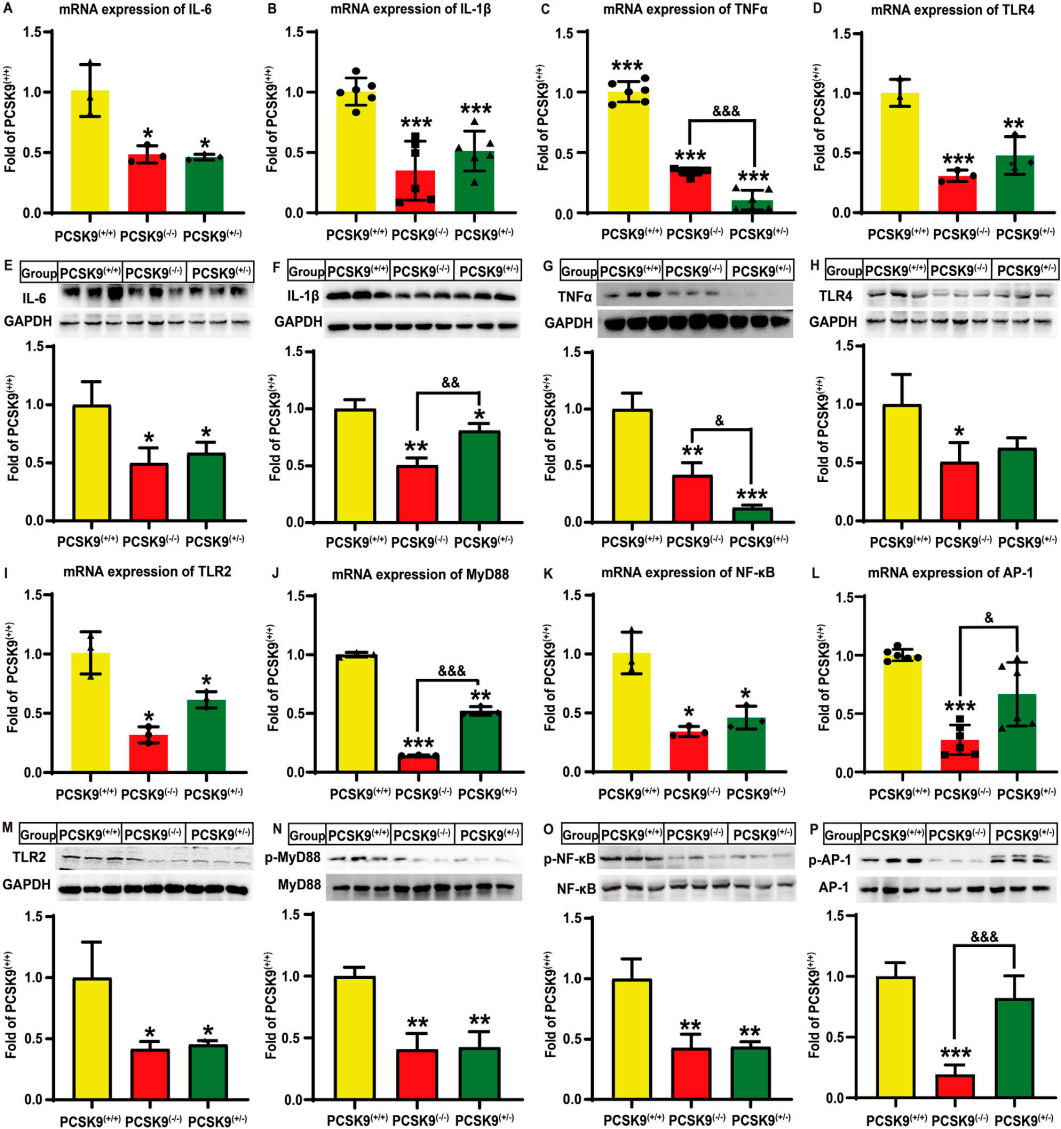
Effects of conditional knockdown of hepatic PCSK9 on some hepatic inflammation-related molecules in mice fed an HFD. Expression of **(A)**, IL-6 (n = 3); **(B)**, IL-1β (n = 6); **(C)**, TNFα (n = 6); and **(D)**, TLR4 (n = 3). Levels of **(E)**, IL-6; **(F)**, IL-1β; **(G)**, TNFα; and **(H)**, TLR4 proteins (n = 6). Expression of **(I)**, TLR2 (n = 3); **(J)**, MyD88 (n = 3); **(K)**, NF-κB (n = 3); and **(L)**, AP-1 (n = 6) proteins. Levels of **(M)**, TLR2; **(N)**, phosphorylated MyD88; **(O)**, phosphorylated p65-NF-κB; and **(P)**, phosphorylated AP-1 proteins (n = 6). ^*^means *p* < 0.05 vs*.* PCSK9^liver*(+/+)*^ group; ^**^means *p* < 0.01 vs*.* PCSK9^liver*(+/+)*^ group; ^***^means *p* < 0.001 vs*.* PCSK9^liver*(+/+)*^ group; ^&^means *p* < 0.05 vs*.* PCSK9^liver*(+/−)*^ group; ^&&^means *p* < 0.01 vs*.* PCSK9^liver*(+/−)*^ group; ^&&&^means *p* < 0.001 vs*.* PCSK9^liver*(+/−)*^ group.

Next, we assessed whether hepatic PCSK9 affects hyperlipidemia-induced expression of proinflammatory cytokines in mice liver. Notably, conditional knockdown of hepatic PCSK9 remarkably decreased the mRNA expression levels of IL-6, IL-1β, and TNFα by 51.4% (*P* < 0.05), 65.6% (*P* < 0.001), and 66.1% (*P* < 0.001), respectively, in PCSK9^liver*(−/−)*^ mice as compared to those in PCSK9^liver*(+/+)*^ mice (Figures 2A–C). Consistent with this finding, PCSK9^liver*(−/−)*^ mice showed a decrease in IL-6, IL-1β, and TNFα protein levels by approximately 50.3% (*P* < 0.05), 49.7% (*P* < 0.01), and 58.3% (*P* < 0.01), respectively, as compared to PCSK9^liver*(+/+)*^ mice (Figures 2E–G). Moreover, *IL-6*, *IL-1*β, and *TNFα* expression levels were reduced by 54.4% (*P* < 0.05), 49.3% (*P* < 0.001), and 89.7% (*P* < 0.001), respectively, in PCSK9^liver*(+/−)*^ mice as compared to those in PCSK9^liver*(+/+)*^ mice (Figures 2A–C). In line with these changes, PCSK9^liver*(+/−)*^ mice exhibited a similar reduction in IL-6, IL-1β, and TNFα protein levels as compared to PCSK9^liver*(−/−)*^ mice (Figures 2E–G). Notably, PCSK9^liver*(−/−)*^ mice showed a further reduction in the IL-1β protein level as compared to PCSK9^liver*(+/−)*^ mice (*P* < 0.01, Figure 2F). However, PCSK9^liver*(+/−)*^ mice showed a significant reduction in TNFα mRNA (*P* < 0.001, Figure 2C) and protein expression levels (*P* < 0.05, Figure 2G) as compared to PCSK9^liver*(−/−)*^ mice.

Given the important roles of TLR in inflammation, we investigated whether this signaling pathway was involved in the abovementioned process. Notably, hyperlipidemia stimulation significantly decreased the mRNA expression levels of *TLR-4* (Figure 2D) and *TLR-2* (Figure 2I) by 69.1% (*P* < 0.001) and 68.1% (*P* < 0.05), and 52.1% (*P* < 0.01) and 38.6% (*P* < 0.05), respectively, in PCSK9^liver*(−/−)*^ and PCSK9^liver*(+/−)*^ mice as compared to that of PCSK9^liver*(+/+)*^ mice. Although both PCSK9^liver*(−/−)*^ and PCSK9^liver*(+/−)*^ mice groups exhibited a reduction in TLR-4 protein levels, only the former group showed a significant difference in the TLR-4 protein level as compared to the PCSK9^liver*(+/+)*^ group (*P* < 0.05, Figure 2H). Conditional knockdown of hepatic PCSK9 also significantly reduced the TLR-2 protein level by approximately 58.3% and 54.8%, respectively, in both PCSK9^liver*(−/−)*^ and PCSK9^liver*(+/−)*^ mice groups (*P* < 0.05, Figure 2M). Moreover, PCSK9^liver*(−/−)*^ and PCSK9^liver*(+/−)*^ mice groups showed a reduction in *MyD88* expression levels by 85.8% (*P* < 0.001) and 47.8% (*P* < 0.01), respectively, as compared to PCSK9^liver*(+/+)*^ mice, with a significant difference between hepatic PCSK9^liver*(−/−)*^ and PCSK9^liver*(+/−)*^ mice groups (*P* < 0.001, Figure 2J). The phosphorylation levels of MyD88 were also decreased by approximately 59% in both PCSK9^liver*(−/−)*^ and PCSK9^liver*(+/−)*^ mice groups as compared to that in the PCSK9^liver*(+/+)*^ group (*P* < 0.01, Figure 2N). Importantly, conditional knockdown of hepatic PCSK9 significantly reduced the gene expression (*P* < 0.05) and phosphorylation levels of p65-NF-κB (*P* < 0.01) as compared to that in PCSK9^liver*(+/+)*^ mice (Figures 2K, O). Additionally, the *AP-1* gene expression level was decreased by approximately 72.6% in PCSK9^liver*(−/−)*^ mice, but not in PCSK9^liver*(+/−)*^ mice, as compared to that in PCSK9^liver*(+/+)*^ mice (*P* < 0.001, Figure 2L). Consistent with this observation, PCSK9^liver*(−/−)*^ mice exhibited a significant reduction in the phosphorylation level of the AP-1 protein as compared to PCSK9^liver*(+/+)*^ or PCSK9^liver*(+/−)*^ mice (*P* < 0.001, Figure 2P).

### Effects of conditional knockdown of hepatic PCSK9 on MAPK and PI3K signaling pathways

**FIGURE 3:**
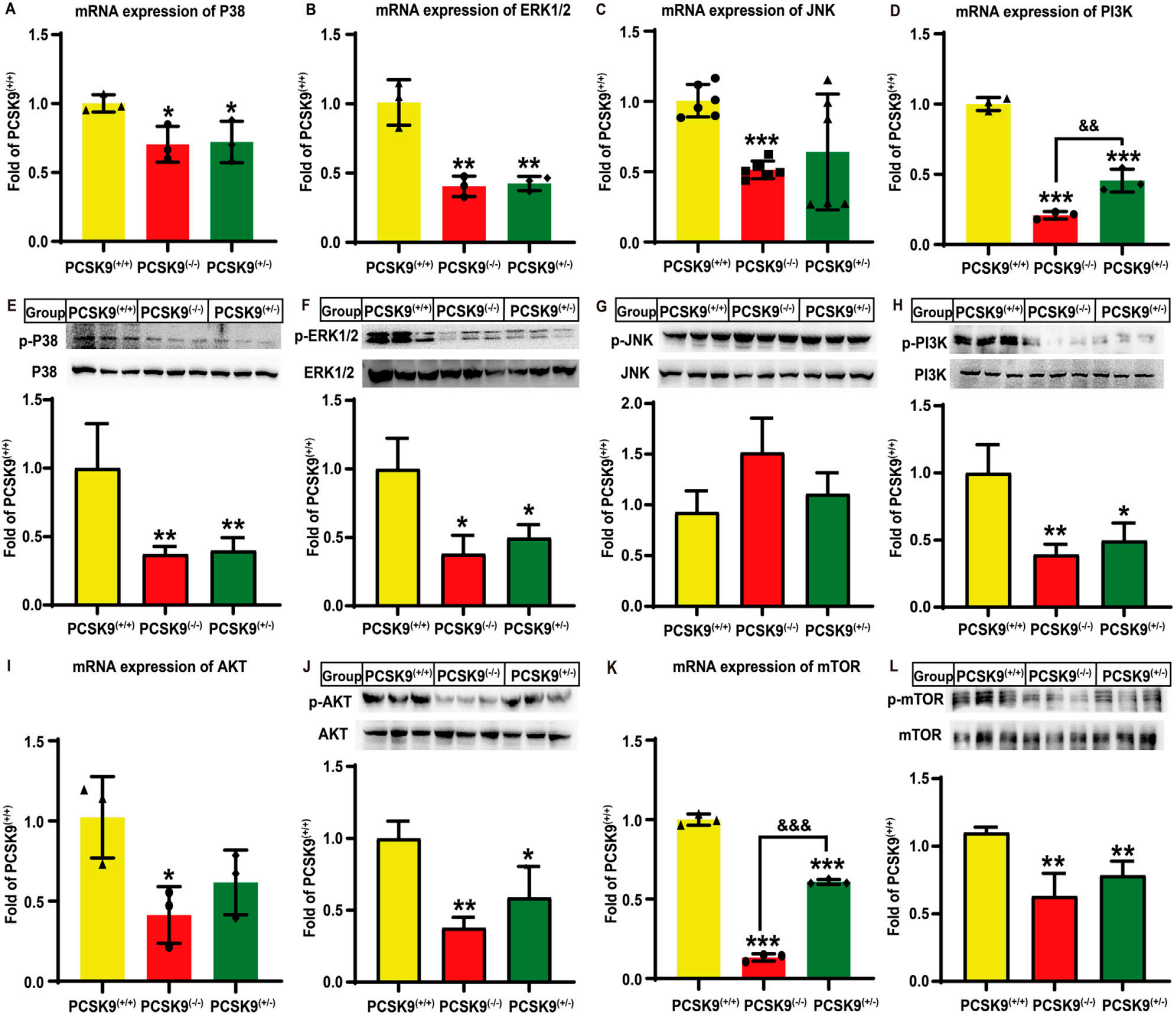
Effects of conditional knockdown of hepatic PCSK9 on some molecules involved in the MAPK and PI3K/AKT signaling pathways in mice fed an HFD. Expression of **(A)**, p38-MAPK (n = 3); **(B)**, ERK1/2 (n = 3); **(C)**, JNK (n = 6); and **(D)**, PI3K (n = 3). Phosphorylated levels of **(E)**, p38-MAPK; **(F)**, ERK1/2; **(G)**, JNK; and **(H)**, PI3K proteins (n = 6). Expression of **(I)**, AKT; and **(K)**, mTOR (n = 3). Phosphorylated levels of **(J)**, AKT; and **(L)**, mTOR proteins (n = 6). ^*^means *p* < 0.05 vs*.* PCSK9^liver*(+/+)*^ group; ^**^means *p* < 0.01 vs*.* PCSK9^liver*(+/+)*^ group; ^***^means *p* < 0.001 vs*.* PCSK9^liver*(+/+)*^ group; ^&&^means *p* < 0.01 vs*.* PCSK9^liver*(+/−)*^ group; ^&&&^means *p* < 0.001 vs*.* PCSK9^liver*(+/−)*^ group.

Next, we investigated the alterations in hepatic MAPK and PI3K/AKT signaling pathways following hyperlipidemia challenge. As shown in Figures 3A–C, the PCSK9^liver*(−/−)*^ mice group showed significant reductions in the mRNA expression of *p38-MAPK*, *ERK1/2*, and *JNK* by 29.5% (*P* < 0.05), 59.6% (*P* < 0.01), and 49.2% (*P* < 0.001), respectively, as compared to that in the PCSK9^liver*(+/+)*^ mice group. There were similar reductions in the expression of *p38-MAPK* and *ERK1/2*, but not *JNK*, in the PCSK9^liver*(+/−)*^ mice group (*P* < 0.05). Moreover, in both PCSK9^liver*(+/−)*^ and PCSK9^liver*(−/−)*^ mice groups, the levels of phosphorylated p38-MAPK and ERK1/2 proteins were downregulated by approximately 60% (*P* < 0.01, Figure 3E) and 50% (*P* < 0.05, Figure 3F), respectively, as compared to those in the PCSK9^liver*(+/+)*^ mice group. However, the levels of phosphorylated JNK exhibited no significant alterations among the mice groups (Figure 3G). Moreover, in PCSK9^liver*(−/−)*^ mice, the gene expression of *PI3K* and *AKT* was decreased by 79.1% (*P* < 0.001) and 58.6% (*P* < 0.05), respectively, as compared to that in PCSK9^liver*(+/+)*^ mice (Figures 3D, I). Consistent with this finding, in PCSK9^liver*(−/−)*^ mice, the levels of phosphorylated PI3K and AKT proteins were downregulated by 60.8% (*P* < 0.01, Figure 3H) and 62.3% (*P* < 0.01, Figure 3J), respectively, as compared to those in PCSK9^liver*(+/+)*^ mice. Moreover, the mRNA expression of *PI3K* and *AKT* in PCSK9^liver*(+/−)*^ mice was downregulated by 54.4% (*P* < 0.001) and 38.3%, respectively, as compared to that in PCSK9^liver*(+/+)*^ mice. Consistent with this finding, the levels of phosphorylated PI3K and AKT proteins in PCSK9^liver*(+/−)*^ mice were decreased by 50.4% and 41.0%, respectively, as compared to those of PCSK9^liver*(+/+)*^ mice (*P* < 0.05, Figures 3H, J). Additionally, PCSK9^liver*(−/−)*^ and PCSK9^liver*(+/−)*^ mice showed a reduction in mTOR expression levels by 86.7% (*P* < 0.001, Figure 3K) and 42.7% (*P* < 0.01, Figure 3L), respectively, as compared to that in PCSK9^liver*(+/+)*^ mice. Consistent with this finding, the levels of phosphorylated mTOR protein were downregulated by approximately 39.2% (*P* < 0.001, Figure 3K) and 28.7% (*P* < 0.01, Figure 3L) in PCSK9^liver*(−/−)*^ and PCSK9^liver*(+/−)*^ mice, respectively.

### Effects of PCSK9 siRNA treatment on the inflammatory state of lipid-loaded HepG2 cells

**FIGURE 4:**
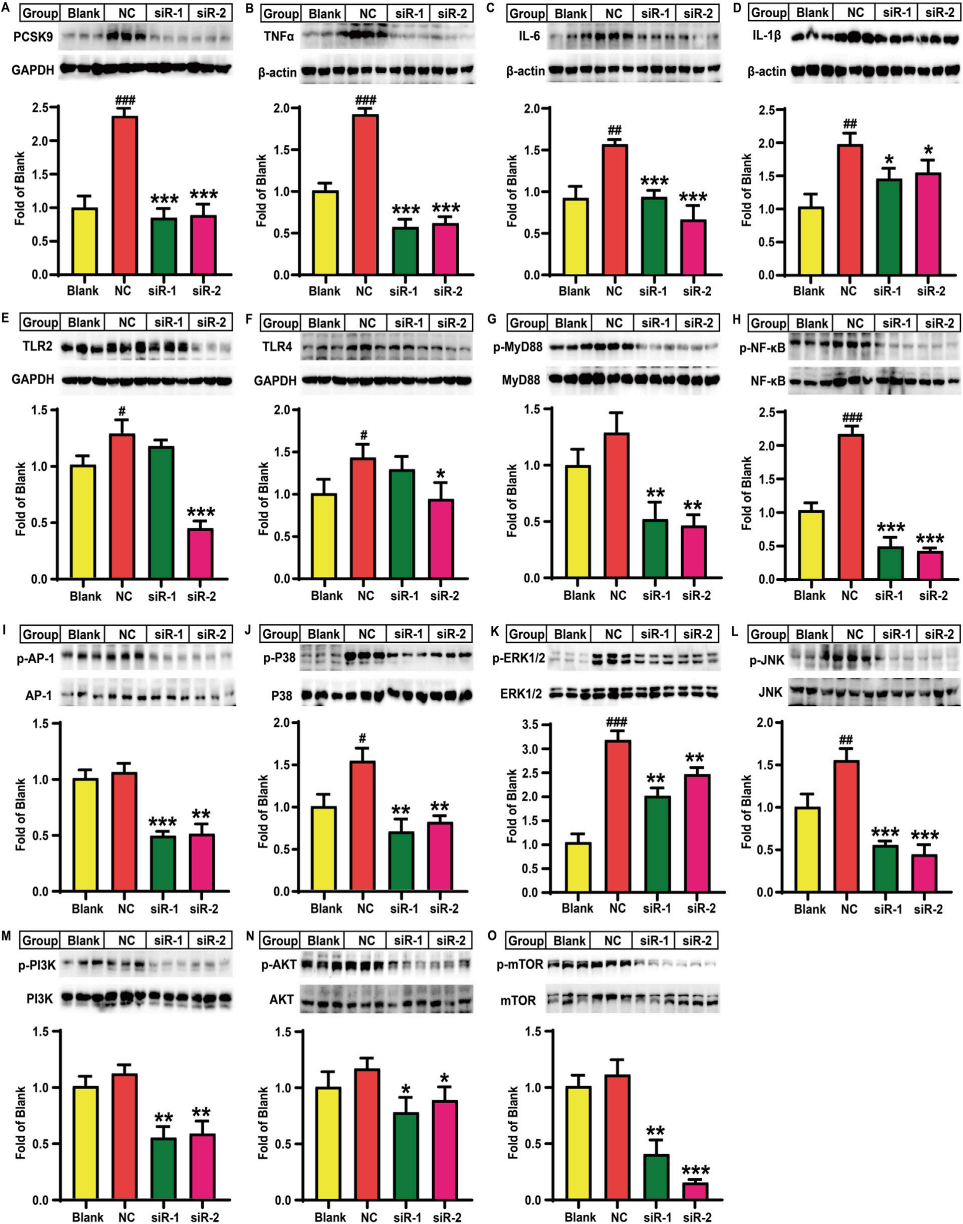
Effects of PCSK9 siRNA on the levels of some key molecules involved in lipid accumulation induced inflammation in HepG2 cells (n = 3). Levels of **(A)**, PCSK9; **(B)**, TNFα; **(C)**, IL-6; and **(D)**, IL-1β; **(E)**, TLR2; and **(F)**, TLR4 proteins. Phosphorylated levels of **(G)**, MyD88; **(H)**, p65-NF-κB; **(I)**, AP-1; **(J)**, p38-MAPK; **(K)**, ERK1/2; **(L)**, JNK; **(M)**, PI3K; **(N)**, AKT; and **(O)**, mTOR proteins. Blank: normal HepG2 cells; NC: lipid-loaded HepG2 cells were treated with negative control siRNA; siR-1: lipid-loaded HepG2 cells were treated with PCSK9 siRNA1; siR-2: lipid-loaded HepG2 cells were treated with PCSK9 siRNA2. ^#^means *p* < 0.05 vs*.* Blank group; ^##^means *p* < 0.01 vs*.* Blank group; ^###^means *p* < 0.001 vs*.* Blank group; ^*^means *p* < 0.05 vs*.* NC group; ^**^means *p* < 0.01 vs*.* NC group; ^***^means *p* < 0.001 vs*.* NC group.

Firstly, we demonstrated that the TC and TG levels in HepG2 cells were significantly increased after combined cis-9-octadecenoic acid and palmitic acid stimulation for 24 h, suggesting that a lipid-laden model was successfully established (Supplementary Figures S3A, B). Next, we demonstrated that lipid-loaded HepG2 cells exhibited higher levels of PCSK9, TNFα, IL-1β, IL-6, TLR-2, and TLR-4, and phosphorylated levels of NF-κB, MyD88, p38-MAPK, ERK1/2, JNK, PI3K, AKT, and mTOR proteins as compared to the blank control group. These findings suggested that lipid accumulation promoted inflammation in lipid-loaded HepG2 cells by regulating multiple signaling pathways as observed in mice. Importantly, there were no significant differences between the model and negative control groups, suggesting that the addition of control plasmid could not affect the levels of these proteins in lipid-loaded HepG2 cells (Supplementary Figure S3C–M and Supplementary Material S1). In the following, we investigated the effect of PCSK9 knockdown on lipid-loaded HepG2 cells and the underlying mechanisms of action. Lipid accumulation markedly increased the PCSK9 protein level by 136.2% (*P* < 0.001) in the NC group, while siR-1 and siR-2 intervention groups significantly decreased the PCSK9 protein level (*P* < 0.001, Figure 4A), as compared to that in the blank control group; this finding confirmed the effectiveness of these two PCSK9 siRNAs. Notably, TNFα, IL-6, and IL-1β protein levels were remarkably increased in the NC group by approximately 91.3% (*P* < 0.001), 70.4% (*P* < 0.01), and 92.3% (*P* < 0.01), respectively, as compared to those in the blank group, thus suggesting that lipid accumulation induced an inflammatory state in HepG2 cells. Compared to the NC group, the siR-1 intervention group showed a marked reduction in TNFα, IL-6, and IL-1β protein levels by 70.6% (*P* < 0.001), 40.6% (*P* < 0.001), and 26.5% (*P* < 0.05), respectively, while the siR-2 intervention group reduced their levels by 68.2% (*P* < 0.001), 57.9% (*P* < 0.001), and 21.8% (*P* < 0.05), respectively (Figures 4B–D). Lipid accumulation also elevated the TLR-2 and TLR-4 protein levels by approximately 40.0% and 53.5%, respectively, in the NC group (*P* < 0.05, Figures 4E, F). siR-2 alone significantly decreased TLR-2 and TLR-4 protein levels by 66.1% (*P* < 0.001) and 34.5% (*P* < 0.05), respectively. Although the NC group showed an increase by approximately 29.1% in the MyD88 protein phosphorylation level, this increase was not significant as compared to that in the blank group. In contrast, both siR-1 and siR-2 treatment significantly reduced MyD88 phosphorylation levels by approximately 60% as compared to that in the NC group (*P* < 0.01, Figure 4G). Notably, lipid accumulation in HepG2 cells significantly increased the p65 NF-κB protein phosphorylation level by 111.1% (*P* < 0.001, Figure 4H) without affecting AP-1 protein phosphorylation level as compared to that in the blank group (Figure 4I). However, transfection with siR-1 markedly decreased p65 NF-κB and AP-1 protein phosphorylation levels by approximately 77.6% (*P* < 0.001) and 54.3% (*P* < 0.001), respectively, while transfection with siR-2 decreased their levels by 80.9% (*P* < 0.001) and 52.4% (*P* < 0.01), respectively (Figures 4H, I), as compared to those in the NC group.

Additionally, as observed in the NC group, lipid accumulation significantly elevated p38-MAPK, ERK1/2, and JNK protein phosphorylation levels by 54.4% (*P* < 0.05), 208.1% (*P* < 0.001), and 55.5% (*P* < 0.01), respectively, as compared to the blank control group (Figures 4J–L). In contrast, as compared to the NC group, both siR-1 and siR-2 for PCSK9 significantly decreased p38-MAPK, ERK1/2, and JNK protein phosphorylation levels by more than 47% (*P* < 0.01), 23% (*P* < 0.01), and 65% (*P* < 0.001), respectively (Figures 4J–L). Lipid accumulation did not alter PI3K, AKT, and mTOR protein phosphorylation levels as compared to those in the blank control group (Figures 4M–O). However, as compared to the NC group, the siR-1 and siR-2 intervention groups showed a significant reduction in PI3K protein phosphorylation levels by 51.3% and 48.1%, respectively (*P* < 0.01, Figure 4M). siR-1 and siR-2 for PCSK9 also remarkably decreased the AKT protein phosphorylation levels by approximately 33.6% and 24.1%, respectively (*P* < 0.05, Figure 4N). Additionally, the mTOR protein phosphorylation levels in the siR-1 and siR-2 treatment groups were reduced by 64.1% (*P* < 0.01) and 87.3% (*P* < 0.001), respectively, as compared to those in the NC group (Figure 4O).

### Effects of anti-PCSK9 antibody and/or p38-MAPK inhibitor on lipid-laden induced inflammation in HepG2 cells

**FIGURE 5:**
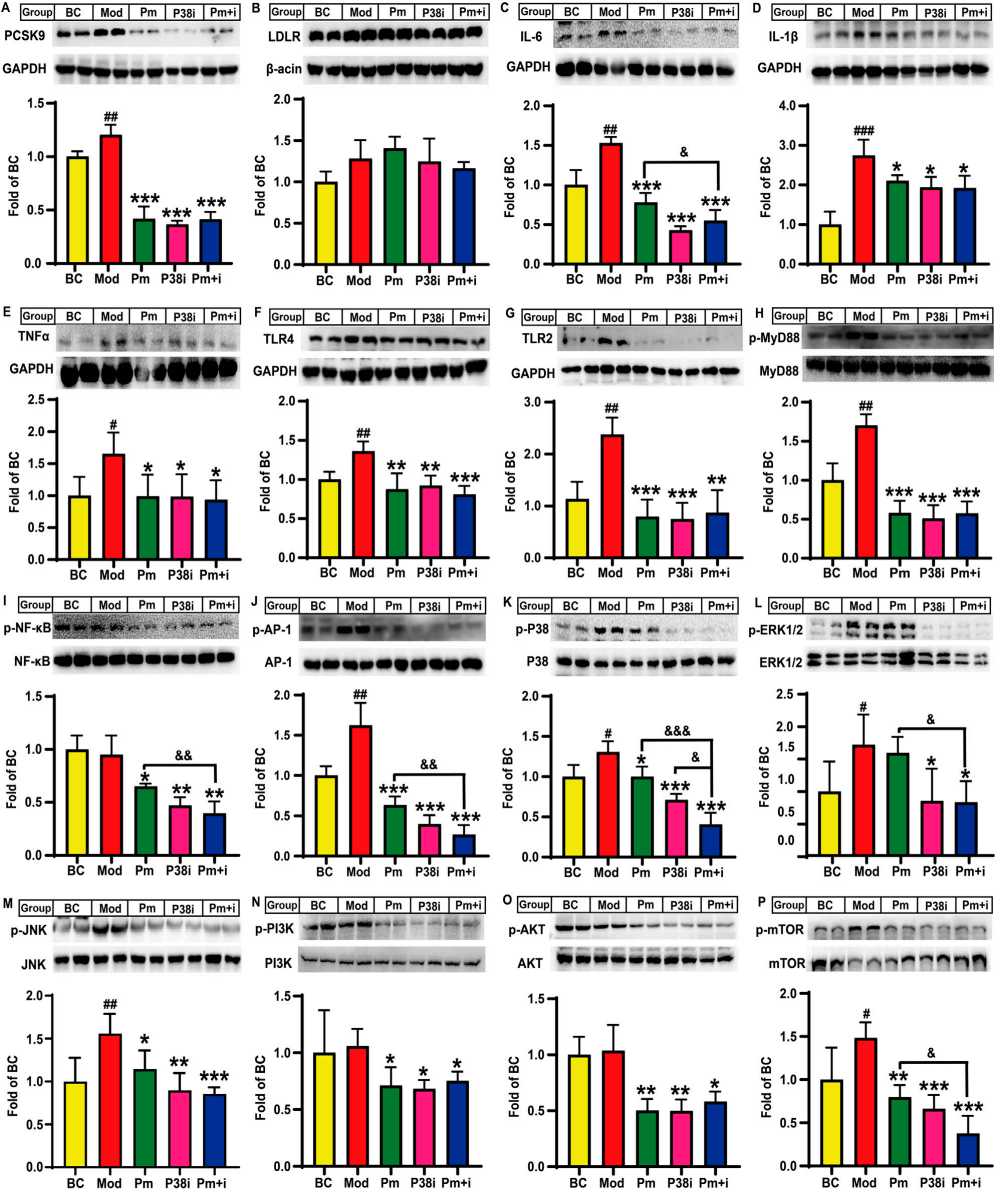
Effects of alirocumab and SB203580 on PCSK9, LDLR, and some key molecules involved in lipid accumulation induced inflammation in HepG2 cells (n = 4). Levels of **(A)**, PCSK9; **(B)**, LDLR; **(C)**, IL-6; **(D)**, IL-1β; **(E)**, TNFα; **(F)**, TLR4; and **(G)**, TLR2 proteins. Phosphorylated levels of **(H)**, MyD88; **(I)**, p65-NF-κB; **(J)**, AP-1; **(K)**, p38-MAPK; **(L)**, ERK1/2; **(M)**, JNK; **(N)**, PI3K; **(O)**, AKT; and **(P)**, mTOR proteins. BC: blank control; Mod: model, lipid-loaded HepG2 cells; Pm: lipid-loaded HepG2 cells were treated with alirocumab (10 μg/mL); p38i: lipid-loaded HepG2 cells were treated with SB203580 (30 µM); Pm + i: combination group, lipid-loaded HepG2 cells were treated with PCSK9 alirocumab (10 μg/mL) and SB203580 (30 µM). ^#^means *p* < 0.05 vs*.* BC group; ^##^means *p* < 0.01 vs*.* BC group; ^###^means *p* < 0.001 vs*.* BC group; ^*^means *p* < 0.05 vs*.* Mod group; ^**^means *p* < 0.01 vs*.* Mod group; ^***^means *p* < 0.001 vs*.* Mod group; ^&^means *p* < 0.05 vs*.* Pm + i group; ^&&^means *p* < 0.01 vs*.* Pm + i group; ^&&&^means *p* < 0.001 vs*.* Pm + i group.

Finally, we investigated the role of PCSK9 in lipid-laden HepG2 cells and the underlying mechanisms of action. As shown in Figure 5A, lipid accumulation increased PCSK9 protein levels by 20.5% (*P* < 0.01) in HepG2 cells, while treatment of these cells with the anti-PCSK9 antibody alirocumab or the p38-MAPK inhibitor SB203580 alone or in combination significantly decreased PCSK9 protein levels by 65.3%, 69.6%, and 65.8%, as compared those in the model group (*P* < 0.001). Furthermore, compared to the blank control group, the model group showed an increase in the LDLR protein levels by 28.3%; however, neither alirocumab nor SB203580 significantly increased the LDLR protein level (Figure 5B). Notably, lipid accumulation significantly increased IL-6, IL-1β, and TNFα protein levels in the model group by approximately 52.7% (*P* < 0.01), 174.6% (*P* < 0.001), and 65.3% (*P* < 0.05), respectively, in the model group as compared to those in the blank control group (Figures 5C–E), while alirocumab or SB203580 alone or in combination attenuated these changes. A noteworthy finding is that the IL-6 protein levels in the combination group were significantly reduced as compared to that in the alirocumab alone intervention group (*P* < 0.05, Figure 5C). TLR-4 and TLR-2 protein levels were significantly increased by 36.1% and 137.6%, respectively, in lipid-loaded HepG2 cells as compared to those in the blank control group (*P* < 0.01, Figures 5F,G). Intervention with anti-PCSK9 antibody significantly decreased TLR-4 level (∼35.7%, *P* < 0.01) and particularly TLR-2 level (∼66.6%, *P* < 0.001) in lipid-laden HepG2 cells. SB203580 exhibited similar effects as those observed for alirocumab. However, alirocumab addition following SB203580 intervention did not further decrease TLR-4 or TLR-2 protein levels. Moreover, lipid accumulation significantly increased the phosphorylated MyD88 protein level by 70.2% (*P* < 0.01), while drug intervention significantly reversed this increase by approximately 65.9% (*P* < 0.001, Figure 5H). Importantly, alirocumab or SB203580 alone or in combination significantly decreased p65-NF-κB phosphorylation by approximately 36% (*P* < 0.05, Figure 5I) and reduced the phosphorylation levels of AP-1 by 61.0%, 75.5%, and 83.5%, respectively, as compared to those in the model group (*P* < 0.001, Figure 5J). The combination group showed a further decrease in the phosphorylated AP-1 protein level by 57.8% as compared to that in alirocumab alone intervention group (*P* < 0.01, Figure 5J).

Lipid accumulation also significantly increased the phosphorylation levels of p38-MAPK (∼30.6%, *P* < 0.05, Figure 5K), ERK1/2 (∼56.6%, *P* < 0.05, Figure 5L), and JNK (∼59.9%, *P* < 0.01, Figure 5M) proteins, as compared to those of the blank control group. Furthermore, the p38-MAPK and JNK proteins, but not ERK1/2 protein, showed a decrease in the phosphorylation levels by 23.5% (*P* < 0.05) and 26.6% (*P* < 0.05), respectively, in the alirocumab alone intervention group as compared to that in the model group. As a positive control, SB203580 significantly reduced p38-MAPK, ERK1/2, and JNK phosphorylation levels by approximately 46.1% (*P* < 0.001, Figure 5K), 45.4% (*P* < 0.05, Figure 5L), and 42.5% (*P* < 0.01, Figure 5M), respectively, as compared to those in the model group. Notably, the combination group also exhibited a decrease in the p38-MAPK phosphorylation level as compared to the alirocumab alone intervention group (*P* < 0.001, Figure 5K). Moreover, alirocumab further reduced the p38-MAPK protein phosphorylation level by 42.9% following SB203580 administration (*P* < 0.05, Figure 5K); this finding suggests that the anti-PCSK9 antibody alirocumab showed a synergistic effect with the p38-MAPK inhibitor SB203580. Notably, the combination group displayed the best effect in reducing the JNK phosphorylation level as compared to the model group (∼45.1%, *P* < 0.001, Figure 5M). Additionally, alirocumab or SB203580 alone or in combination remarkably decreased the phosphorylation levels of PI3K (∼30%, *P* < 0.05) and AKT (∼50%, *P* < 0.05) as compared to that in the model group (Figures 5N, O). Alirocumab, however, did not show a synergistic effect with SB203580 for reducing PI3K and AKT levels. Lipid accumulation in HepG2 cells also elevated the mTOR protein phosphorylation level by approximately 48.3% (*P* < 0.05) as compared to that in the blank control. In contrast, alirocumab, SB203580, or their combination significantly decreased the mTOR protein phosphorylation levels by 46.2% (*P* < 0.01), 55.3% (*P* < 0.001), and 74.6% (*P* < 0.001), respectively, as compared to those in the model group (Figure 5P). Furthermore, the combination intervention decreased the mTOR protein phosphorylation levels by 52.9% as compared to those in the alirocumab alone intervention group (*P* < 0.05, Figure 5P).

## Discussion

Recent studies have revealed that a positive association of PCSK9 with vascular inflammation (Tang et al., 2017; Yurtseven et al., 2020). Although PCSK9 deficiency in the liver elevates hepatic CD36-mediated fatty acid uptake, which increases the risk of liver injury and the expression of hepatic inflammatory factors, including IL-6, IL-1β, and TNFα (Demers et al., 2015; Lebeau et al., 2019), the effect of PCSK9 knockdown rather than its complete knockout on liver inflammation remains unclear. Here, we demonstrated that conditional knockdown of hepatic PCSK9 significantly decreased hyperlipidemia-induced gene expression and protein levels of some key pro-inflammatory factors potentially via downregulating several signaling pathways including TLR/MyD88, MAPK, and PI3K/AKT; this phenomenon further decreased the phosphorylation levels of NF-κB and AP-1 proteins in the nuclei, resulting in a reduction in inflammatory reaction.

First, we successfully obtained PCSK9^liver*(−/−)*^, PCSK9^liver*(+/−)*^ and PCSK9^liver*(+/+)*^ mice as confirmed by genotyping experiments. RT-qPCR and Western blotting assay showed a significant reduction in the gene expression and protein levels of PCSK9 in both PCSK9^liver*(−/−)*^ and PCSK9^liver*(+/−)*^ mice as compared to those in PCSK9^liver*(+/+)*^ mice. In line with these observations, as a post-translational LDLR regulator, hepatic PCSK9 knockdown apparently increased the LDLR protein level without affecting the gene expression of LDLR; these findings agreed with previously reported results (Maxwell and Breslow, 2004; Zhang et al., 2007; Zaid et al., 2008; Demers et al., 2015). In contrast, the downregulation of PCSK9 did not induce a significant increase in the LDLR protein level in HepG2 cells. Mechanistically, PCSK9 suppression has been demonstrated to downregulate SREPB-2 *in vitro*, a master regulator of LDLR and PCSK9 (Marques et al., 2022). Furthermore, the mice experimental groups showed no significant differences in body weight, liver indices, and hepatic TC levels. In line with these findings, the body weight and hepatic TC levels of PCSK9^liver*(+/+)*^ and PCSK9^liver*(−/−)*^ mice exhibited no significant difference after an HFD challenge for 12 weeks. However, PCSK9^*(−/−)*^ mice displayed a significant increase in the liver indices accompanied with elevated levels of hepatic TG (approximately 90%) (Lebeau et al., 2019), and the hepatic TG levels were increased by 57.8% in PCSK9^liver*(−/−)*^ mice as compared to that in PCSK9^liver*(+/+)*^ mice due to elevated LDLR-mediated uptake of lipoprotein particles, especially non-HDL particles. These differences might be induced by the distinct challenge time (9 weeks vs. 12 weeks) and the difference in the hepatic PCSK9 levels (knockdown vs. knockout) between our study and the study of Lebeau (Lebeau et al., 2019). In hepatic-specific PCSK9 knockdown mice, the levels of hepatic PCSK9 proteins can be maintained at a certain level due to the following reasons. (1) Although PCSK9 is primarily expressed in the liver, this secretary protein is also generated by extrahepatic organs including colon, ileum, duodenum, and kidneys (Zaid et al., 2008); PCSK9 secreted by extrahepatic organs is transferred from the bloodstream to the liver. (2) Alb-Cre specifically target hepatocyte to knockout PCSK9, the rest cells, including hepatobiliary duct cell and Kupffer cell in the liver, still express PCSK9. These facts may explain why we did not observe significant differences in the levels of hepatic PCSK9 protein between PCSK9^liver*(−/−)*^ and PCSK9^liver*(+/−)*^ mice.

Second, we demonstrated that conditional knockdown of hepatic PCSK9 reduced hyperlipidemia-induced production of several pro-inflammatory cytokines (Figure 2, Supplementary Material S2, S3). Consistent with these findings, accumulating evidence has demonstrated that hyperlipidemia causes inflammation (Ma and Feng, 2016; Miao et al., 2023). For instance, familial hypercholesterolemia induces low-grade systemic inflammation, thereby promoting the onset and development of atherosclerosis, a chronic inflammatory disease. Anti-PCSK9 antibodies, such as alirocumab, reduce the levels of inflammatory cytokines in hypercholesterolemic patients (Marques et al., 2022; Seidah and Garçon, 2022). A Mendelian randomization study showed a positive association between the plasma TG levels and the increased risk of MAFLD (Chen et al., 2022). In the present study, the trend of reduction in plasma TC levels in PCSK9^liver*(−/−)*^ and PCSK9^liver*(+/−)*^ mice were consistent with previous findings (Zaid et al., 2008; Meng et al., 2023). As LDLR binds both apolipoprotein B and apolipoprotein E, conditional knockdown of hepatic PCSK9 not only reduced LDL-c levels but also decreased HDL-c levels, which were consistent with previous studies (Zaid et al., 2008; Meng et al., 2023). Importantly, both PCSK9^liver*(−/−)*^ and PCSK9^liver*(+/−)*^ mice exhibited a marked decrease in the gene expression and protein levels of some hepatic pro-inflammatory cytokines such as IL-6, IL-1β, and TNFα. This reduction could be partially attributed to the decrease in circulating TC levels. However, the downregulation degree of these proinflammatory factors exhibited differences in PCSK9^liver*(−/−)*^ and PCSK9^liver*(+/−)*^ mice. For instance, the expression of TNFα was lower in PCSK9^liver*(+/−)*^ mice as compared to that in PCSK9^liver*(−/−)*^ mice, this may be partially attributed to the higher levels of hepatic TG in the latter. As the expression of these proinflammatory factors are modulated by a complex network, we cannot precisely explain these differences. In a mouse model of fibrosis, PCSK9 deletion alleviated hepatic inflammation, accompanied with a reduction in the plasma levels of lipopolysaccharide (LPS), aspartate aminotransferase (AST), and alanine aminotransferase (ALT), which are representative biomarkers of liver injury (Zou et al., 2020). Anti-PCSK9 monoclonal antibodies also lowered serum ALT and AST levels in some preclinical and clinical studies (Momtazi-Borojeni et al., 2022). Here, we found that alirocumab inhibited inflammation in lipid-loaded HepG2 cells. In another study, PCSK9 transgenic mice exhibited higher levels of liver injury, while PCSK9-deficient mice showed opposite effects, thus suggesting PCSK9 deficiency protects organs against bacterial dissemination and inflammatory injury (Dwivedi et al., 2016). However, the related mechanisms are not yet known. Another noteworthy finding is that PCSK9 knockdown ameliorates aortic inflammation by inhibiting the TLR-4 signaling pathway without affecting the lipid profiles in apolipoprotein E-deficient mice (Tang et al., 2017). These findings imply that PCSK9 inhibition may directly suppress inflammation through mechanisms other than lowering of the lipid level.

Third, our results suggest that conditional knockdown of hepatic PCSK9 ameliorates hyperlipidemia-induced inflammation by downregulating multiple signaling pathways. Here, we showed that conditional knockdown of hepatic PCSK9 or intervention with anti-PCSK9 antibody significantly decreased TLR-4 expression levels both *in vivo* and *in vitro*. LPS is an endotoxin recognized by TLR-4 localized on the membrane of several cell types, including hepatocytes (Lee et al., 2021). Several previous studies suggest that PCSK9 knockdown inhibits TLR-4/Myd88/NF-κB signaling in the aorta of apolipoprotein E-deficient mice or rabbits (Tang et al., 2017; Liu et al., 2020). Mechanistically, the unique C-terminal cysteine-rich domain of PCSK9 is presumed to bind and activate TLR-4, thereby causing an inflammatory reaction (Tarkowski et al., 2010; Cheng et al., 2016; Tang et al., 2017). These findings indicate that PCSK9 in different organs or cells might induce inflammation by modulating of the same TLR-4 signaling pathway. In contrast, our results demonstrated that hepatic PCSK9 knockdown or inhibition particularly reduced TLR-2 level rather than TLR-4 level, with inhibition of the downstream NF-κB and AP-1 signaling pathways, thus suggesting PCSK9 suppression may stimulate different TLR family members following hyperlipidemia challenge. Lipid accumulation may also induce other unknown proinflammatory factors, apart from LPS, which participate in TLR-2 activation. It is necessary to note that PCSK9^liver*(−/−)*^ and PCSK9^liver*(+/−)*^ mice exhibited different effect on suppressing the gene expression and protein levels of phosphorylated AP-1, suggesting the slight differences in the levels of PCSK9 may play a key role in modulating this transcription factor. The downregulation of the proinflammatory factors in PCSK9^liver*(+/−)*^ mice may primarily attribute to the downregulation of NF-κB and other unknown transcription factors.

Notably, recent studies have indicated a relationship between PCSK9 and the MAPK signaling pathway. A microarray analysis suggested that a gain-of-function mutation in PCSK9 (D374Y) increases the expression of several signaling molecules involved in inflammation, including TNF-4, TNF receptor IIa, MAPK kinase, and NF-κB in HepG2 cells (Lan et al., 2010). Another study demonstrated that MAPKs, including ERK, JNK, and p38-MAPK, are involved in PCSK9 secretion, particularly in mice fed an HFD (Ding et al., 2020b). Our findings indicated that conditional knockdown of hepatic PCSK9 *in vivo* or PCSK9 suppression *in vitro* inhibited the phosphorylation of ERK1/2 and p38-MAPK proteins involved in the MAPK signaling pathway. However, PCSK9 suppression exhibited differences in modulation of JNK *in vivo* and *in vitro*. This may be explained by the different modulatory mechanisms because the *in vivo* situations are more complexed than those *in vitro*. Moreover, the alterations of the mRNA expression of these MAPKs were inconsistent with their protein levels, suggesting some unknown mechanisms are involved in the post-translational regulation of these proteins. For instance, the levels of protein are finely controlled by multiple factors, including the stability of mRNA, the function of ribosome, the levels of translation-related factors, and protein degradation (Wu and Bazzini, 2023; Jia et al., 2024). In this study, alirocumab further decreased the phosphorylation level of the p38-MAPK protein; however, it did not affect IL-6, IL-1β, and TNFα proinflammatory cytokine levels following treatment with the p38-MAPK inhibitor *in vitro*. This result indicates that the p38-MAPK signaling pathway may primarily contribute to PCSK9-mediated inflammation in lipid-loaded HepG2 cells. In addition to ERK1/2 and p38-MAPK proteins, JNK protein phosphorylation level was reduced by PCSK9 siRNAs, thus suggesting that PCSK9 siRNAs showed more robust effects in inhibiting the MAPK signaling pathway than alirocumab. Consistent with this observation, a recent study demonstrated that downregulation of the MAPK signaling pathway is a protective mechanism for MASH (Wu et al., 2023). All these findings suggest that the mechanisms of action of PCSK9 suppression on attenuating hyperlipidemia-induced inflammation are associated with downregulation of the MAPK signaling pathway.

Several previous studies have also indicated an association between the PI3K/AKT signaling pathway and PCSK9. For instance, PI3K/AKT signaling is involved in modulating the sterol regulatory element binding protein-2/PCSK9 signaling pathway in HepG2 cells (Xiao et al., 2019). Here, we found that conditional knockdown of hepatic PCSK9 suppressed the levels of phosphorylated PI3K, AKT, NF-κB, and AP-1 proteins following hyperlipidemia challenge; this result suggests that PCSK9 knockdown may reduce inflammation by inhibiting the PI3K/AKT signaling pathway. Another study also indicated that PCSK9 deficiency suppresses AKT protein phosphorylation in hyperlipidemic mice (Lebeau et al., 2019). mTOR, a downstream molecule of the PI3K/AKT signaling pathway, is involved in modulating autophagy, a cellular protective mechanism following unfavorable conditions such as inflammation (Yurtseven et al., 2020; Rajendran et al., 2024). Interestingly, PCSK9 inhibition promotes autophagy by suppressing phosphorylation of mTOR (Ding et al., 2016; Yurtseven et al., 2020). Our results demonstrated that hepatic PCSK9 knockdown *in vivo* or PCSK9 inhibition *in vitro* markedly reduced phosphorylated mTOR levels, thus suggesting that PCSK9 suppression may attenuate inflammation by improving autophagy. It is worth noting that HFD-induced upregulation of the PI3K/AKT signaling pathway in mice cannot be reproduced in lipid-loaded HepG2 cells potentially due to the distinct metabolic situations of these two models. However, both anti-PCSK9 antibody and siRNAs against PCSK9 significantly inhibited the PI3K/AKT/mTOR signaling pathway in HepG2 cells as observed in PCSK9^liver*(−/−)*^ and PCSK9^liver*(+/−)*^ mice.

Although the complete deletion of PCSK9, an extreme condition, enhanced CD36-mediated lipid accumulation and subsequent inflammation in mice (Demers et al., 2015; Lebeau et al., 2019), our findings revealed that conditional knockdown of hepatic PCSK9 *in vivo* and PCSK9 suppression *in vitro* were associated with decreased inflammation following lipid accumulation. These contrasting results might be due to the abovementioned reasons, including differences in the animal model (PCSK9-deficient model vs. conditional knockdown of hepatic PCSK9 model), HFD challenge period (12 weeks vs. 9 weeks), and the differences in the components of HFD. In our model, PCSK9 secreted by other organs is transferred to liver through blood flow and exert its effects, such as downregulation of CD36, thereby partially ameliorating hepatic lipid accumulation. Moreover, a shorter challenge period in our study may reduce the severity of hepatic lipid accumulation. These assumptions are consistent with the unaltered liver indices and less hepatic TG accumulation noted in our study. Consistent with this finding, PCSK9 overexpression or gain-of-function exhibited an association with MAFLD as revealed by laboratory and clinical studies (Lebeau et al., 2022). These observations suggest that PCSK9 modulation may induce different or even contrasting complementary mechanisms under distinct conditions. For instance, PCSK9-deficient mice exhibit higher levels of ATP-binding cassette transporter A1 and higher levels of cholesterol in the feces than control mice; this suggests that PCSK9 deletion may promote reverse cholesterol transport as a complementary mechanism (Lebeau et al., 2019). Moreover, PCSK9^*(−/−)*^ mice showed impairment of liver regeneration, while PCSK9^*(+/−)*^ mice exhibit an improved liver regeneration and resistance to hepatic steatosis (Momtazi-Borojeni et al., 2022); this finding indicates that a moderate level of the PCSK9 protein can protect mice against MAFLD and MASH. As discussed above, although hyperlipidemia causes inflammation, it remains unclear how excessive hepatic lipid accumulation may promote elevated production of proinflammatory factors, thereby inducing systemic inflammation (Ugovšek and Šebeštjen, 2022). An appropriate elevated level of hepatic TG accumulation may stimulate complementary anti-inflammatory mechanisms. It should also be noted that conditional knockdown of hepatic PCSK9 significantly decreased plasma TC levels, which are sure to ameliorate systemic inflammation and thereby reduce hepatic inflammation.

In conclusion, the results of the present study suggest that PCSK9 downregulation to ameliorates hyperlipidemia-induced liver inflammation without inducing sever lipid accumulation. The related mechanisms of action are associated with the downregulation of TLR-4, TLR-2, MAPK, and PI3K/AKT/mTOR as well as their downstream NF-κB and AP-1 signaling pathways. However, this study has several limitations as mentioned hereafter. Firstly, in the liver of PCSK9^liver*(+/−)*^ and PCSK9^liver*(−/−)*^ mice, there is PCSK9 expressed by other cells and transferred from the blood flow as discussed above. Therefore, complete knockout of hepatic PCSK9 is not available, and we only demonstrated that hepatic PCSK9 knockdown attenuates hyperlipidemia-induced liver inflammation. A comparative study is necessary to confirm the differences in HFD-induced chronic liver inflammation by using PCSK9 knockout and hepatic PCSK9 knockdown animal models. Secondly, HepG2 cells are derived from human liver carcinoma and their metabolic profiles often differ significantly from normal hepatocytes. Furthermore, the *in vivo* anti-inflammatory mechanisms are more complex that those *in vitro*. Therefore, some of the *in vivo* results in PCSK9 knockdown mice, including the regulation of the PI3K/AKT signaling pathway and PCSK9-mediated LDLR degradation, could not be reproduced in HepG2 cells in this study. Thus, it is necessary to determine whether siRNAs or antibodies against PCSK9 could reduce lipid accumulation induced inflammation in primary hepatocytes. Furthermore, we only investigated the anti-inflammatory effects of PCSK9 antibody (alirocumab) and siRNAs in HepG2 cells pre-loaded with lipids. Although this model is designed to mimic MAFLD therapy using PCSK9 inhibitors, these experiments cannot reflect the effects of PCSK9 suppression induced increase in LDLR-mediated lipid uptake, which might affect the final anti-inflammatory effects of PCSK9 inhibition. Additionally, the effects of PCSK9 on modulation of lipid metabolism in HepG2 cells, especially the underlying mechanisms of action are not well clarified. Thirdly, although we investigated the effects of conditional knockdown of hepatic PCSK9 on HFD-induced liver inflammation following 3-month intervention and the underlying mechanisms of action, this study cannot reflect the potential different effects of PCSK9 in the dynamic processes of hyperlipidemia-induced liver inflammation. Furthermore, as PCSK9^liver*(−/−)*^ mice with a minor higher level of liver TG exhibited equal or even stronger anti-inflammatory effects as compared to those in PCSK9^liver*(+/−)*^or PCSK9^liver*(+/+)*^ mice, it remains to be determined how excessive hepatic lipid accumulation may cause elevated liver inflammation or MASH in mice with conditional knockdown of hepatic PCSK9. Fourth, because multiple signaling pathways are involved in these processes, we could not presently confirm the contribution of each signaling pathway. Furthermore, whether PCSK9 differently regulate the JNK, p38-MAPK, and ERK signaling pathways requires further studies in the future.

## Data Availability

The original contributions presented in the study are included in the article/Supplementary Material, further inquiries can be directed to the corresponding author.

## Abbreviations

AKT, protein kinase B; ALT, alanine aminotransferase; AP-1, activator protein-1; AST, aspartate aminotransferase; CVD, cardiovascular disease; CD36, cluster of differentiation 36; DMEM, Dulbecco′s modified Eagle′s medium; ERK1/2, extracellular regulated protein kinase 1/2; FPLC, fast protein liquid chromatography; GAPDH, glyceraldehyde-3-phosphate dehydrogenase; HDL, high-density lipoprotein; IL, interleukin; JAK, janus kinase; JNK, c-Jun N-terminal kinase; LDLR, low-density lipoprotein receptor; LPS, lipopolysaccharide; MAFLD, metabolic dysfunction-associated fatty liver disease; MAPK, mitogen-activated protein kinase; mTOR, mammalian target of rapamycin; MyD88, myeloid differentiation factor 88; MASH, metabolic dysfunction-associated steatohepatitis; NF-κB, nuclear factor kappa-B; PCSK9, proprotein convertase subtilisin/kexin type 9; PI3K, phosphoinositide-3 kinase; PVDF, Polyvinylidene fluoride; TBS, Tris-buffered saline; TC, total cholesterol; TG, triglycerides; TLR, toll-like receptor; TNF, tumor necrosis factor.
