# Supplementary material for: Conditional knockdown of hepatic PCSK9 ameliorates high-fat diet-induced liver inflammation in mice
Source: Front Pharmacol. 2025 Feb 3;16:1528250. doi: 10.3389/fphar.2025.1528250 (PMC11830812; doi:10.3389/fphar.2025.1528250)
Supplement: Supplementary file 1 [file Presentation1.zip › Data Sheet 1/Supplementary material S1.pdf]

Repeated Figure 1C PCSK9 (From left to right, 1-3: PCSK9$^{(+/+)}$; 4-6: PCSK9$^{(-/-)}$; 7-9: PCSK9$^{(+/-)}$)

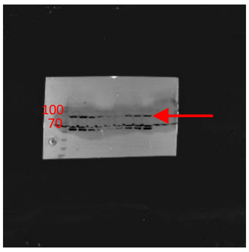

PCSK9

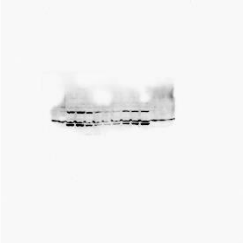

PCSK9

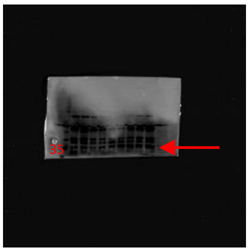

GAPDH

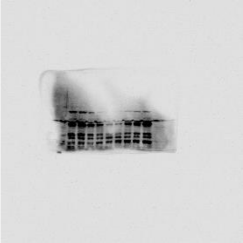

GAPDH

Repeated Figure 2P p-AP-1, AP-1 (From left to right, 1-3: PCSK9$^{(+/+)}$; 4-6: PCSK9$^{(-/-)}$; 7-9: PCSK9$^{(+/-)}$)

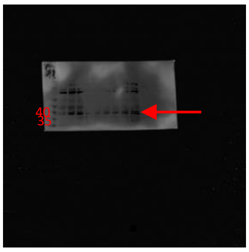

p-AP-1

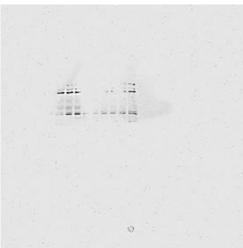

p-AP-1

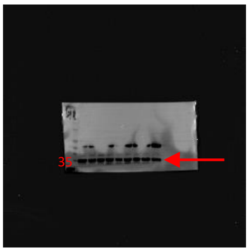

GAPDH

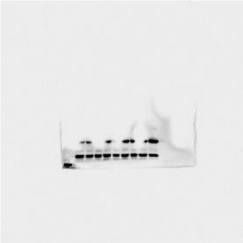

GAPDH

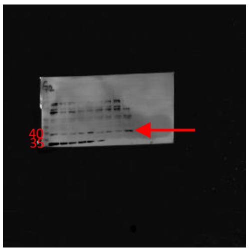

AP-1

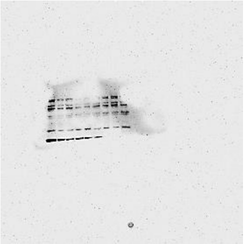

AP-1

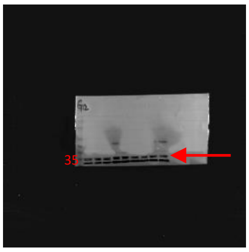

GAPDH

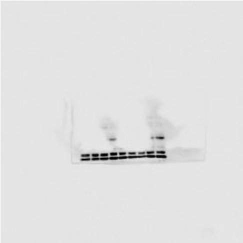

GAPDH

Repeated Figure 4A PCSK9 (From left to right, 1-3: Blank; 4-6: NC; 7-9: PCSK9 siRNA1;10-12: PCSK9 siRNA2)

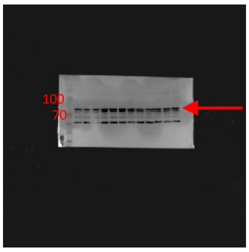

PCSK9

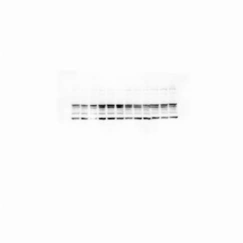

PCSK9

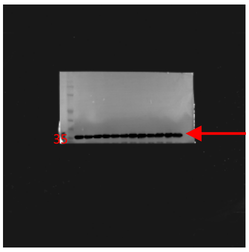

GAPDH

GAPDH

Repeated Figure 4I p-AP-1, AP-1 (From left to right, 1-3: Blank; 4-6: NC; 7-9: PCSK9 siRNA1;10-12: PCSK9 siRNA2)

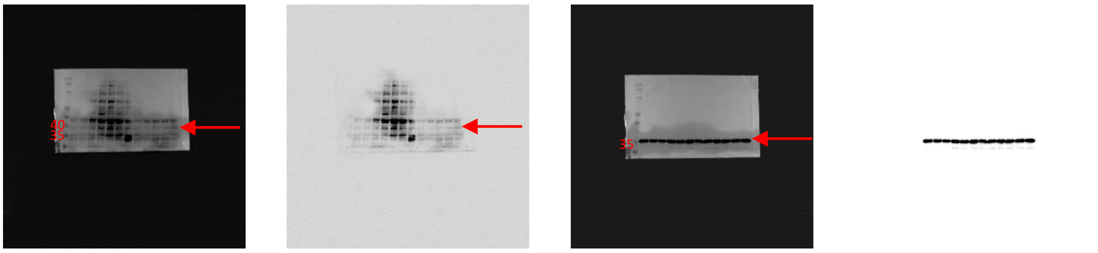

p-AP-1 p-AP-1 GAPDH GAPDH

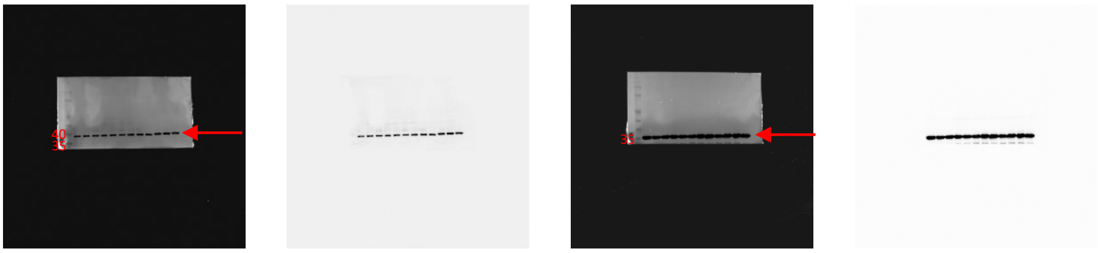

AP-1 AP-1 GAPDH GAPDH

Repeated Figure 4M p-PI3K, PI3K (From left to right, 1-2: BC; 3-4: Mod; 4-6: Pm; 7-8: P38i; 9-10: Pm+i)

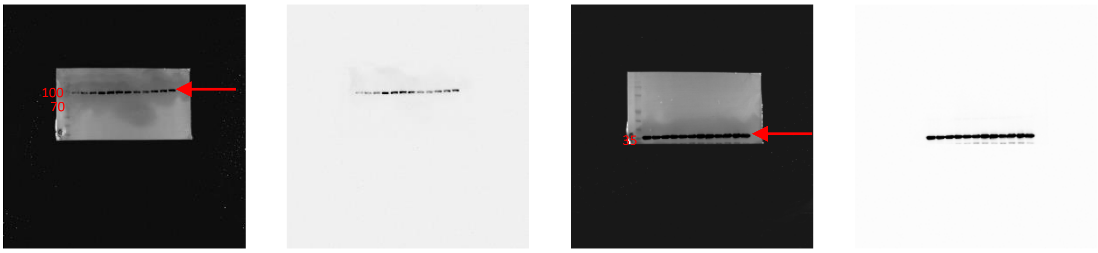

p-PI3K p-PI3K GAPDH GAPDH

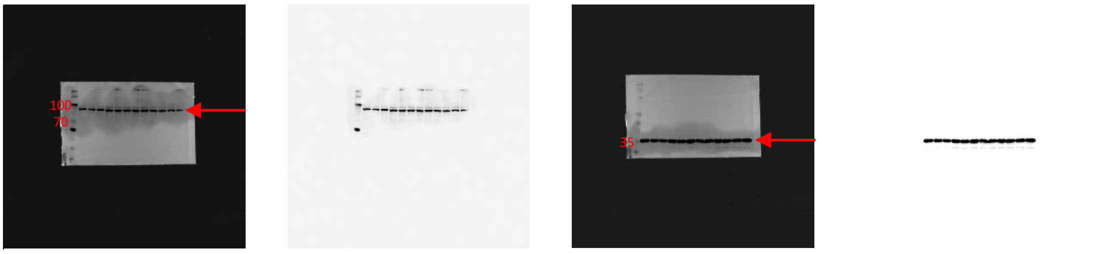

PI3K PI3K GAPDH GAPDH

Repeated Figure 4N p-AKT, AKT (From left to right, 1-2: BC; 3-4: Mod; 4-6: Pm; 7-8: P38i; 9-10: Pm+i)

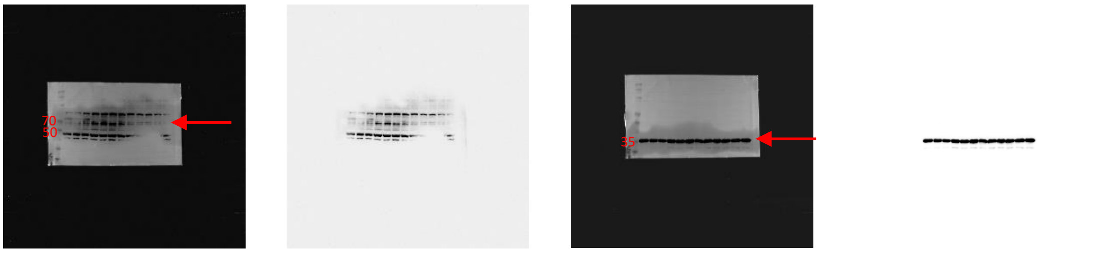

p-AKT p-AKT GAPDH GAPDH

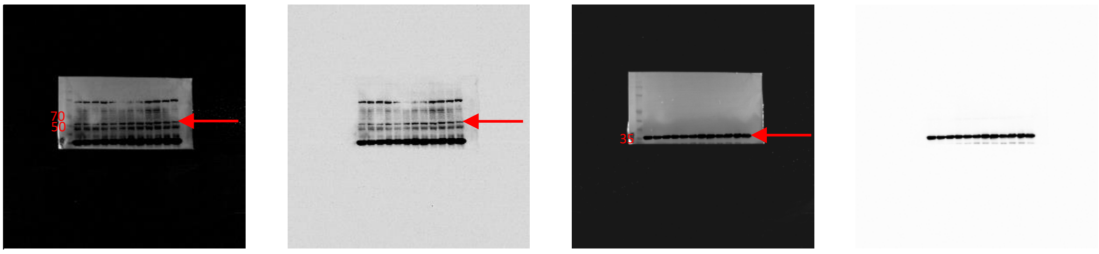

AKT AKT GAPDH GAPDH

Repeated Figure 4O p-MTOR, MTOR (From left to right, 1-2: BC; 3-4: Mod; 4-6: Pm; 7-8: P38i; 9-10: Pm+i)

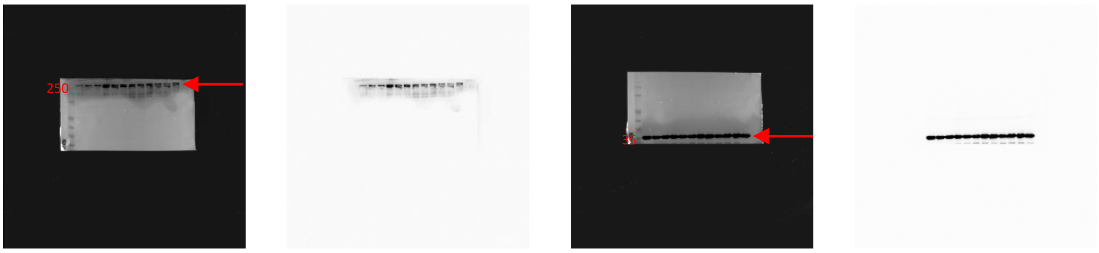

p-MTOR p-MTOR GAPDH GAPDH

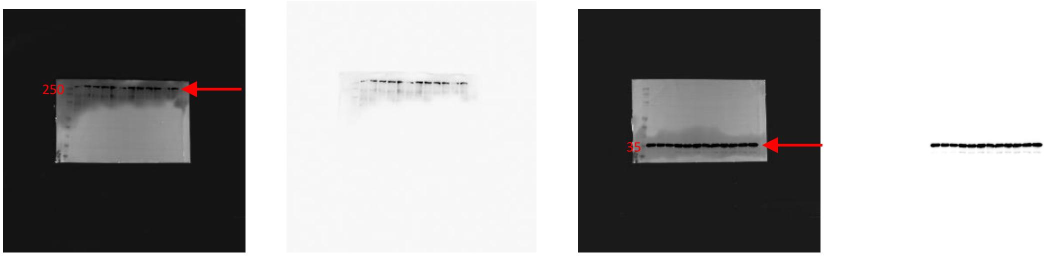

MTOR MTOR GAPDH GAPDH

Repeated Figure 5B LDLR (From left to right, 1-2: BC; 3-4: Mod; 4-6: Pm; 7-8: P38i; 9-10: Pm+i)

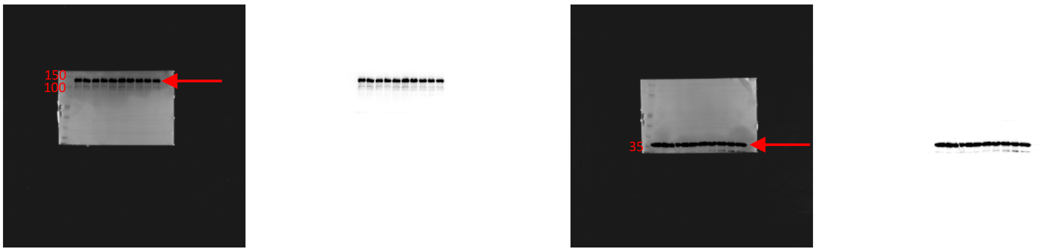

LDLR LDLR GAPDH GAPDH

Repeated Figure 5M p-JNK, JNK (From left to right, 1-2: BC; 3-4: Mod; 4-6: Pm; 7-8: P38i; 9-10: Pm+i)

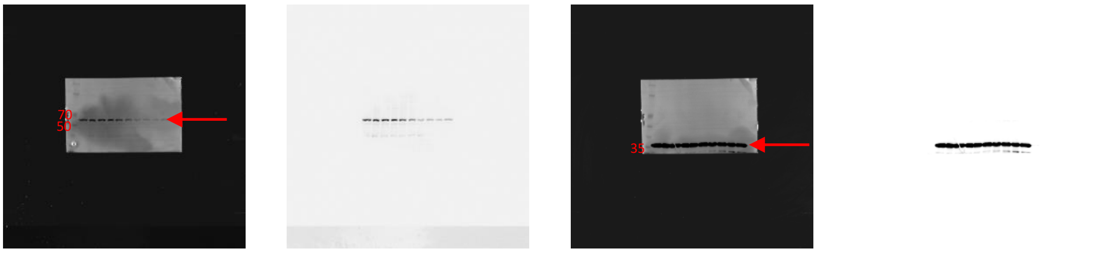

p-JNK p-JNK GAPDH GAPDH

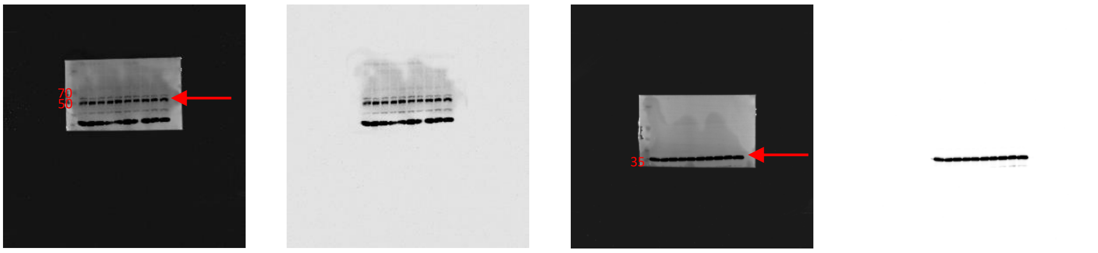

JNK JNK GAPDH GAPDH

Repeated Figure 5N p-PI3K, PI3K (From left to right, 1-2: BC; 3-4: Mod; 4-6: Pm; 7-8: P38i; 9-10: Pm+i)

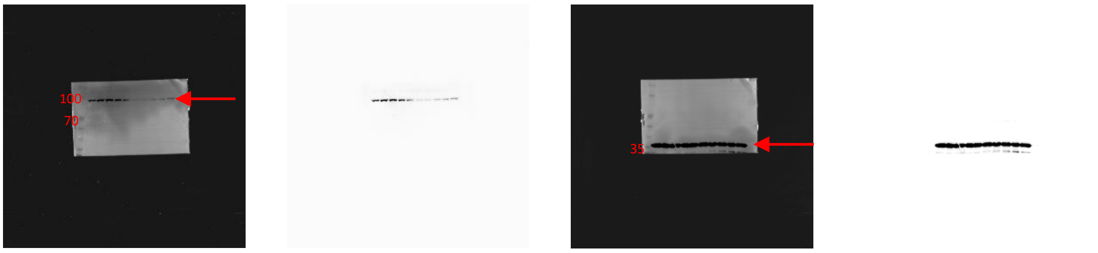

p-PI3K p-PI3K GAPDH GAPDH

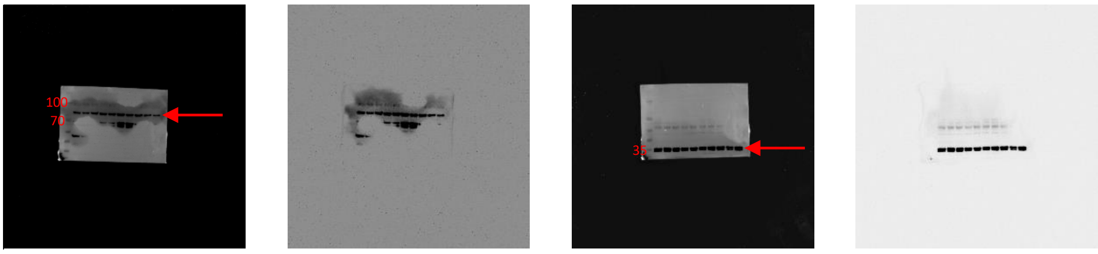

PI3K PI3K GAPDH GAPDH

Repeated Figure 5O p-AKT, AKT (From left to right, 1-2: BC; 3-4: Mod; 4-6: Pm; 7-8: P38i; 9-10: Pm+i)

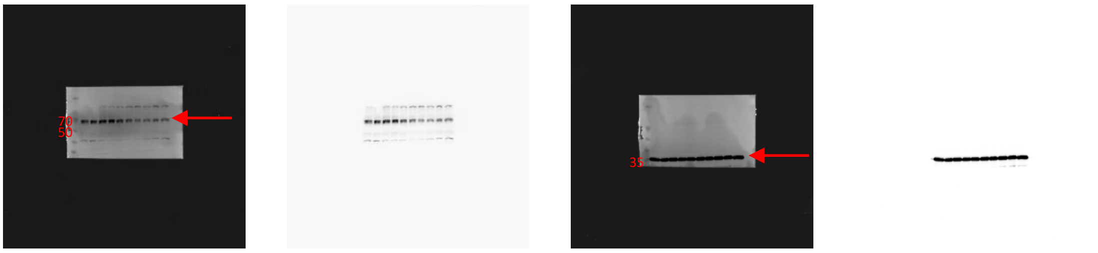

p-AKT p-AKT GAPDH GAPDH

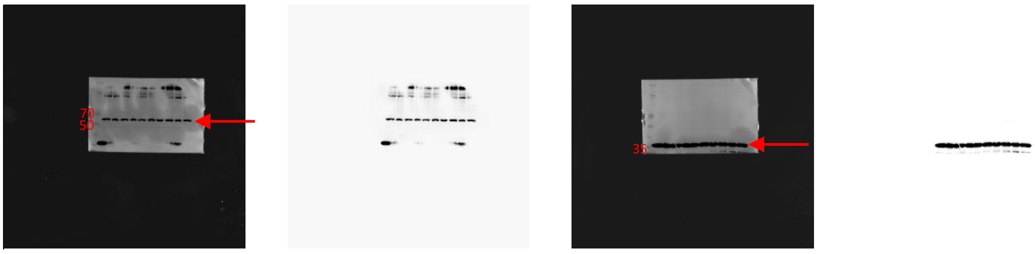

AKT AKT GAPDH GAPDH

Supplemented Figure 2C PCSK9 (From left to right, 1-3: normal chow; 4-6: high-fat)

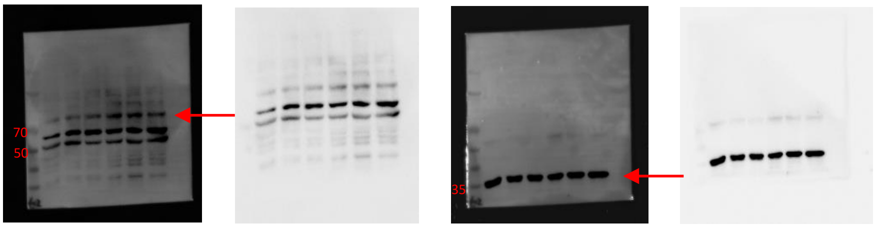

PCSK9 PCSK9 GAPDH GAPDH

Supplemented Figure 2D TNFα (From left to right, 1-3: normal chow; 4-6: high-fat)

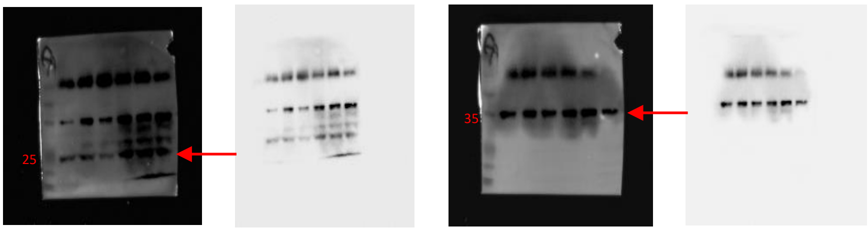

TNFα TNFα GAPDH GAPDH

Supplemented Figure 2E IL-1β (From left to right, 1-3: normal chow; 4-6: high-fat)

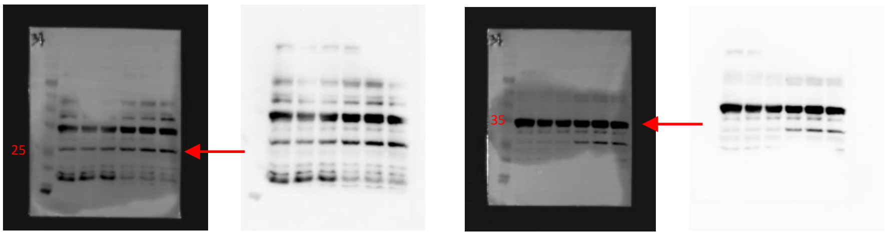

IL-1β IL-1β GAPDH GAPDH

Supplemented Figure 2F p-P65, P65 (From left to right, 1-3: normal chow; 4-6: high-fat)

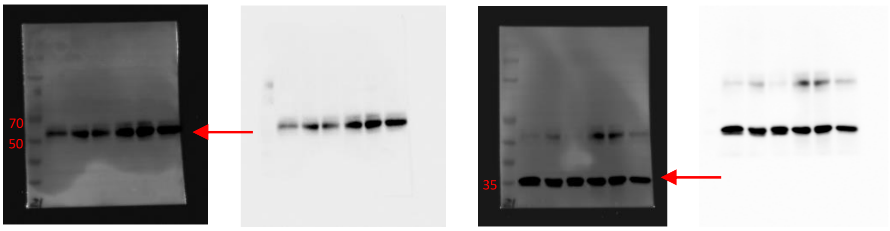

p-P65 p-P65 GAPDH GAPDH

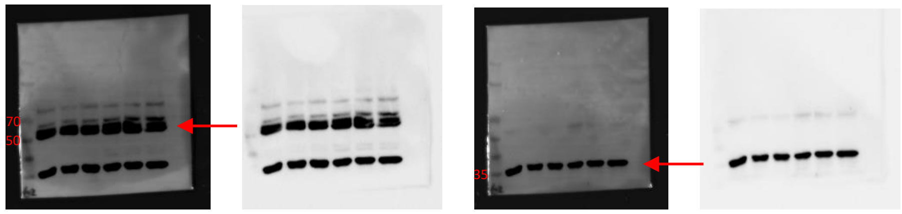

P65 P65 GAPDH GAPDH

Supplemented Figure 2G p-AP-1, AP-1 (From left to right, 1-3: normal chow; 4-6: high-fat)

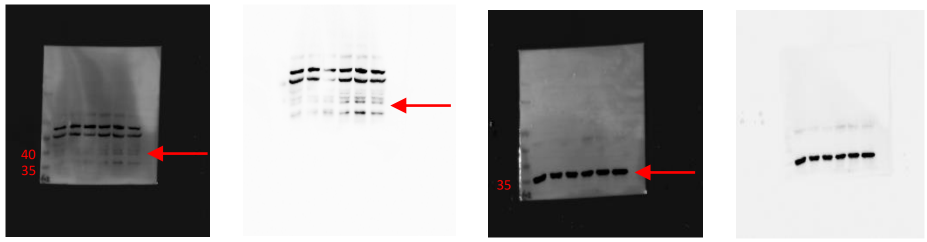

p-AP-1 p-AP-1 GAPDH GAPDH

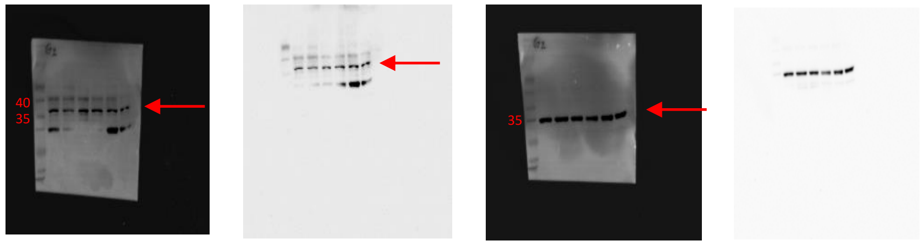

AP-1 AP-1 GAPDH GAPDH

Supplemented Figure 2H TLR2 (From left to right, 1-3: normal chow; 4-6: high-fat)

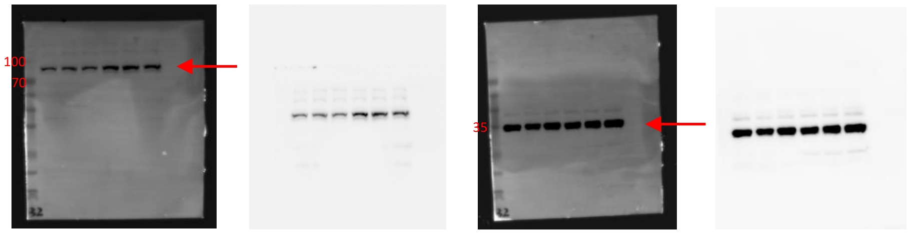

TLR2 TLR2 GAPDH GAPDH

Supplemented Figure 2I TLR4 (From left to right, 1-3: normal chow; 4-6: high-fat)

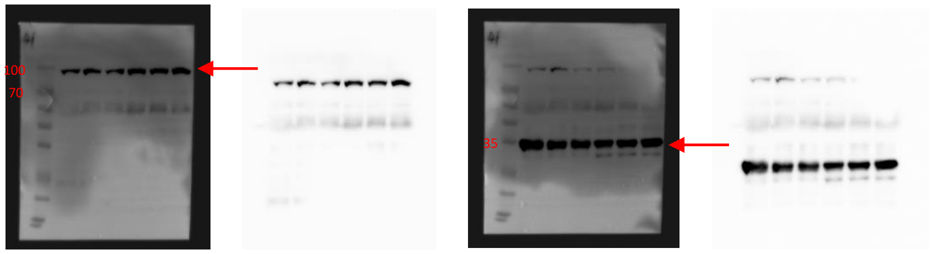

TLR4 TLR4 GAPDH GAPDH

Supplemented Figure 2J p-MyD88, MyD88 (From left to right, 1-3: normal chow; 4-6: high-fat)

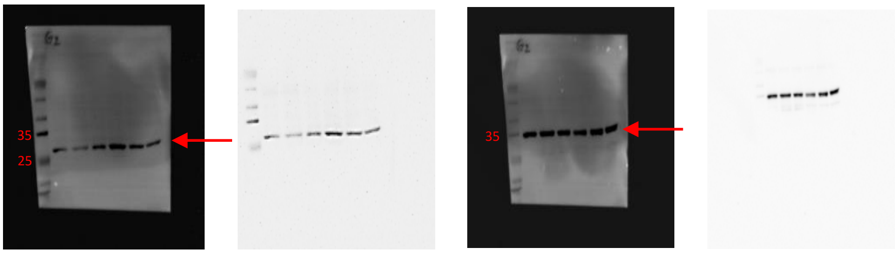

p-MYD88 p-MYD88 GAPDH GAPDH

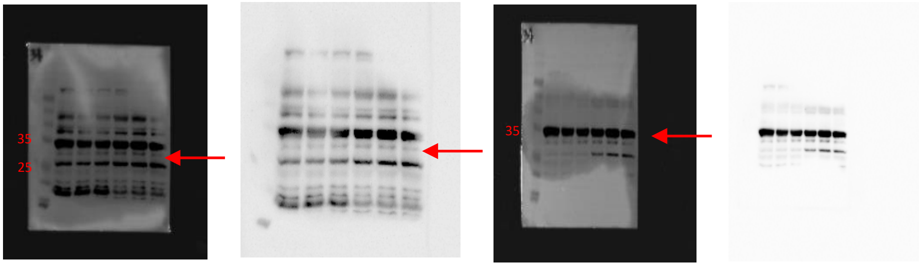

MYD88 MYD88 GAPDH GAPDH

Supplemented Figure 2K p-PI3K, PI3K (From left to right, 1-3: normal chow; 4-6: high-fat)

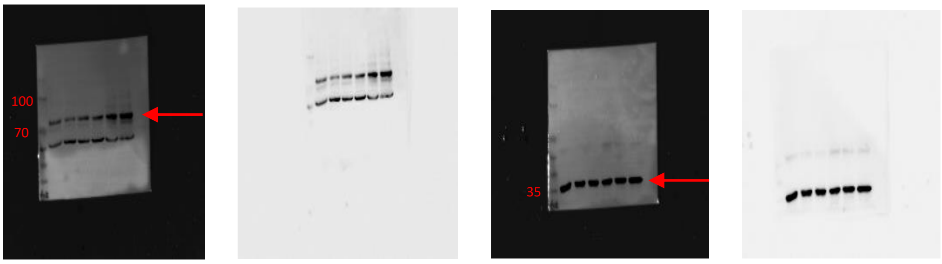

p-PI3K p-PI3K GAPDH GAPDH

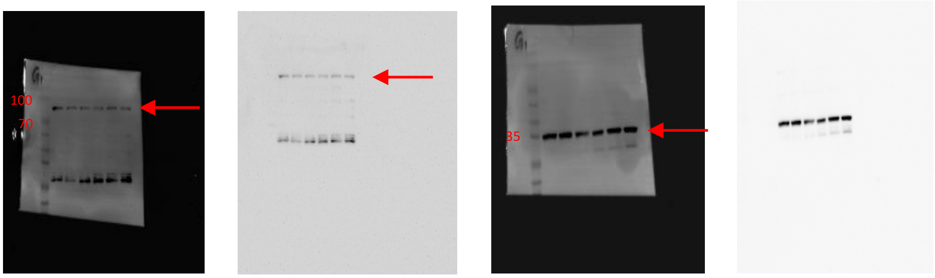

PI3K PI3K GAPDH GAPDH

Supplemented Figure 2L p-AKT, AKT (From left to right, 1-3: normal chow; 4-6: high-fat)

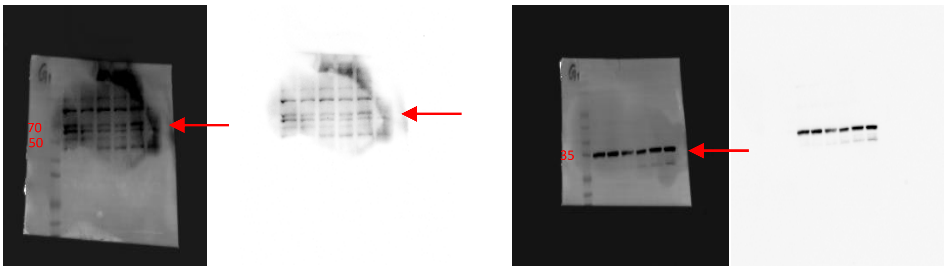

p-AKT p-AKT GAPDH GAPDH

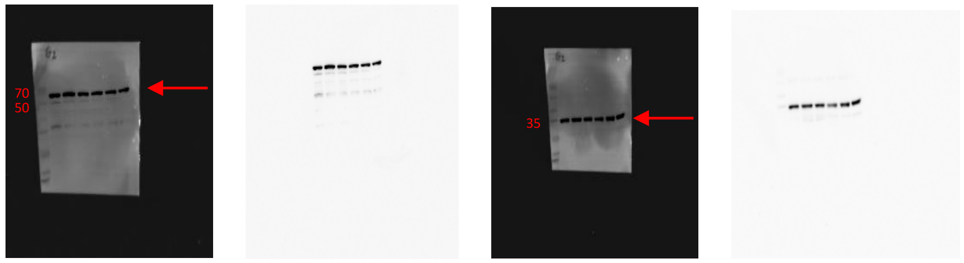

AKT AKT GAPDH GAPDH

Supplemented Figure 2M p-mTOR, mTOR (From left to right, 1-3: normal chow; 4-6: high-fat)

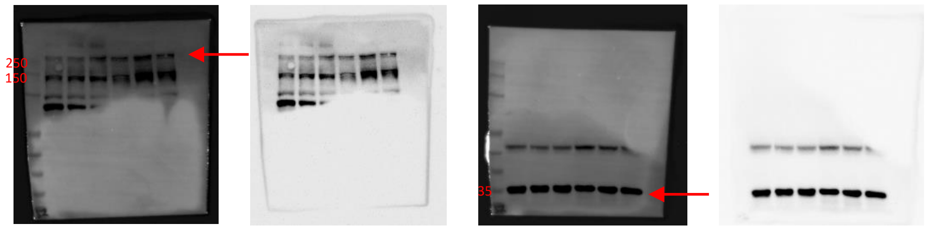

p-mTOR p-mTOR GAPDH GAPDH

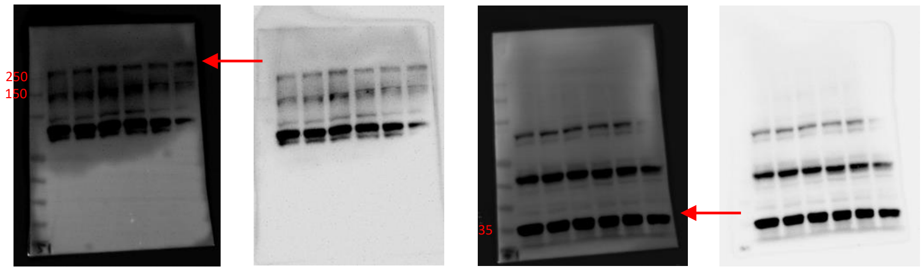

mTOR mTOR GAPDH GAPDH

Supplemented Figure 2N p-P38, P38 (From left to right, 1-3: normal chow; 4-6: high-fat)

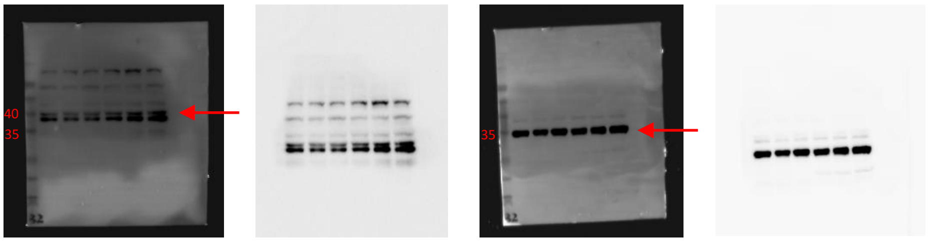

p-P38 p-P38 GAPDH GAPDH

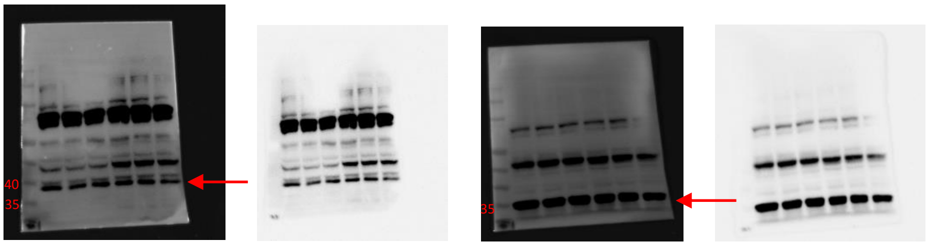

P38 P38 GAPDH GAPDH

Supplemented Figure 2O p-ERK, ERK (From left to right, 1-3: normal chow; 4-6: high-fat)

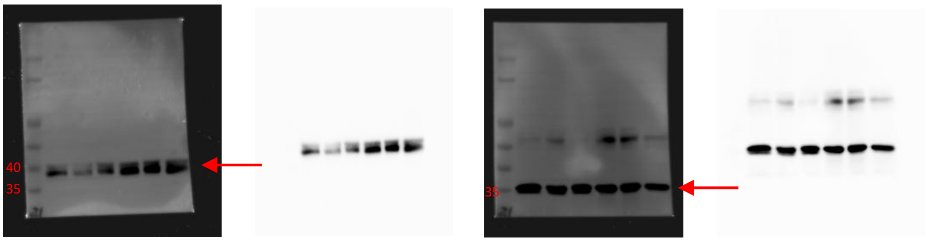

p-ERK p-ERK GAPDH GAPDH

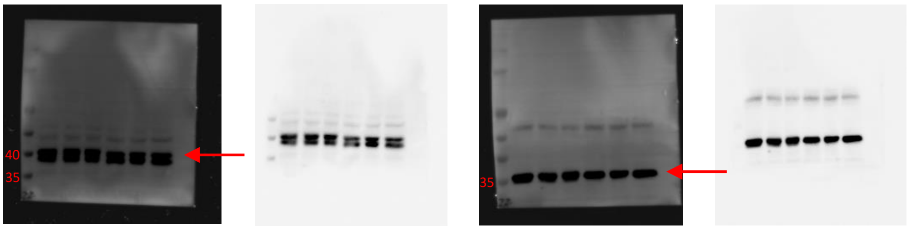

ERK ERK GAPDH GAPDH

Supplemented Figure 2P p-JNK, JNK (From left to right, 1-3: normal chow; 4-6: high-fat)

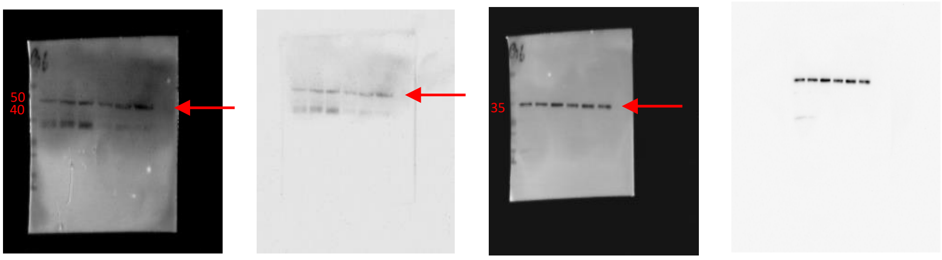

p-JNK p-JNK GAPDH GAPDH

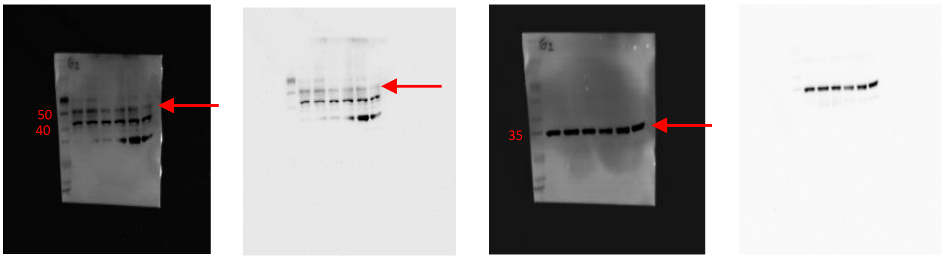

JNK JNK GAPDH GAPDH

Supplemented Figure 3C PCSK9 (From left to right, 1-3: Blank; 4-6: Model; 7-9: NC)

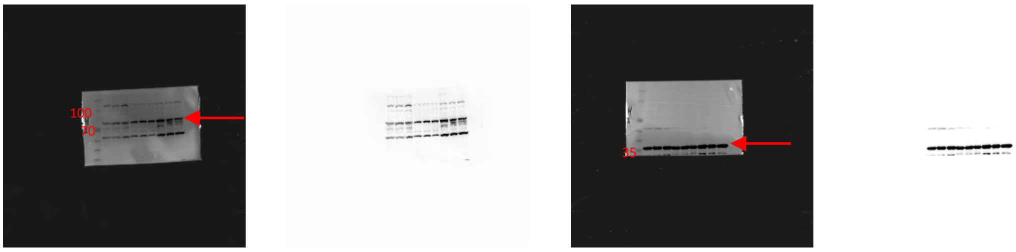

PCSK9 PCSK9 GAPDH GAPDH

Supplemented Figure 3D TNFα (From left to right, 1-3: Blank; 4-6: Model; 7-9: NC)

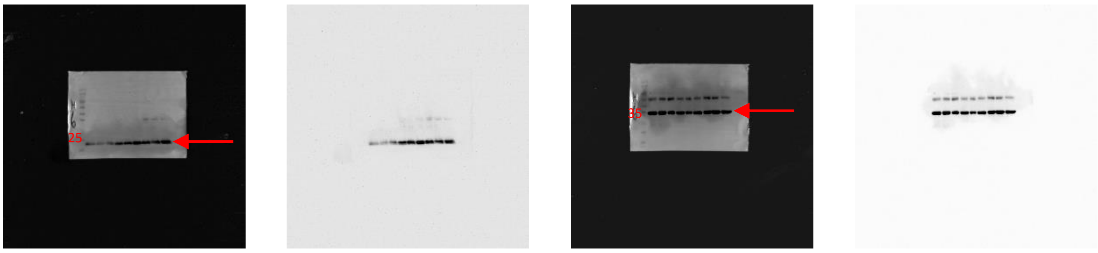

TNFα TNFα GAPDH GAPDH

Supplemented Figure 3E IL-6 (From left to right, 1-3: Blank; 4-6: Model; 7-9: NC)

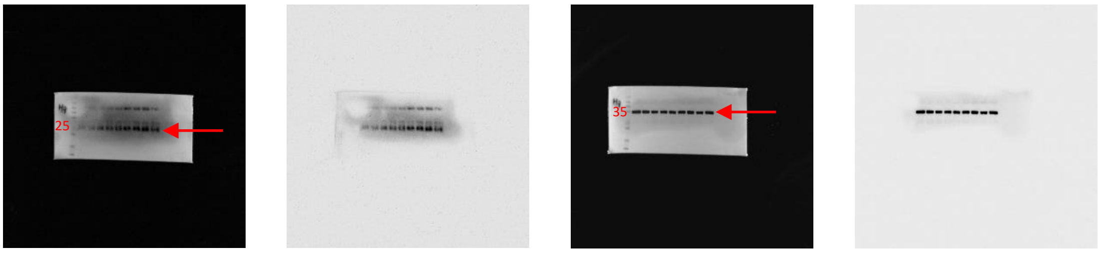

IL-6 IL-6 GAPDH GAPDH

Supplemented Figure 3F IL-1β (From left to right, 1-3: Blank; 4-6: Model; 7-9: NC)

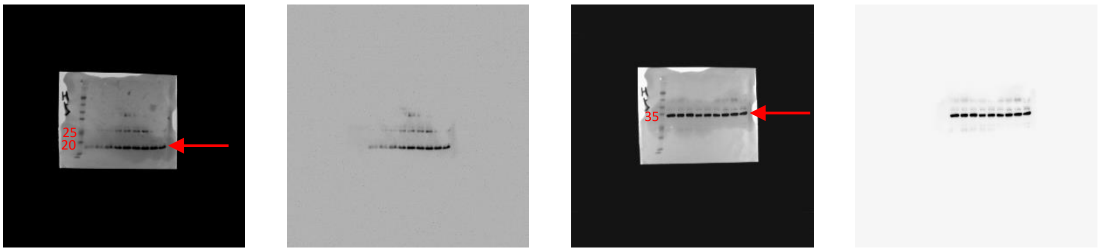

IL-1β IL-1β GAPDH GAPDH

Supplemented Figure 3G TLR2 (From left to right, 1-3: Blank; 4-6: Model; 7-9: NC)

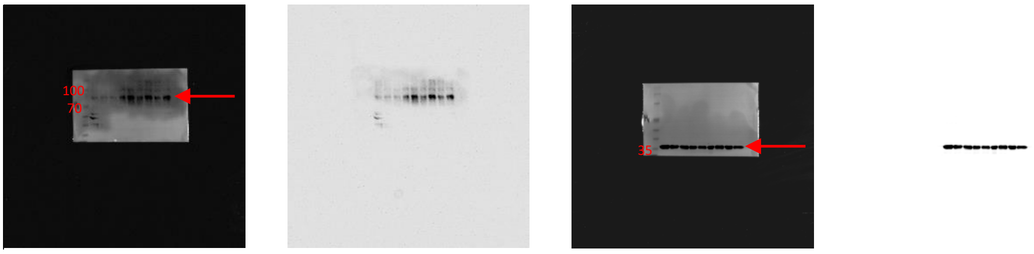

TLR2 TLR2 GAPDH GAPDH

Supplemented Figure 3H TLR4 (From left to right, 1-3: Blank; 4-6: Model; 7-9: NC)

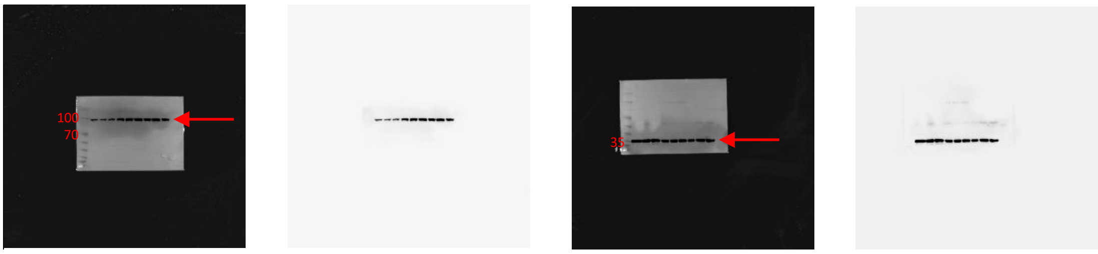

TLR4 TLR4 GAPDH GAPDH

Supplemented Figure 3I p-MyD88, MyD88 (From left to right, 1-3: Blank; 4-6: Model; 7-9: NC)

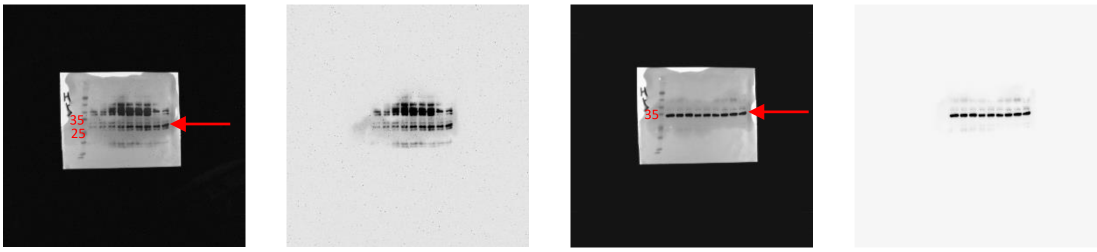

p-MyD88 p-MyD88 GAPDH GAPDH

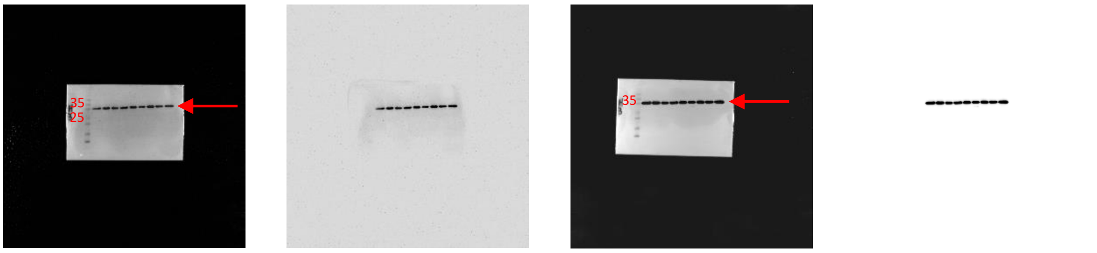

MyD88 MyD88 GAPDH GAPDH

Supplemented Figure 3J p-P65, P65 (From left to right, 1-3: Blank; 4-6: Model; 7-9: NC)

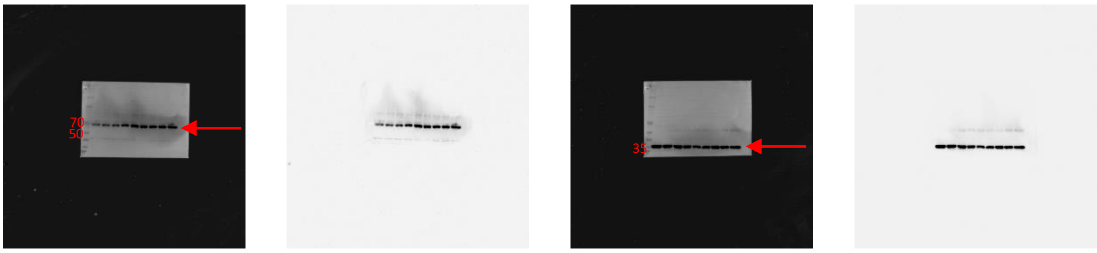

p-P65 p-P65 GAPDH GAPDH

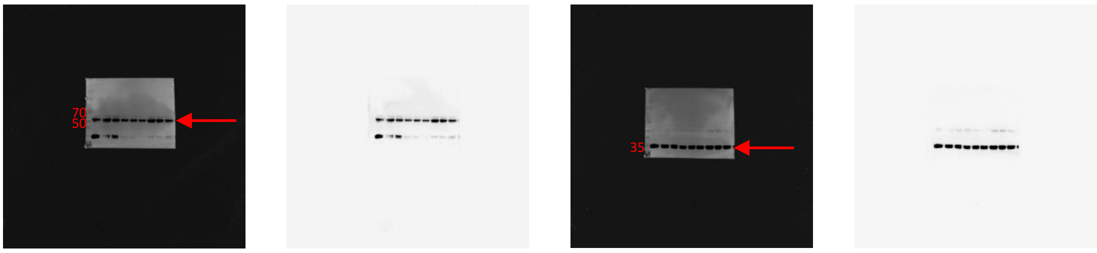

P65 P65 GAPDH GAPDH

Supplemented Figure 3K p-P38, P38 (From left to right, 1-3: Blank; 4-6: Model; 7-9: NC)

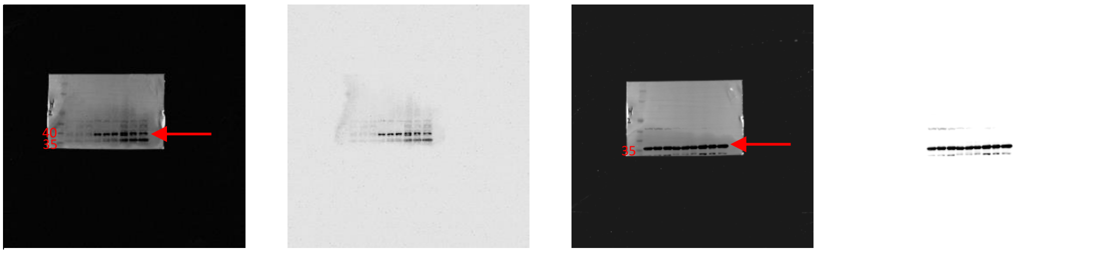

p-P38 p-P38 GAPDH GAPDH

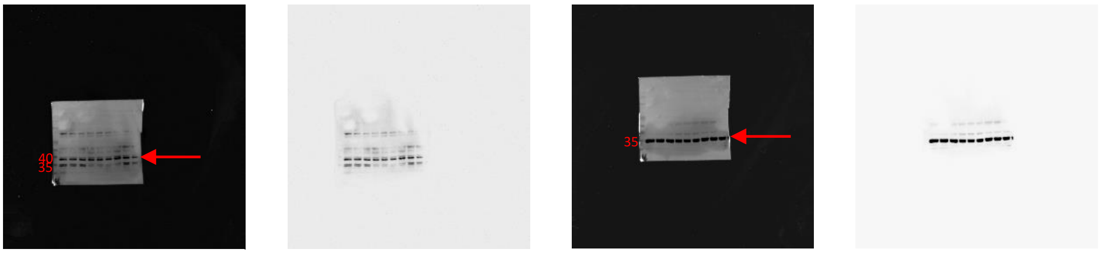

P38 P38 GAPDH GAPDH

Supplemented Figure 3L p-ERK, ERK (From left to right, 1-3: Blank; 4-6: Model; 7-9: NC)

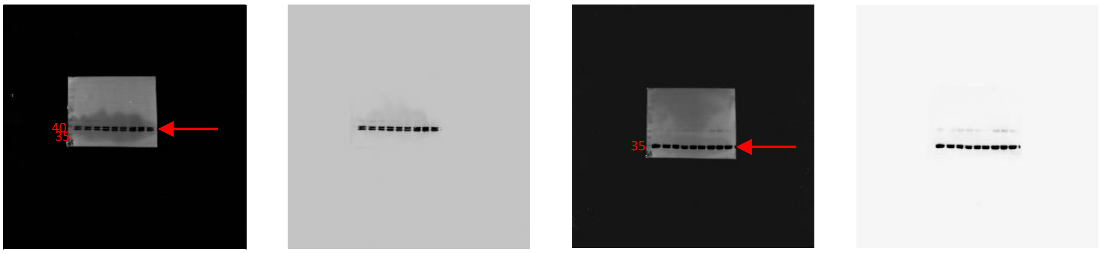

p-ERK p-ERK GAPDH GAPDH

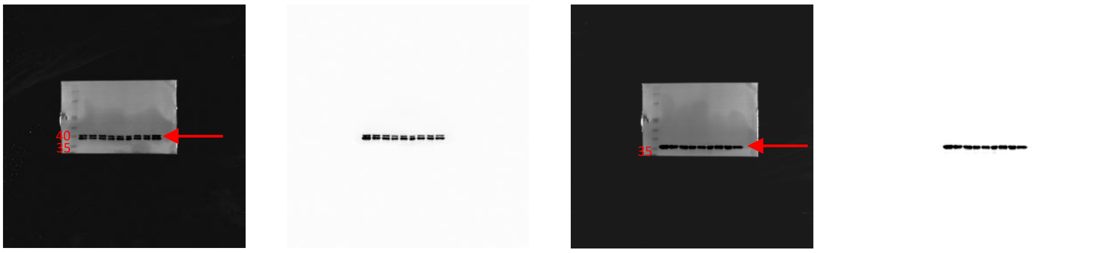

ERK ERK GAPDH GAPDH

Supplemented Figure 3M p-JNK, JNK (From left to right, 1-3: Blank; 4-6: Model; 7-9: NC)

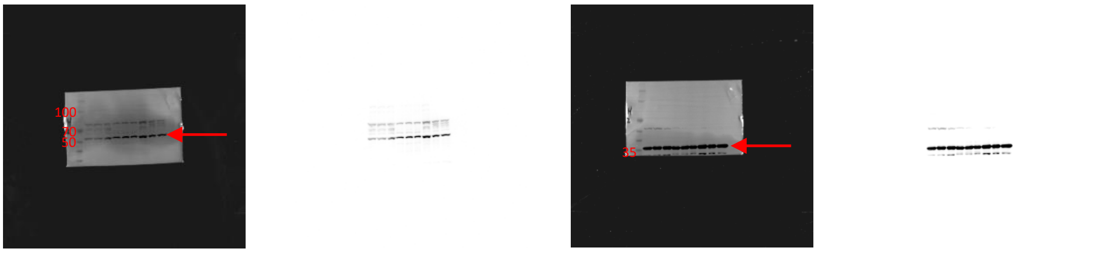

p-JNK p-JNK GAPDH GAPDH

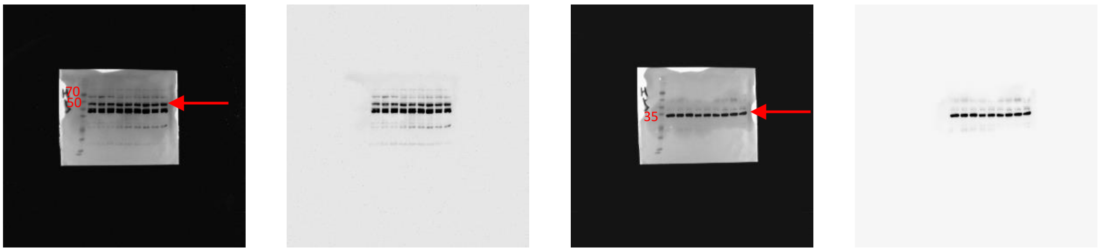

JNK JNK GAPDH GAPDH

Supplemented Figure 3N p-PI3K, PI3K (From left to right, 1-3: Blank; 4-6: Model; 7-9: NC)

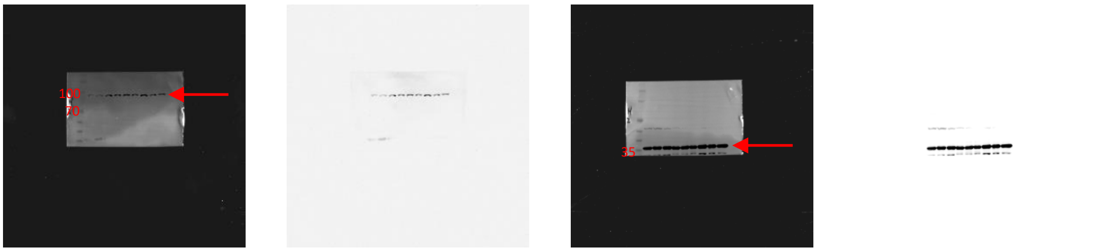

p-PI3K p-PI3K GAPDH GAPDH

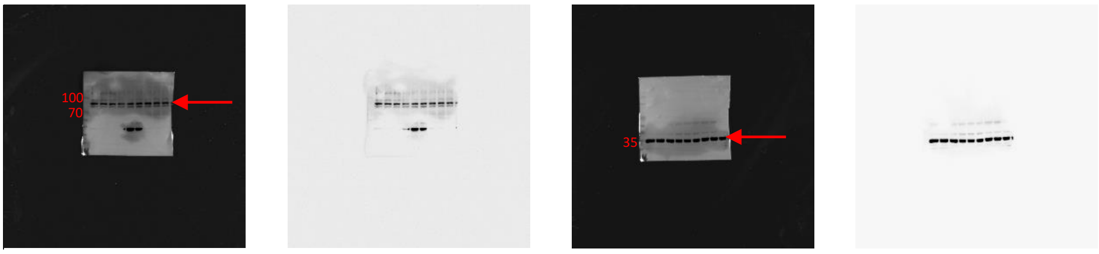

PI3K PI3K GAPDH GAPDH

Supplemented Figure 3O p-AKT, AKT (From left to right, 1-3: Blank; 4-6: Model; 7-9: NC)

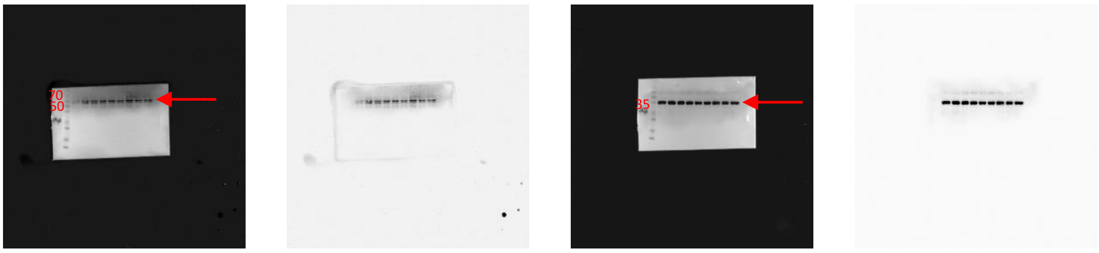

p-AKT p-AKT GAPDH GAPDH

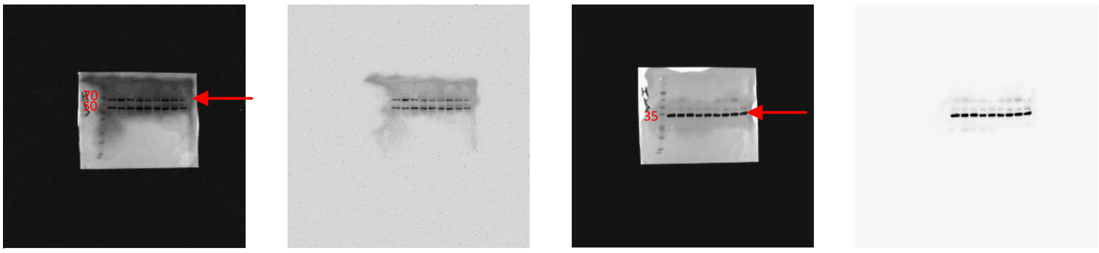

AKT AKT GAPDH GAPDH

Supplemented Figure 3P p-MTOR, MTOR (From left to right, 1-3: Blank; 4-6: Model; 7-9: NC)

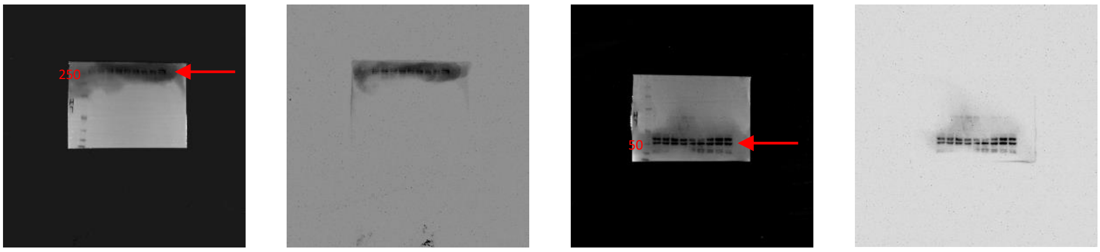

p-MTOR p-MTOR β-tublin β-tublin

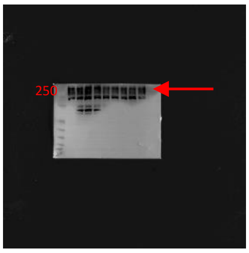

MTOR

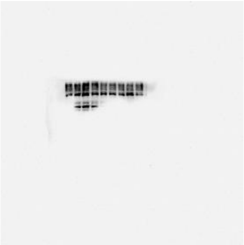

MTOR

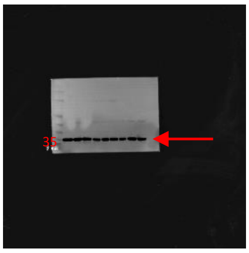

GAPDH

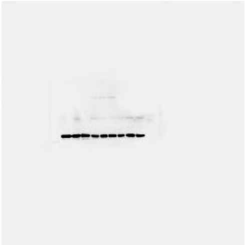

GAPDH
