# Supplementary material for: Conditional knockdown of hepatic PCSK9 ameliorates high-fat diet-induced liver inflammation in mice
Source: Front Pharmacol. 2025 Feb 3;16:1528250. doi: 10.3389/fphar.2025.1528250 (PMC11830812; doi:10.3389/fphar.2025.1528250)

H&E staining of hepatic PCSK9$^{liver(+/+)}$ mice

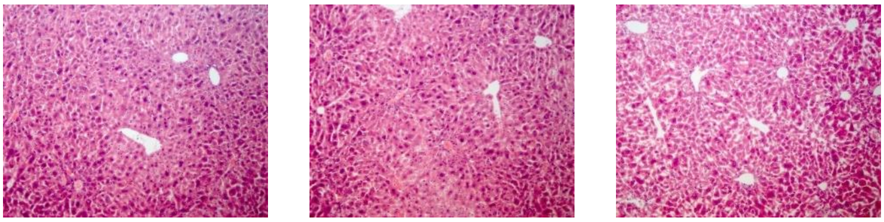

Oil Red O staining of hepatic PCSK9$^{liver(+/+)}$ mice

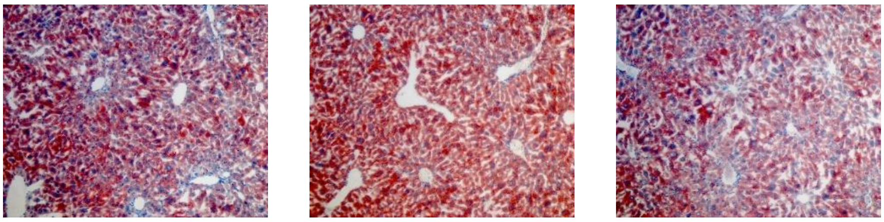

H&E staining of hepatic PCSK9$^{liver(-/-)}$ mice

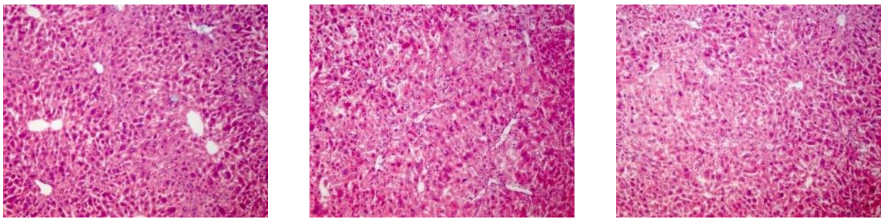

Oil Red O staining of hepatic PCSK9$^{liver(-/-)}$ mice

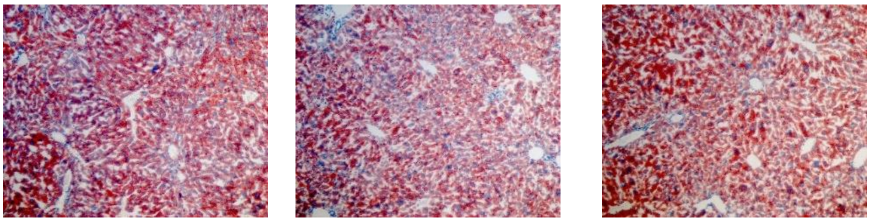

H&E staining of hepatic PCSK9$^{liver(+/-)}$ mice

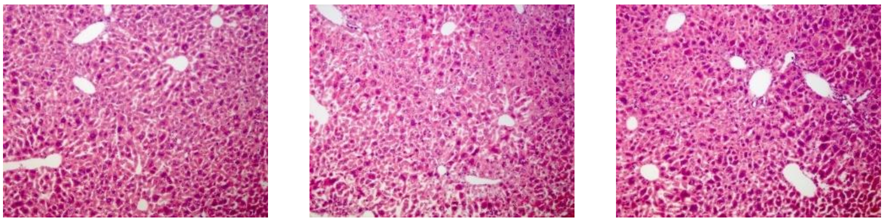

Oil Red O staining of hepatic PCSK9$^{liver(+/-)}$ mice

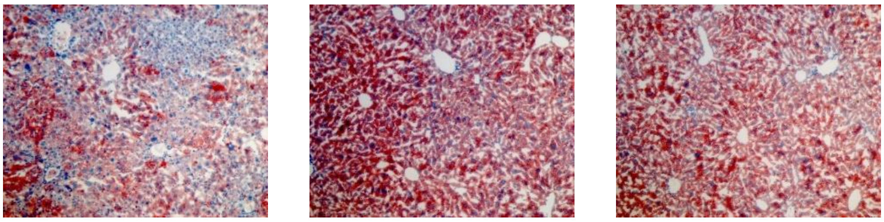

Supplement: Supplementary file 1 [file Presentation1.zip › Data Sheet 1/Supplementary material S3.pdf]
