# Supplementary material for: Conditional knockdown of hepatic PCSK9 ameliorates high-fat diet-induced liver inflammation in mice
Source: Front Pharmacol. 2025 Feb 3;16:1528250. doi: 10.3389/fphar.2025.1528250 (PMC11830812; doi:10.3389/fphar.2025.1528250)
Supplement: Supplementary file 1 [file Presentation1.zip › Data Sheet 1/Supplementary material WB RAW DATA.pdf]

Western blot RAW data

Figure 1C PCSK9 (From left to right, 1-3: PCSK9$^{(+/+)}$; 4-6: PCSK9$^{(-/-)}$; 7-9: PCSK9$^{(+/-)}$)

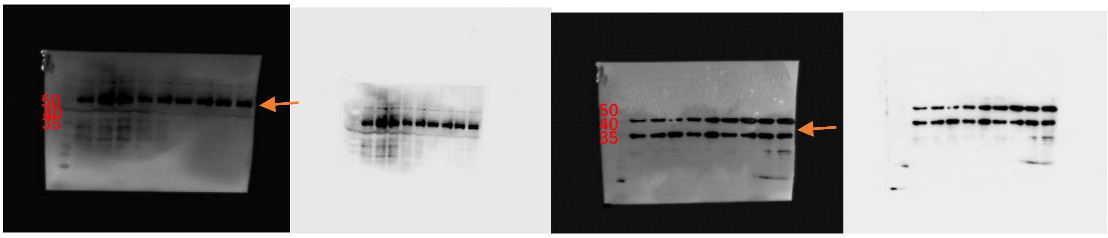

PCSK9 PCSK9 GAPDH GAPDH

Figure 1D LDLR (From left to right, 1-3: PCSK9$^{(+/+)}$; 4-6: PCSK9$^{(-/-)}$; 7-9: PCSK9$^{(+/-)}$)

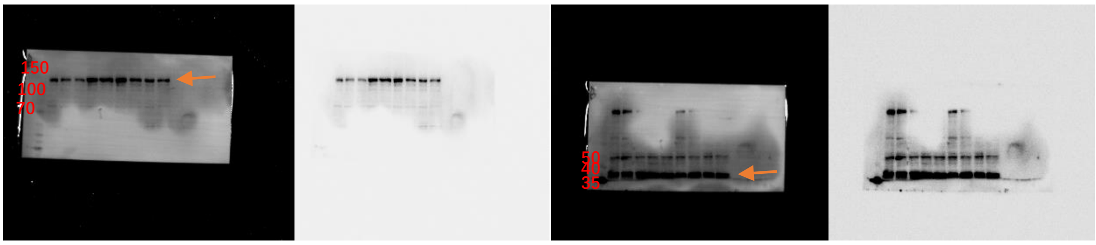

LDLR LDLR GAPDH GAPDH

Figure 2E IL-6 (From left to right, 1-3: PCSK9$^{(+/+)}$; 4-6: PCSK9$^{(-/-)}$; 7-9: PCSK9$^{(+/-)}$)

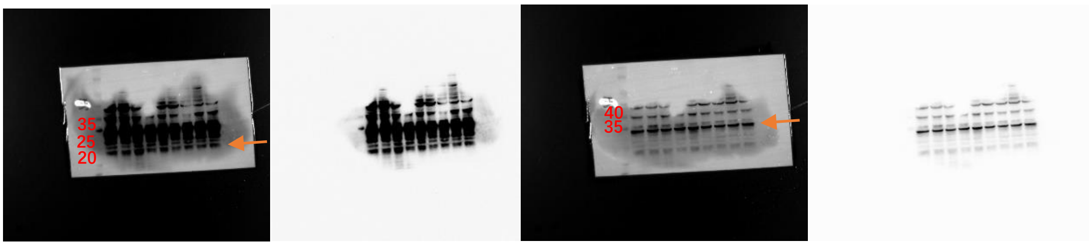

IL-6 IL-6 GAPDH GAPDH

Figure 2F IL-1β (From left to right, 1-3: PCSK9$^{(+/+)}$; 4-6: PCSK9$^{(-/-)}$; 7-9: PCSK9$^{(+/-)}$)

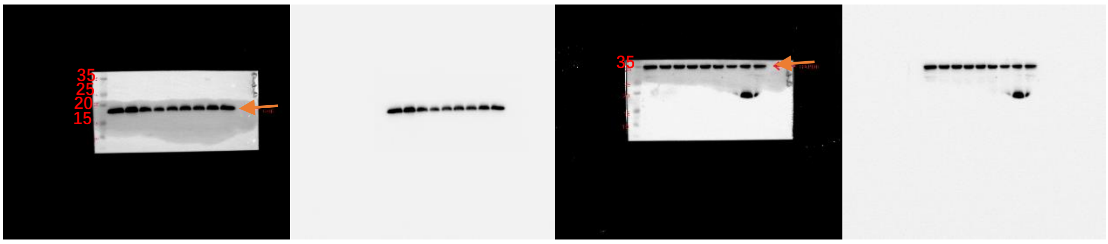

IL-1β IL-1β GAPDH GAPDH

Figure 2G TNFα (From left to right, 1-3: PCSK9$^{(+/+)}$; 4-6: PCSK9$^{(-/-)}$; 7-9: PCSK9$^{(+/-)}$)

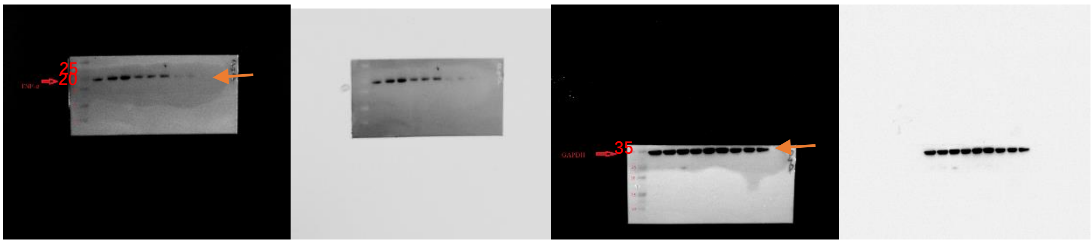

TNFα TNFα GAPDH GAPDH

Figure 2H TLR4 (From left to right, 1-3: PCSK9$^{(+/+)}$; 4-6: PCSK9$^{(-/-)}$; 7-9: PCSK9$^{(+/-)}$)

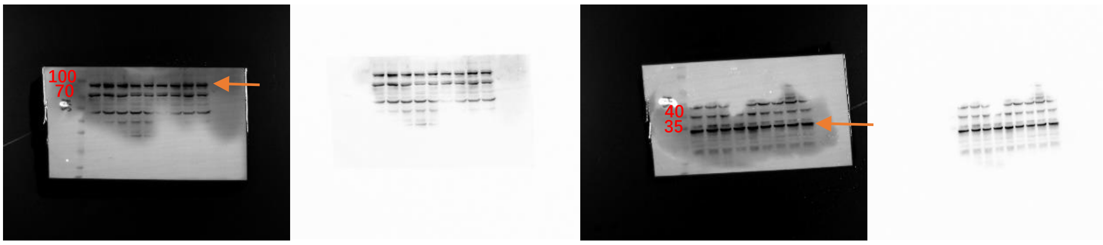

TLR4 TLR4 GAPDH GAPDH

Figure 2M TLR2 (From left to right, 1-3: PCSK9$^{(+/+)}$; 4-6: PCSK9$^{(-/-)}$; 7-9: PCSK9$^{(+/-)}$)

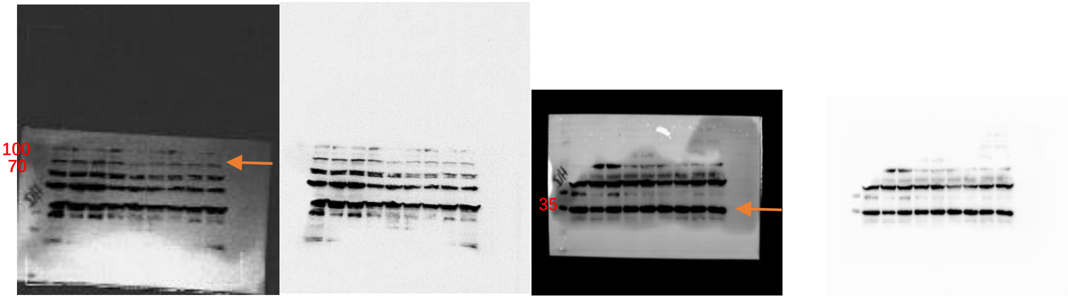

TLR2 TLR2 GAPDH GAPDH

Figure 2N p-MyD88 (1-3: PCSK9$^{(+/+)}$; 4-6: PCSK9$^{(-/-)}$; 7-9: PCSK9$^{(+/-)}$)

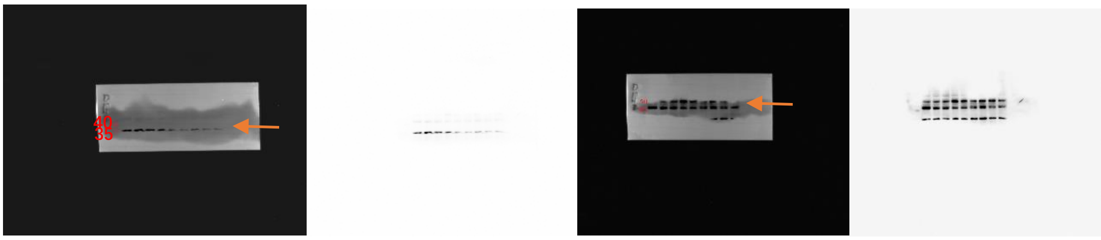

p-MyD88 p-MyD88 β-actin β-actin

MyD88 (From left to right, 1-3: PCSK9$^{(+/+)}$; 4-6: PCSK9$^{(-/-)}$; 7-9: PCSK9$^{(+/-)}$)

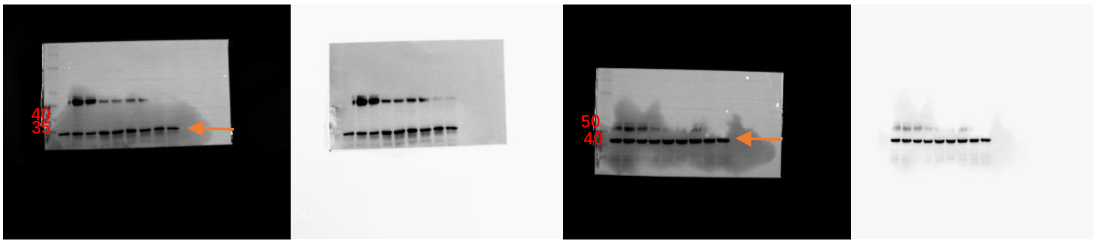

MyD88 MyD88 β-actin β-actin

Figure 2O p-NF-κB P65 (1-3: PCSK9$^{(+/+)}$; 4-6: PCSK9$^{(-/-)}$; 7-9: PCSK9$^{(+/-)}$)

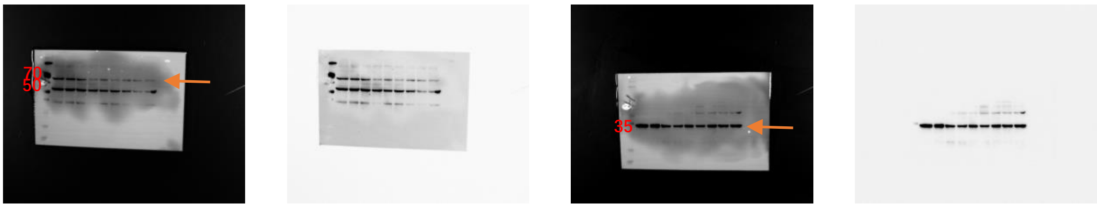

p-NF-κB P65 p-NF-κB P65 GAPDH GAPDH

NF-κB P65 (From left to right, 1-3: PCSK9$^{(+/+)}$; 4-6: PCSK9$^{(-/-)}$; 7-9: PCSK9$^{(+/-)}$)

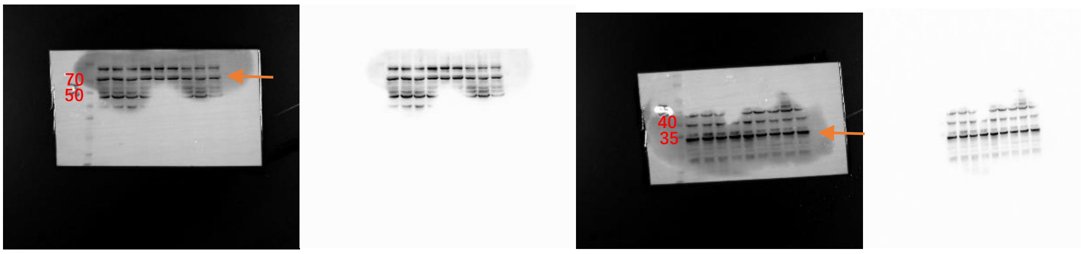

NF-κB P65 NF-κB P65 GAPDH GAPDH

Figure 2P p-AP-1 (From left to right, 1-3: PCSK9$^{(+/+)}$; 4-6: PCSK9$^{(-/-)}$; 7-9: PCSK9$^{(+/-)}$)

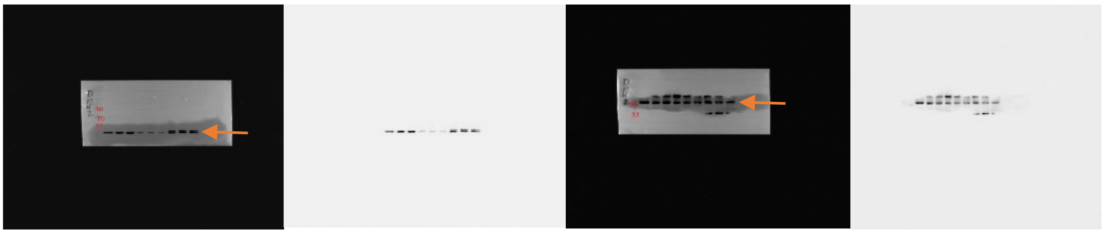

p-AP-1 p-AP-1 β-actin β-actin

AP-1 (From left to right, 1-3: PCSK9$^{(+/+)}$; 4-6: PCSK9$^{(-/-)}$; 7-9: PCSK9$^{(+/-)}$)

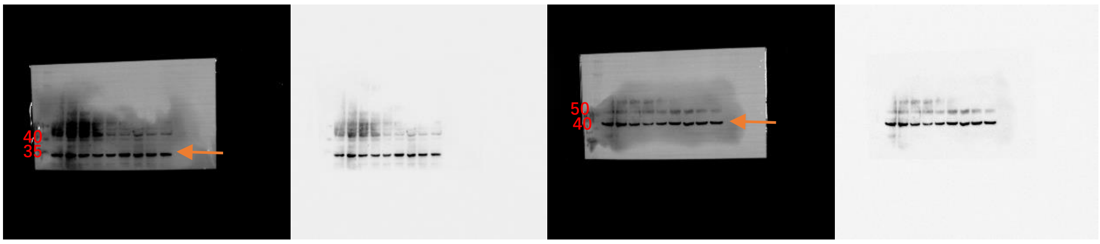

AP-1 AP-1 β-actin β-actin

Figure 3E p-P38 MAPK (From left to right, 1-3: PCSK9$^{(+/+)}$; 4-6: PCSK9$^{(-/-)}$; 7-9: PCSK9$^{(+/-)}$)

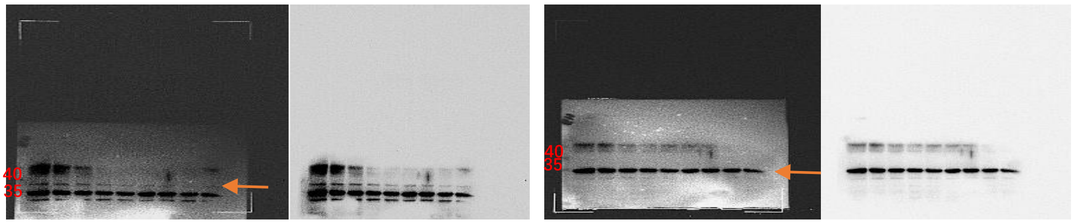

p-P38 MAPK p-P38 MAPK GAPDH GAPDH

P38 MAPK (From left to right, 1-3: PCSK9$^{(+/+)}$; 4-6: PCSK9$^{(-/-)}$; 7-9: PCSK9$^{(+/-)}$)

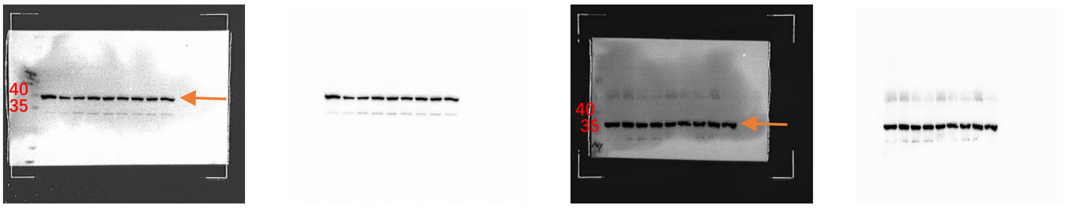

P38 MAPK P38 MAPK GAPDH GAPDH

Figure 3F p-ERK1/2 (From left to right, 1-3: PCSK9$^{(+/+)}$; 4-6: PCSK9$^{(-/-)}$; 7-9: PCSK9$^{(+/-)}$)

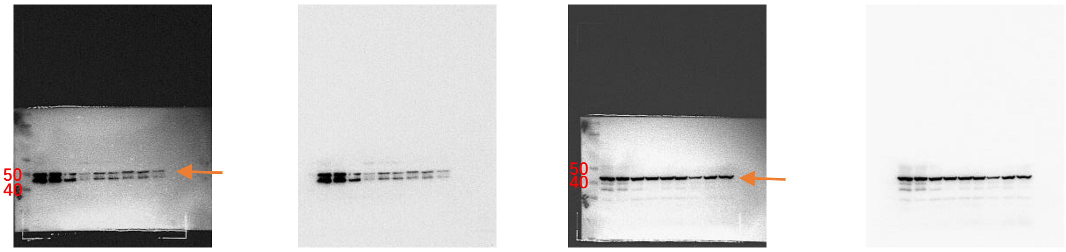

p-ERK1/2 p-ERK1/2 β-actin β-actin

ERK1/2 (From left to right, 1-3: PCSK9$^{(+/+)}$; 4-6: PCSK9$^{(-/-)}$; 7-9: PCSK9$^{(+/-)}$)

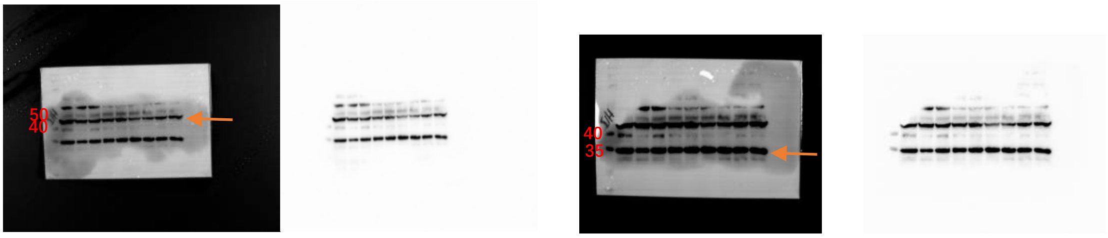

ERK1/2 ERK1/2 GAPDH GAPDH

Figure 3G p-JNK (From left to right, 1-3: PCSK9$^{(+/+)}$; 4-6: PCSK9$^{(-/-)}$; 7-9: PCSK9$^{(+/-)}$)

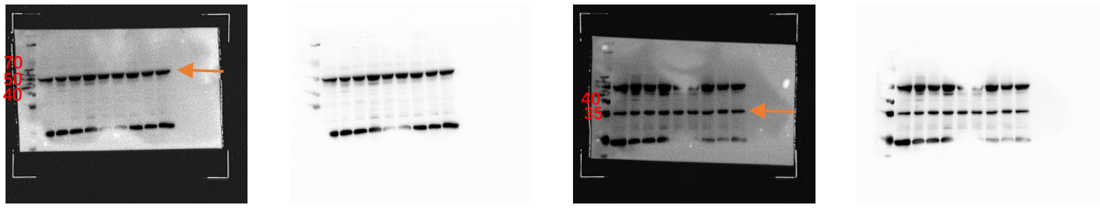

p-JNK p-JNK GAPDH GAPDH

JNK (From left to right, 1-3: PCSK9$^{(+/+)}$; 4-6: PCSK9$^{(-/-)}$; 7-9: PCSK9$^{(+/-)}$)

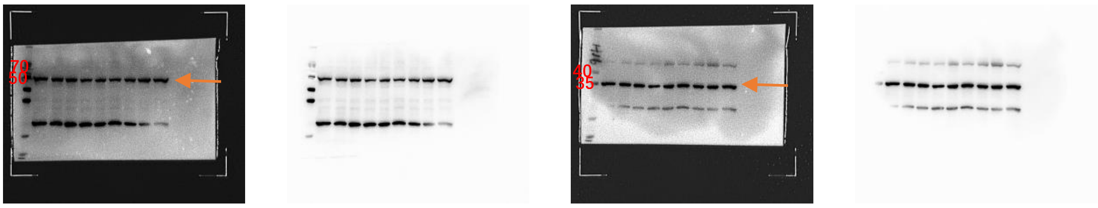

JNK JNK GAPDH GAPDH

Figure 3H p-PI3K (From left to right, 1-3: PCSK9$^{(+/+)}$; 4-6: PCSK9$^{(-/-)}$; 7-9: PCSK9$^{(+/-)}$)

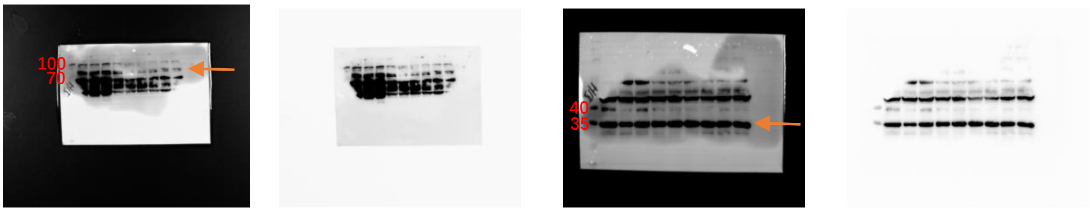

p-PI3K p-PI3K GAPDH GAPDH

PI3K (From left to right, 1-3: PCSK9$^{(+/+)}$; 4-6: PCSK9$^{(-/-)}$; 7-9: PCSK9$^{(+/-)}$)

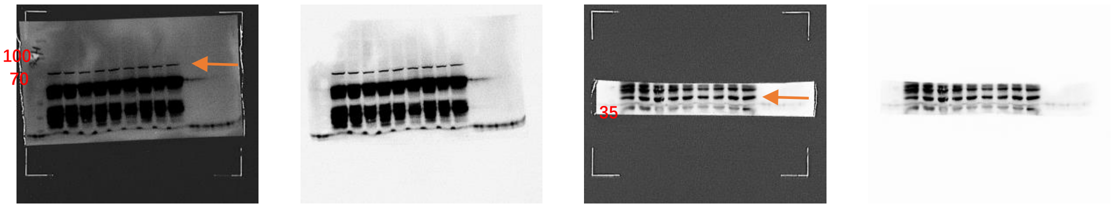

PI3K PI3K GAPDH GAPDH

Figure 3J p-AKT (From left to right, 1-3: PCSK9$^{(+/+)}$; 4-6: PCSK9$^{(-/-)}$; 7-9: PCSK9$^{(+/-)}$)

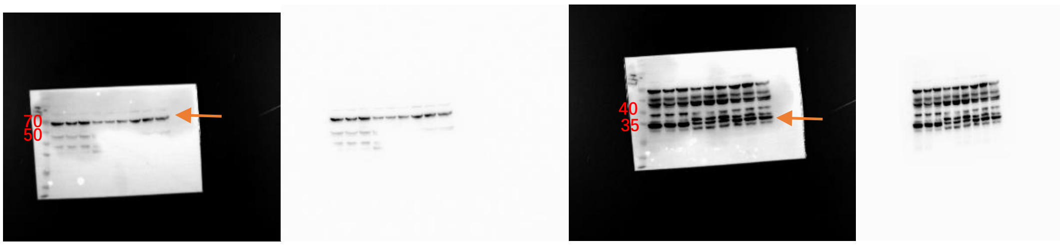

p-AKT p-AKT GAPDH GAPDH

AKT (From left to right, 1-3: PCSK9$^{(+/+)}$; 4-6: PCSK9$^{(-/-)}$; 7-9: PCSK9$^{(+/-)}$)

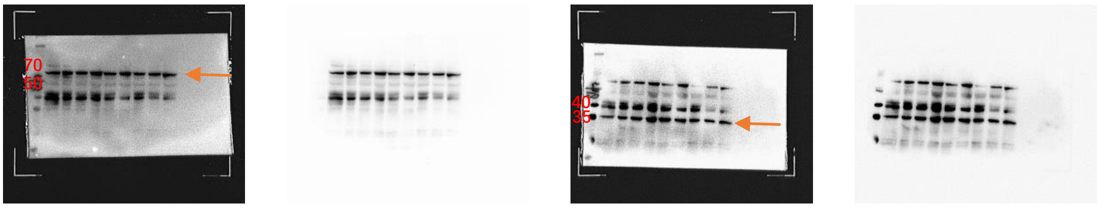

AKT AKT GAPDH GAPDH

Figure 3L p-mTOR (From left to right, 1-3: PCSK9$^{(+/+)}$; 4-6: PCSK9$^{(-/-)}$; 7-9: PCSK9$^{(+/-)}$)

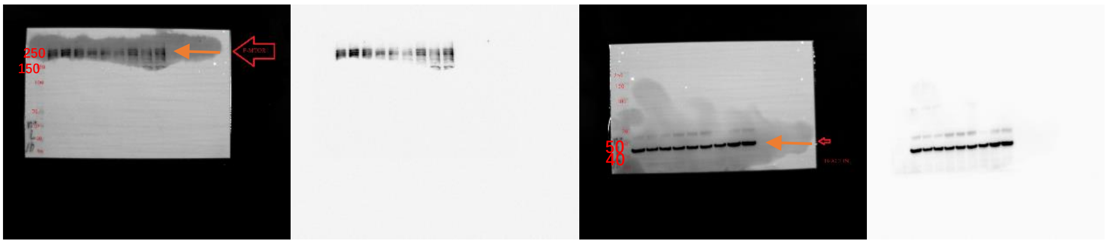

p-mTOR p-mTOR β-actin β-actin

mTOR (From left to right, 1-3: PCSK9$^{(+/+)}$; 4-6: PCSK9$^{(-/-)}$; 7-9: PCSK9$^{(+/-)}$)

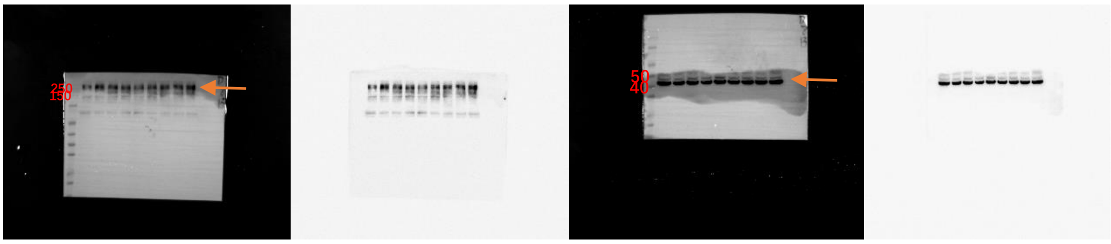

mTOR mTOR β-actin β-actin

Figure 4A PCSK9 (From left to right, 1-3: Blank; 4-6: NC; 7-9: PCSK9 siRNA1;10-12: PCSK9 siRNA2)

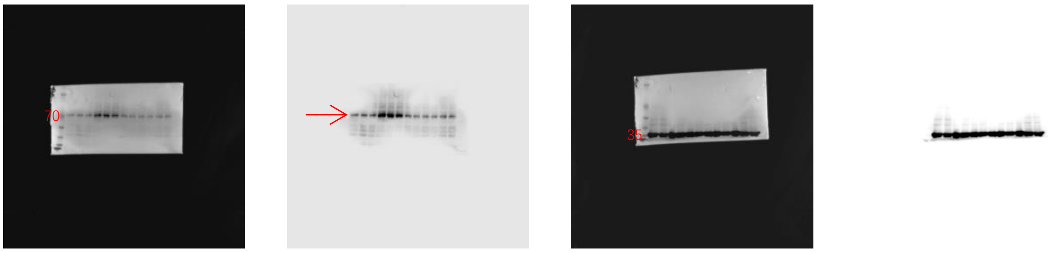

PCSK9 PCSK9 GAPDH GAPDH

Figure 4B TNFα (From left to right, 1-3: Blank; 4-6: NC; 7-9: PCSK9 siRNA1;10-12: PCSK9 siRNA2)

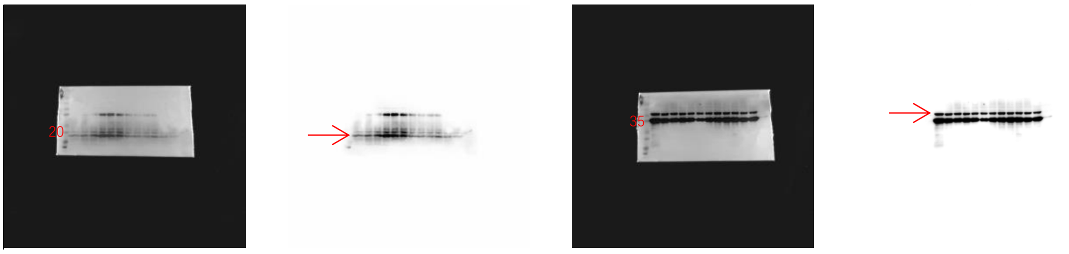

TNFα TNFα β-actin β-actin

Figure 4C IL-6 (From left to right, 1-3: Blank; 4-6: NC; 7-9: PCSK9 siRNA1;10-12: PCSK9 siRNA2)

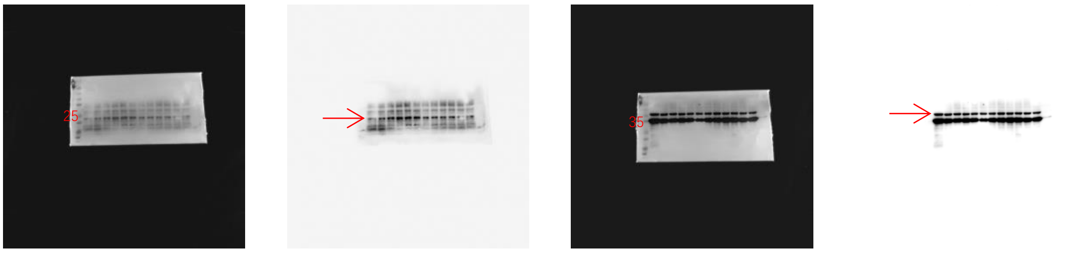

IL-6 IL-6 β-actin β-actin

Figure 4D IL-1β (From left to right, 1-3: Blank; 4-6: NC; 7-9: PCSK9 siRNA1;10-12: PCSK9 siRNA2)

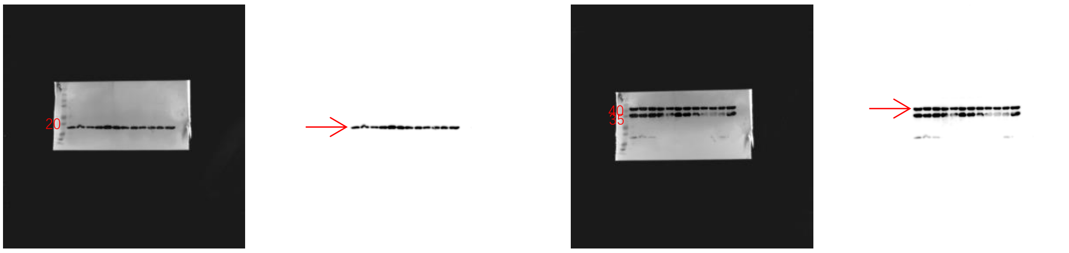

IL-1β IL-1β β-actin β-actin

Figure 4E TLR2 (From left to right, 1-3: Blank; 4-6: NC; 7-9: PCSK9 siRNA1;10-12: PCSK9 siRNA2)

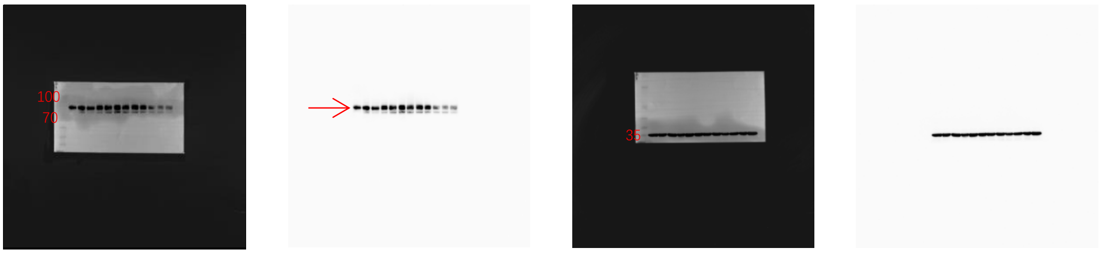

TLR2 TLR2 GAPDH GAPDH

Figure 4F TLR4 (From left to right, 1-3: Blank; 4-6: NC; 7-9: PCSK9 siRNA1;10-12: PCSK9 siRNA2)

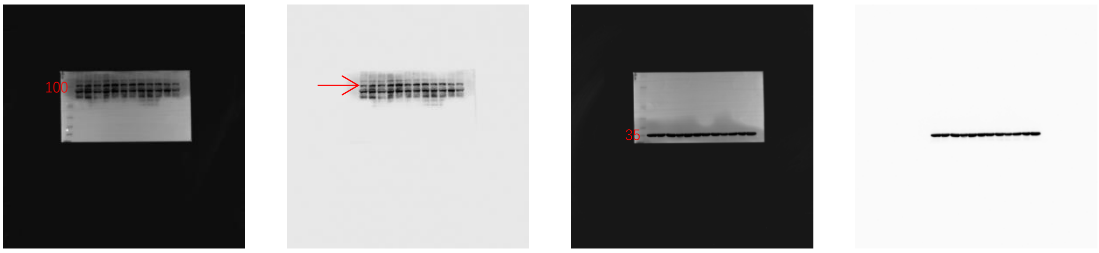

TLR4 TLR4 GAPDH GAPDH

Figure 4G p-MyD88, MyD88 (From left to right, 1-3: Blank; 4-6: NC; 7-9: PCSK9 siRNA1;10-12: PCSK9 siRNA2)

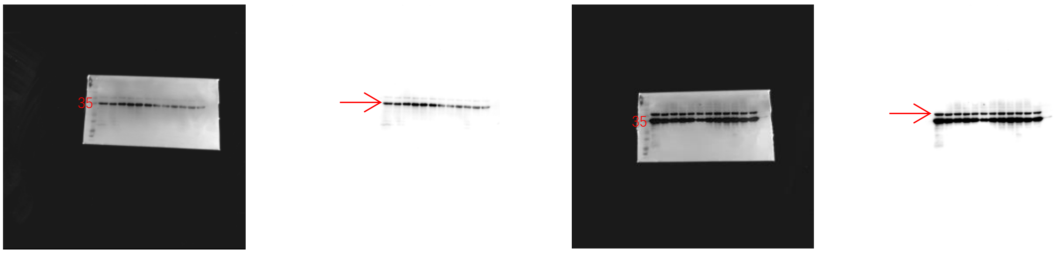

p-MyD88 p-MyD88 β-actin β-actin

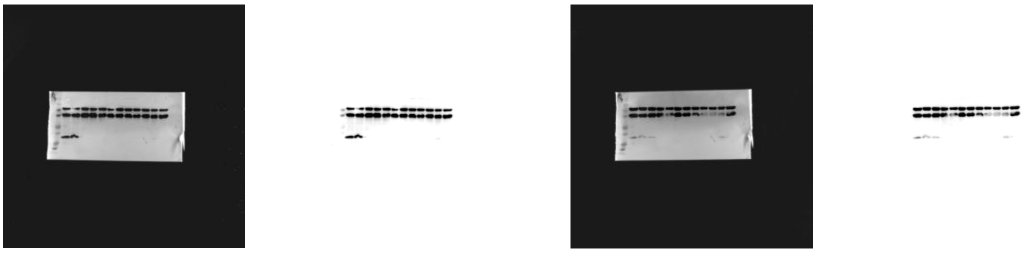

MyD88 MyD88 β-actin β-actin

Figure 4H p-NF-κB P65, NF-κB P65 (From left to right, 1-3: Blank; 4-6: NC; 7-9: PCSK9 siRNA1;10-12: PCSK9 siRNA2)

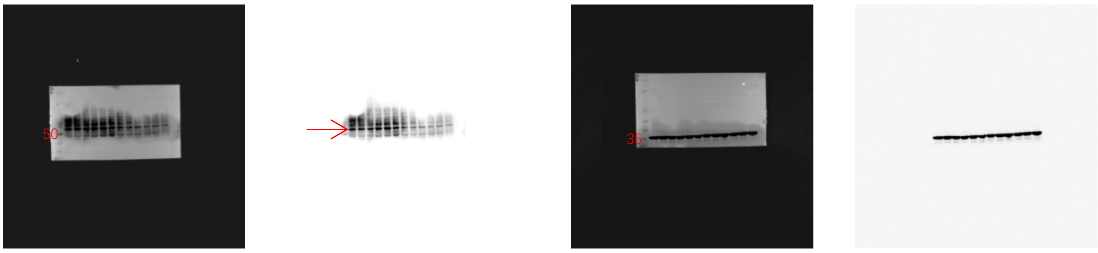

p-NF-κB P65 p-NF-κB P65 GAPDH GAPDH

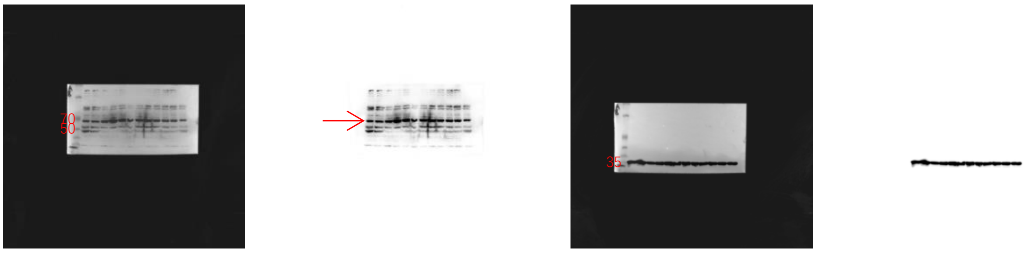

NF-κB P65 NF-κB P65 GAPDH GAPDH

Figure 4I p-AP-1, AP-1 (From left to right, 1-3: Blank; 4-6: NC; 7-9: PCSK9 siRNA1;10-12: PCSK9 siRNA2)

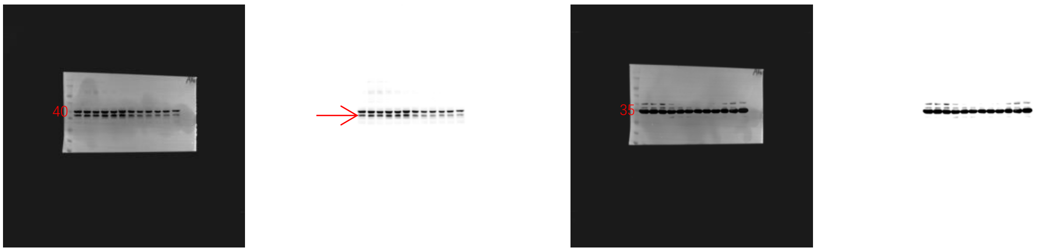

p-AP-1 p-AP-1 GAPDH GAPDH

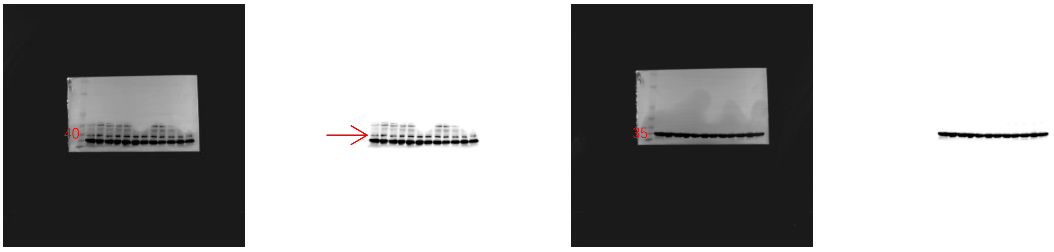

AP-1 AP-1 GAPDH GAPDH

Figure 4J p-P38, P38 (From left to right, 1-3: Blank; 4-6: NC; 7-9: PCSK9 siRNA1;10-12: PCSK9 siRNA2)

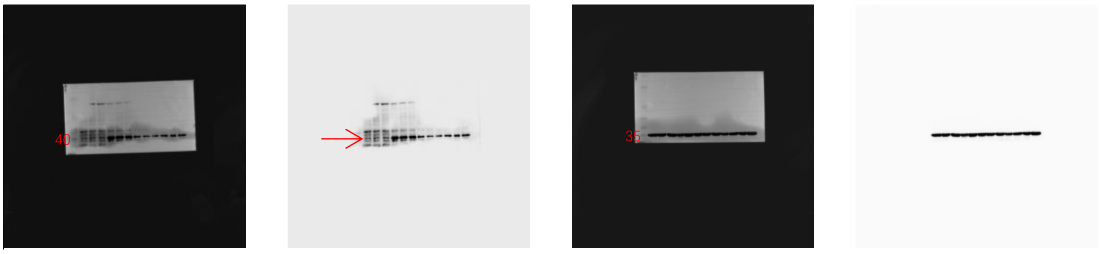

p-P38 p-P38 GAPDH GAPDH

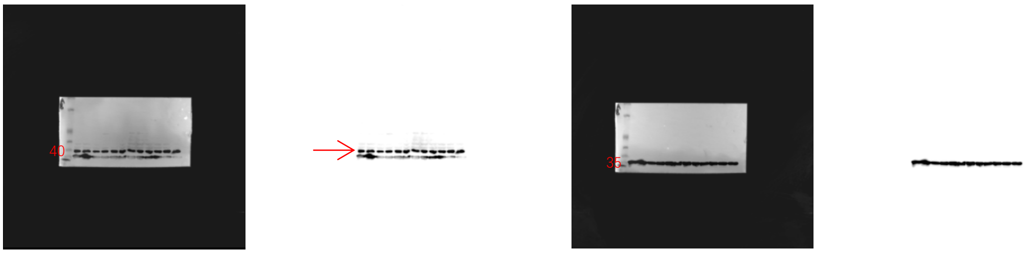

P38 P38 GAPDH GAPDH

Figure 4K p-ERK1/2, ERK (From left to right, 1-3: Blank; 4-6: NC; 7-9: PCSK9 siRNA1;10-12: PCSK9 siRNA2)

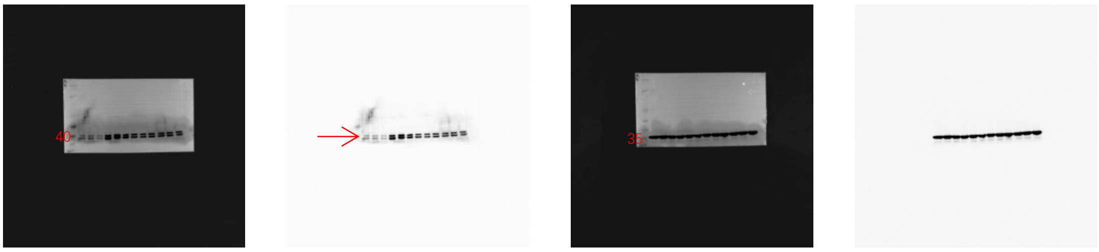

p-ERK1/2 p-ERK1/2 GAPDH GAPDH

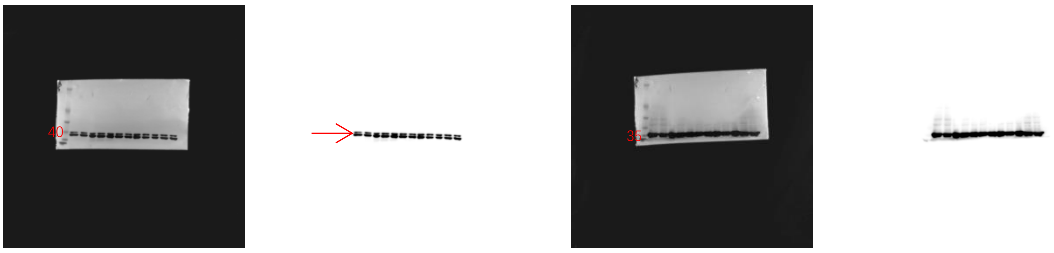

ERK ERK GAPDH GAPDH

Figure 4L p-JNK, JNK (From left to right, 1-3: Blank; 4-6: NC; 7-9: PCSK9 siRNA1;10-12: PCSK9 siRNA2)

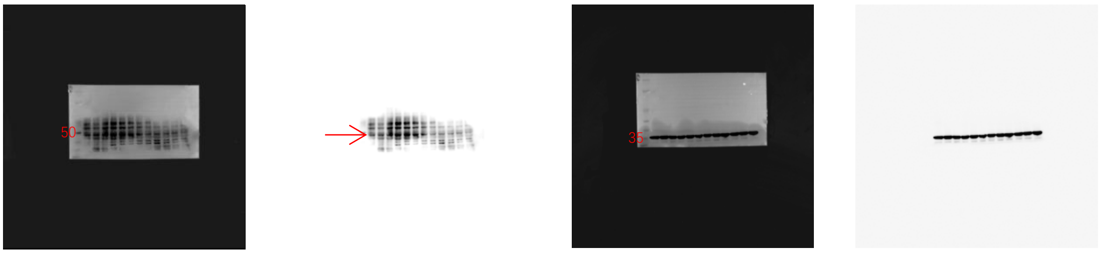

p-JNK p-JNK GAPDH GAPDH

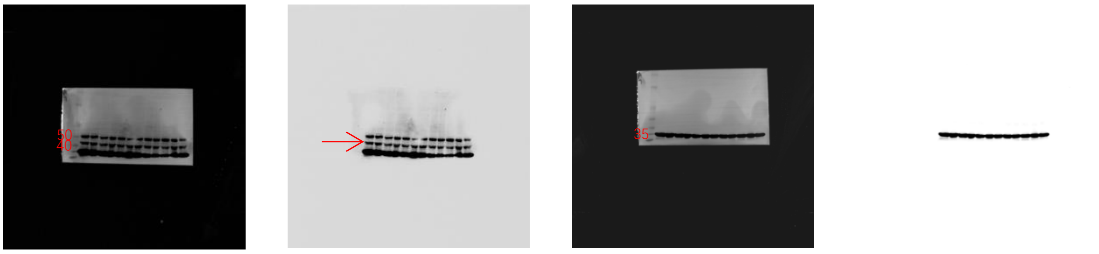

JNK JNK GAPDH GAPDH

Figure 4M p-PI3K, PI3K (From left to right, 1-3: Blank; 4-6: NC; 7-9: PCSK9 siRNA1;10-12: PCSK9 siRNA2)

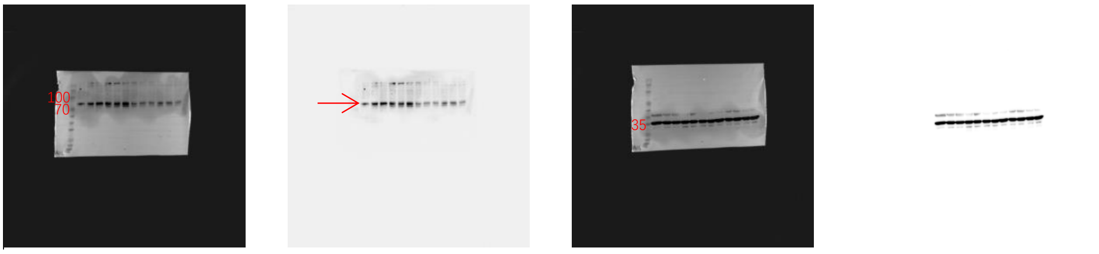

p-PI3K p-PI3K GAPDH GAPDH

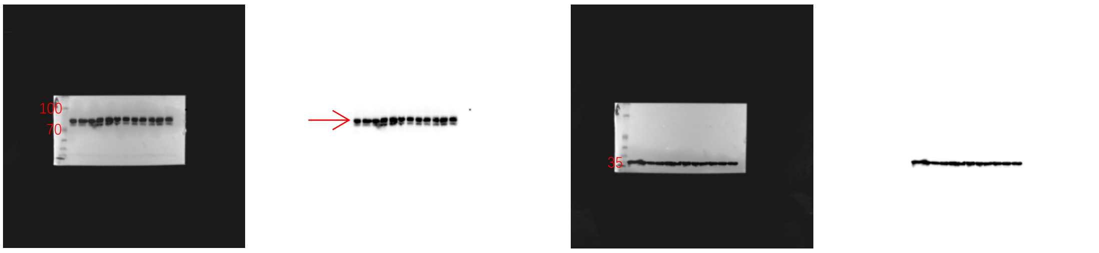

PI3K PI3K GAPDH GAPDH

Figure 4N p-AKT, AKT (From left to right, 1-3: Blank; 4-6: NC; 7-9: PCSK9 siRNA1;10-12: PCSK9 siRNA2)

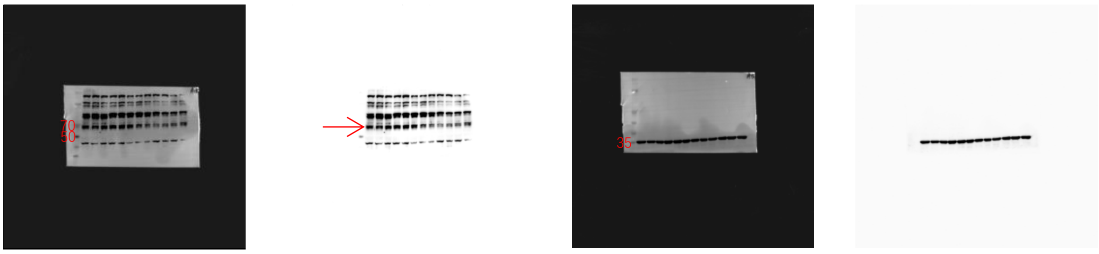

p-AKT p-AKT GAPDH GAPDH

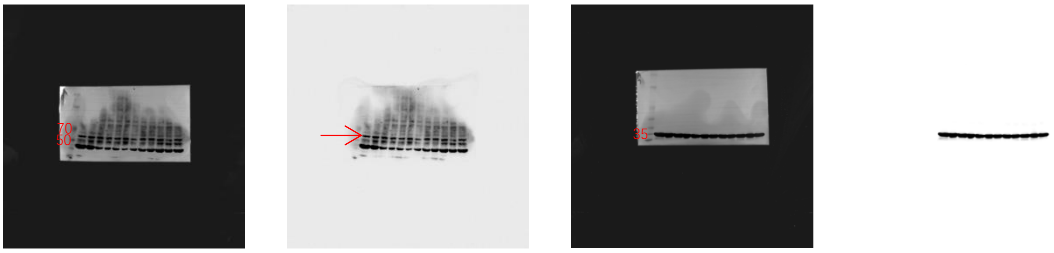

AKT AKT GAPDH GAPDH

Figure 4O p-mTOR, mTOR (From left to right, 1-3: Blank; 4-6: NC; 7-9: PCSK9 siRNA1;10-12: PCSK9 siRNA2)

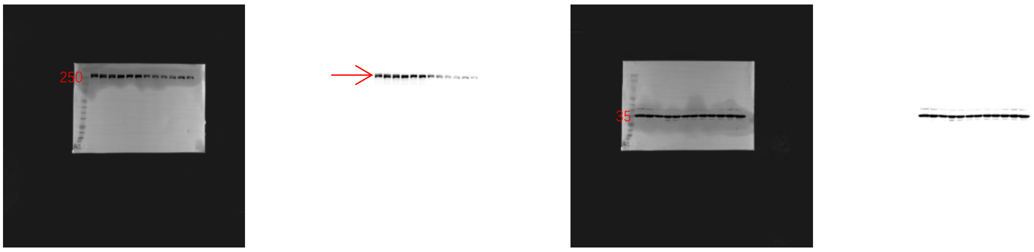

p-mTOR p-mTOR GAPDH GAPDH

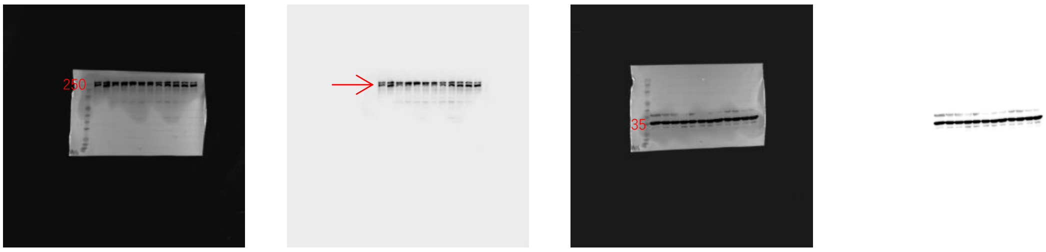

mTOR mTOR GAPDH GAPDH

Figure 5A PCSK9 (From left to right, 1-2: BC; 3-4: Mod; 4-6: Pm; 7-8: P38i; 9-10: Pm+i)

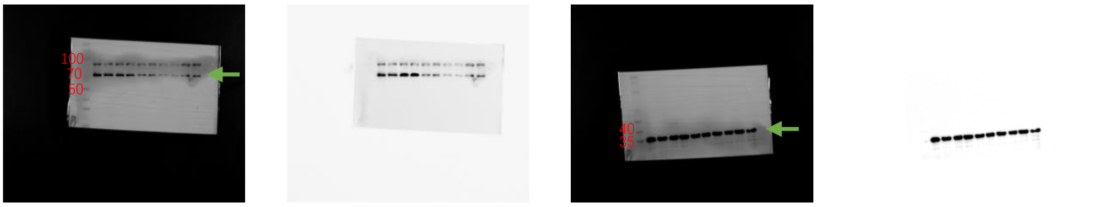

PCSK9 PCSK9 GAPDH GAPDH

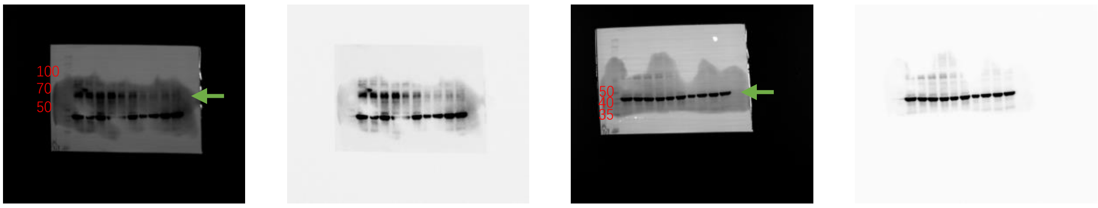

PCSK9 PCSK9 β-actin β-actin

Figure 5B LDLR (From left to right, 1-2: BC; 3-4: Mod; 4-6: Pm; 7-8: P38i; 9-10: Pm+i)

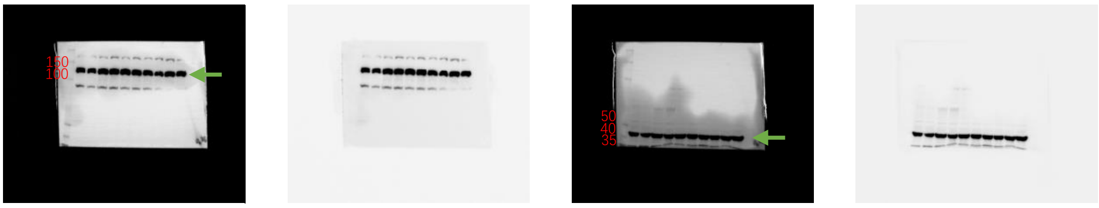

LDLR LDLR β-actin β-actin

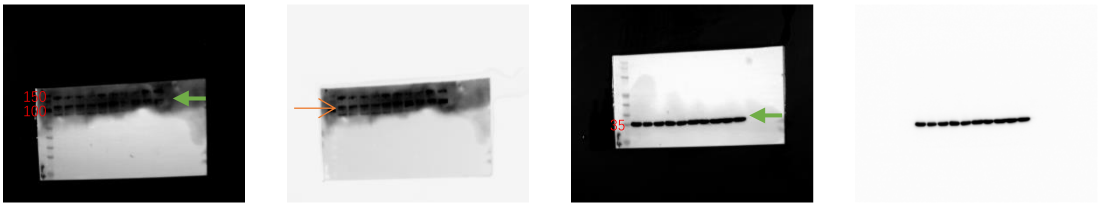

LDLR LDLR GAPDH GAPDH

Figure 5C IL-6 (From left to right, 1-2: BC; 3-4: Mod; 4-6: Pm; 7-8: P38i; 9-10: Pm+i)

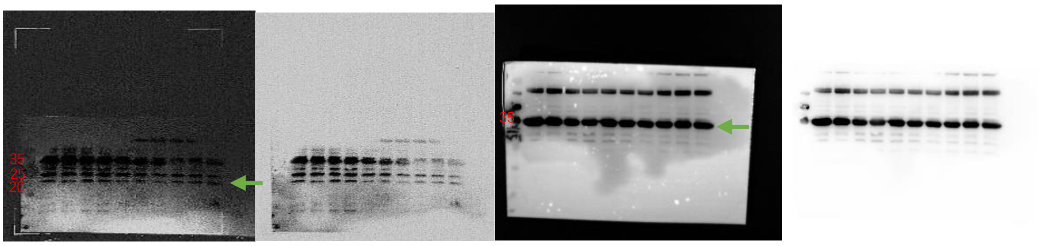

IL-6 IL-6 GAPDH GAPDH

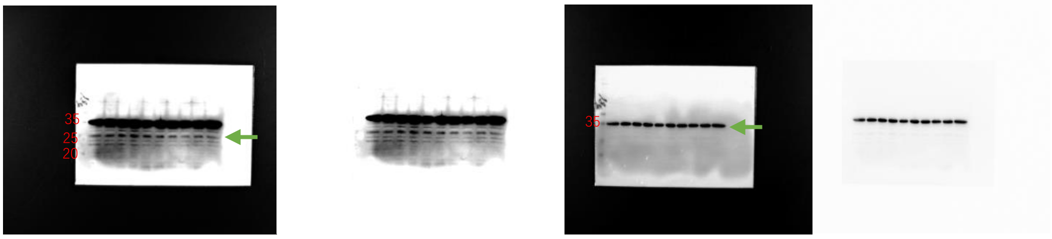

IL-6 IL-6 GAPDH GAPDH

Figure 5D IL-1β (From left to right, 1-2: BC; 3-4: Mod; 4-6: Pm; 7-8: P38i; 9-10: Pm+i)

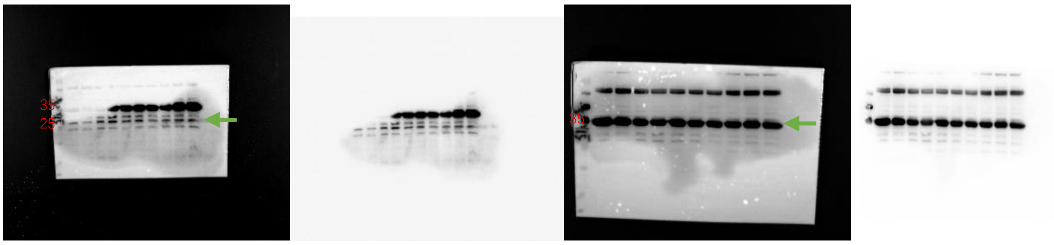

IL-1β IL-1β GAPDH GAPDH

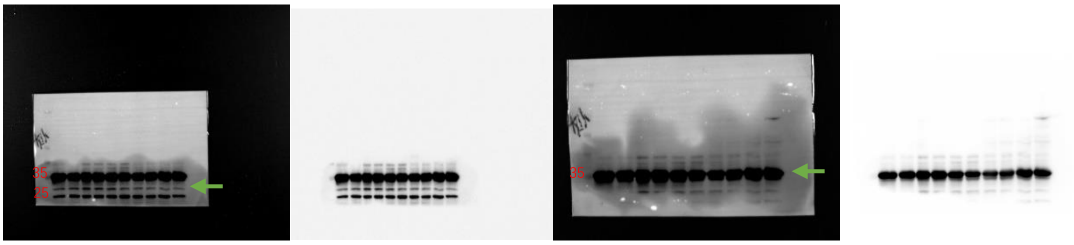

IL-1β IL-1β GAPDH GAPDH

Figure 5E TNFα (From left to right, 1-2: BC; 3-4: Mod; 4-6: Pm; 7-8: P38i; 9-10: Pm+i)

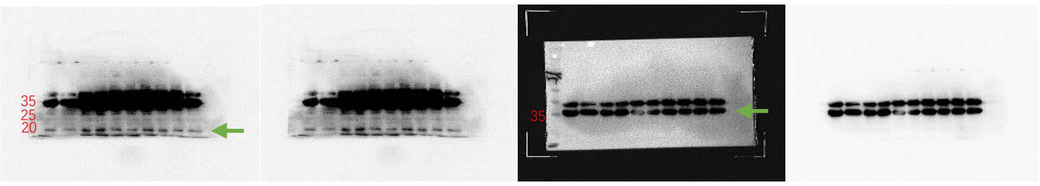

TNFα TNFα GAPDH GAPDH

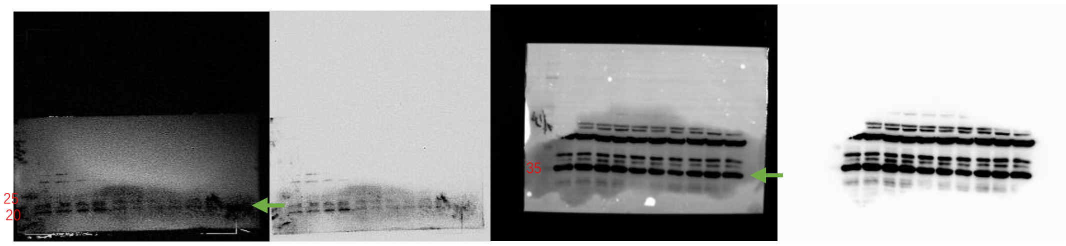

TNFα TNFα GAPDH GAPDH

Figure 5F TLR4 (From left to right, 1-2: BC; 3-4: Mod; 4-6: Pm; 7-8: P38i; 9-10: Pm+i)

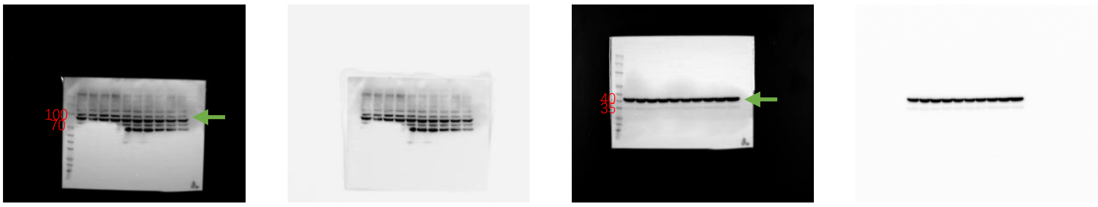

TLR4 TLR4 GAPDH GAPDH

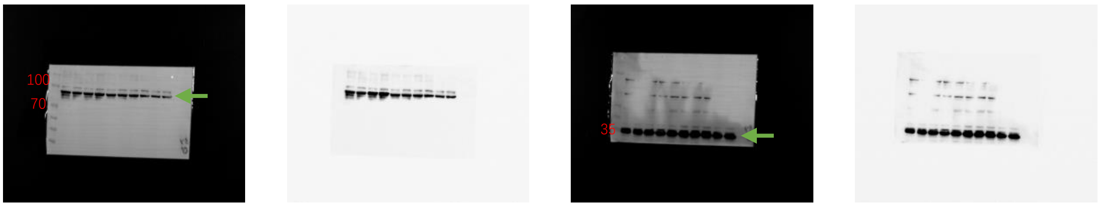

TLR4 TLR4 GAPDH GAPDH

Figure 5G TLR2 (From left to right, 1-2: BC; 3-4: Mod; 4-6: Pm; 7-8: P38i; 9-10: Pm+i)

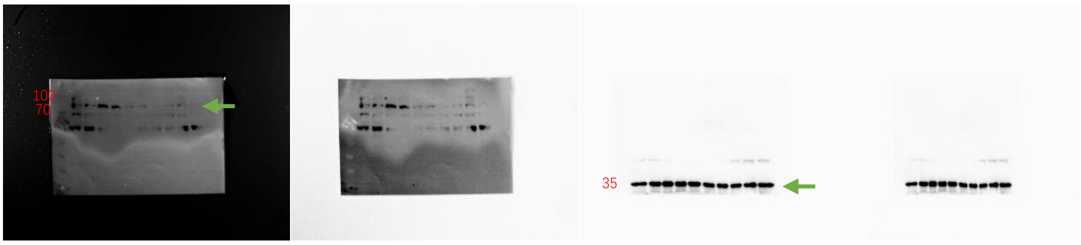

TLR2 TLR2 GAPDH GAPDH

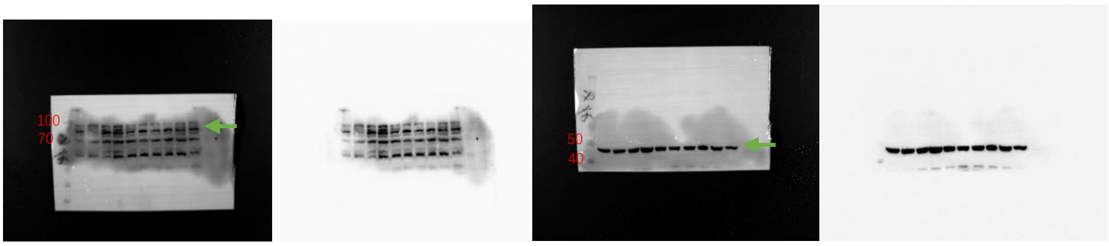

TLR2 TLR2 β-actin β-actin

Figure 5H MyD88 (From left to right, 1-2: BC; 3-4: Mod; 4-6: Pm; 7-8: P38i; 9-10: Pm+i)

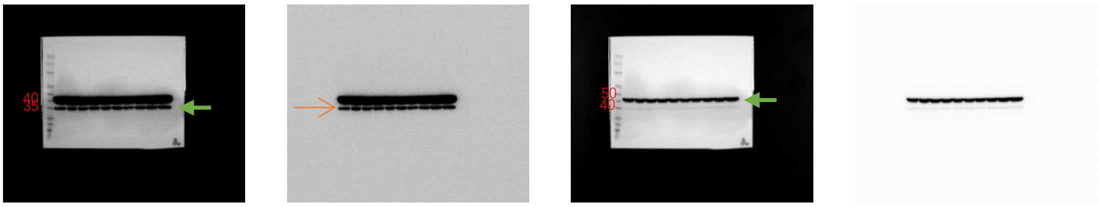

MyD88 MyD88 β-actin β-actin

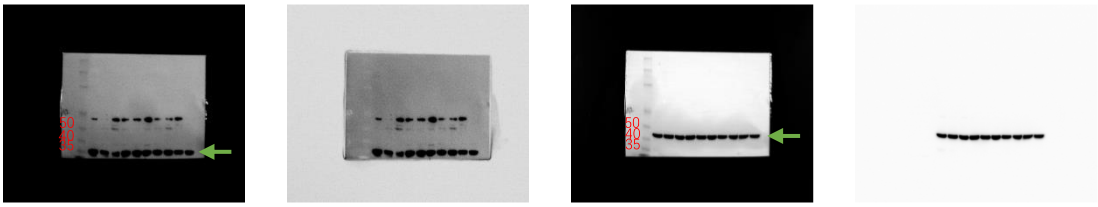

MyD88 MyD88 β-actin β-actin

p-MyD88 (From left to right, 1-2: BC; 3-4: Mod; 4-6: Pm; 7-8: P38i; 9-10: Pm+i)

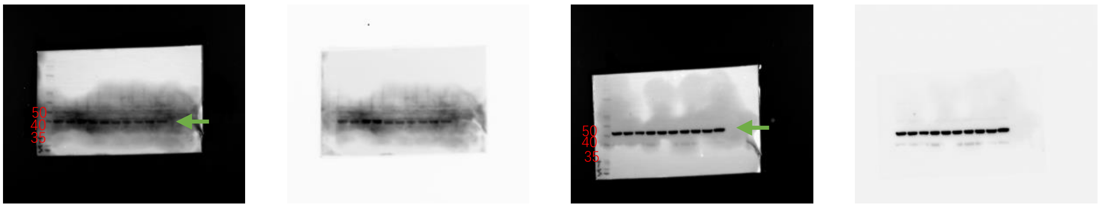

p-MyD88 p-MyD88 β-actin β-actin

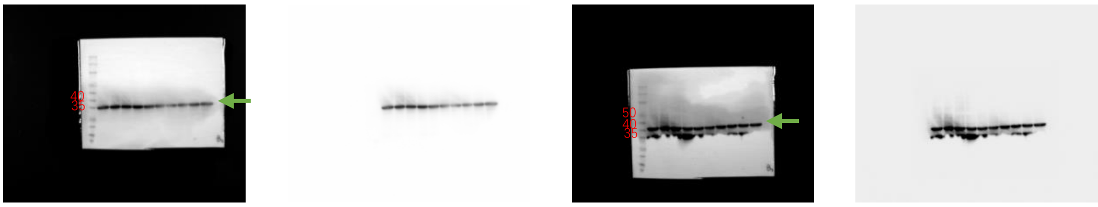

p-MyD88 p-MyD88 β-actin β-actin

Figure 5I NF-κB P65 (From left to right, 1-2: BC; 3-4: Mod; 4-6: Pm; 7-8: P38i; 9-10: Pm+i)

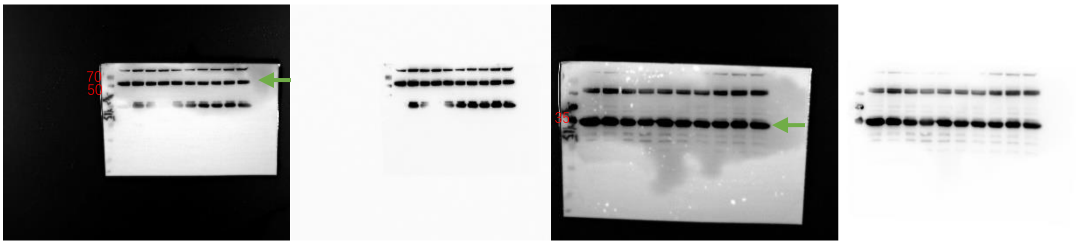

NF-κB P65 NF-κB P65 GAPDH GAPDH

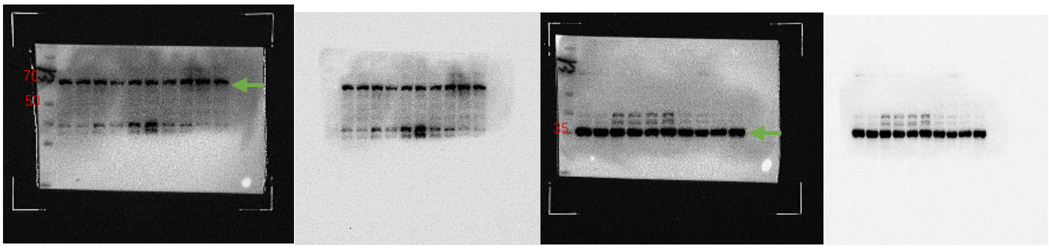

NF-κB P65 NF-κB P65 GAPDH GAPDH

p- NF-κB P65 (From left to right, 1-2: BC; 3-4: Mod; 4-6: Pm; 7-8: P38i; 9-10: Pm+i)

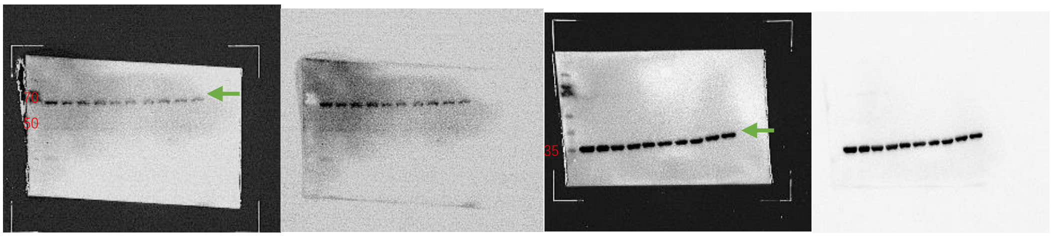

p-NF-κB P65 p-NF-κB P65 GAPDH GAPDH

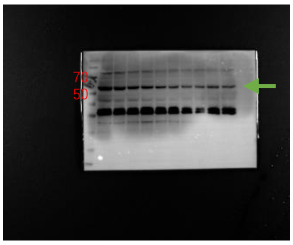 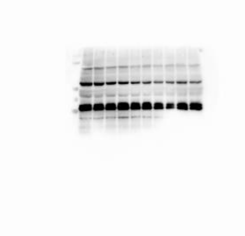 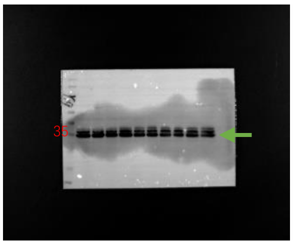 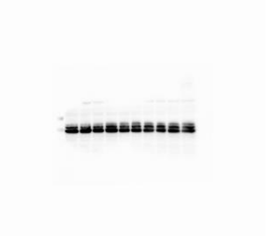

p-NF-κB P65 p-NF-κB P65 GAPDH GAPDH

Figure 5J AP-1 (From left to right, 1-2: BC; 3-4: Mod; 4-6: Pm; 7-8: P38i; 9-10: Pm+i)

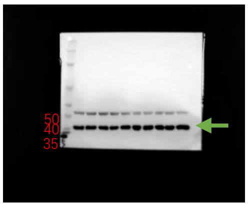 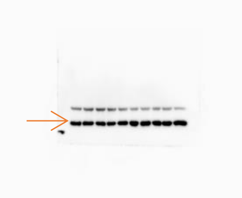 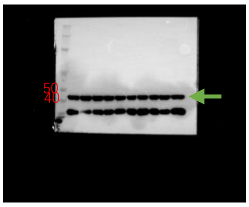 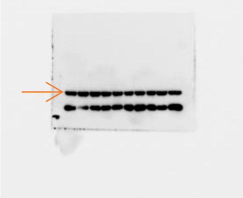

AP-1 AP-1 β-actin β-actin

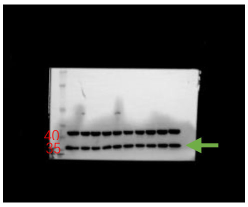 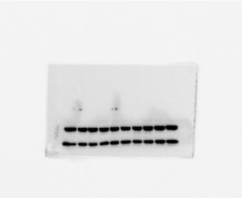 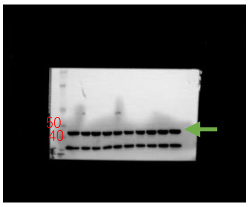 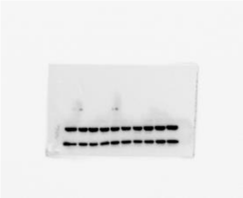

AP-1 AP-1 β-actin β-actin

p-AP-1 (From left to right, 1-2: BC; 3-4: Mod; 4-6: Pm; 7-8: P38i; 9-10: Pm+i)

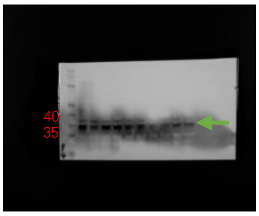 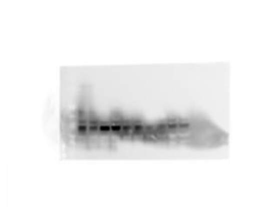 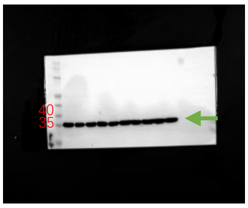

p-AP-1 p-AP-1 GAPDH GAPDH

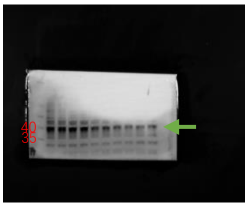 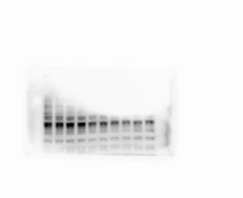 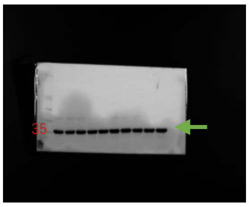

p-AP-1 p-AP-1 GAPDH GAPDH

Figure 5K P38 MAPK (From left to right, 1-2: BC; 3-4: Mod; 4-6: Pm; 7-8: P38i; 9-10: Pm+i)

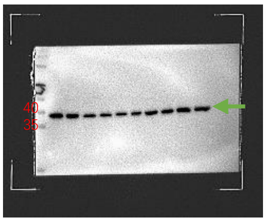 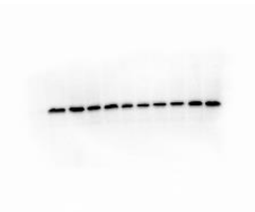 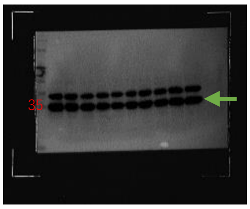 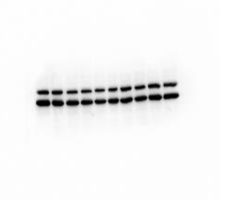

P38 MAPK P38 MAPK GAPDH GAPDH

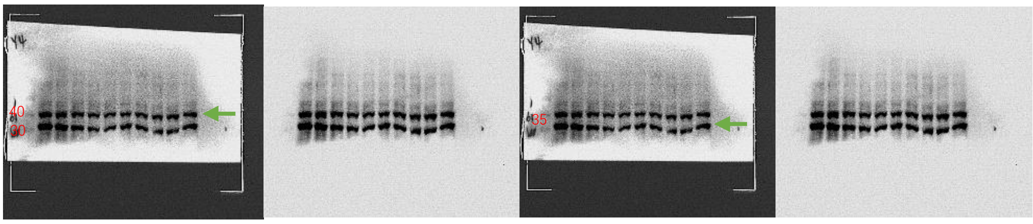

P38 MAPK P38 MAPK GAPDH GAPDH

p-P38 MAPK (From left to right, 1-2: BC; 3-4: Mod; 4-6: Pm; 7-8: P38i; 9-10: Pm+i)

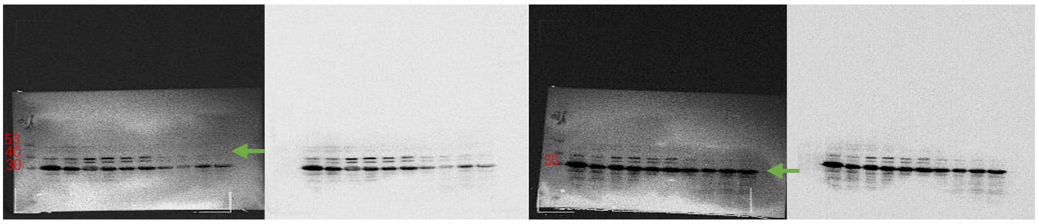

p-P38 MAPK p-P38 MAPK GAPDH GAPDH

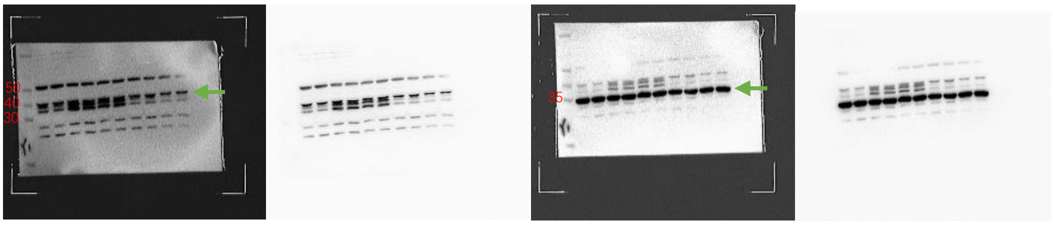

p-P38 MAPK p-P38 MAPK GAPDH GAPDH

Figure 5L ERK1/2 (From left to right, 1-2: BC; 3-4: Mod; 4-6: Pm; 7-8: P38i; 9-10: Pm+i)

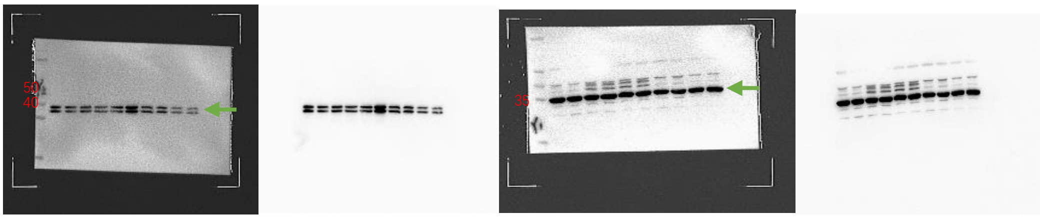

ERK1/2 ERK1/2 GAPDH GAPDH

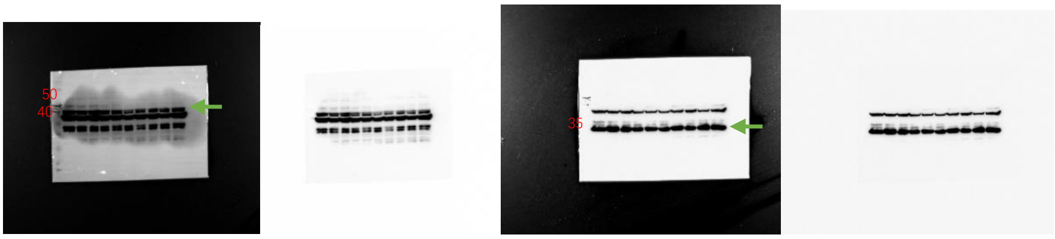

ERK1/2 ERK1/2 GAPDH GAPDH

p-ERK1/2 (From left to right, 1-2: BC; 3-4: Mod; 4-6: Pm; 7-8: P38i; 9-10: Pm+i)

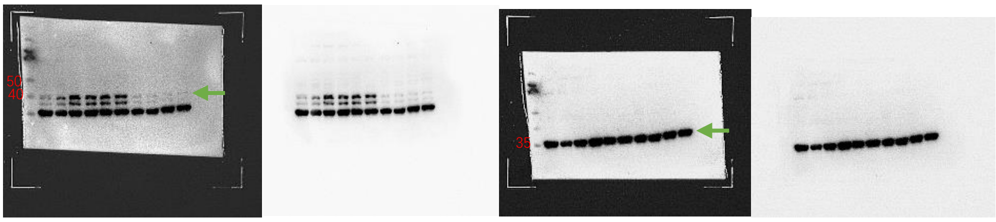

p-ERK1/2 p-ERK1/2 GAPDH GAPDH

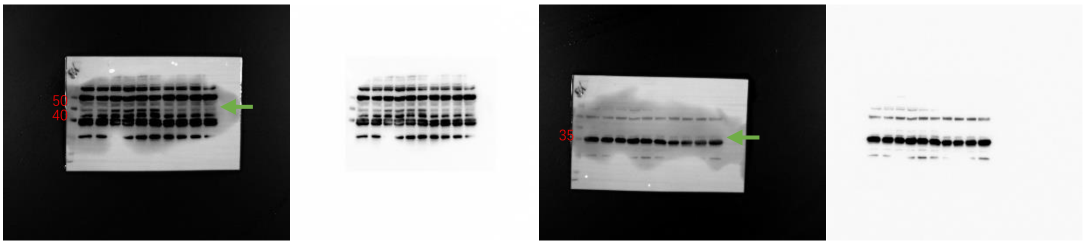

p-ERK1/2 p-ERK1/2 GAPDH GAPDH

Figure 5M JNK (From left to right, 1-2: BC; 3-4: Mod; 4-6: Pm; 7-8: P38i; 9-10: Pm+i)

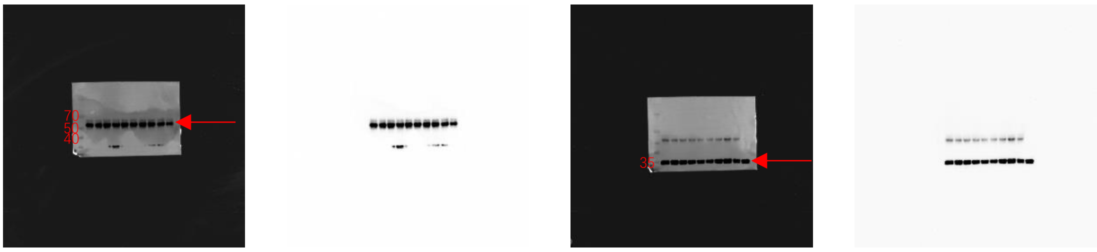

JNK JNK GAPDH GAPDH

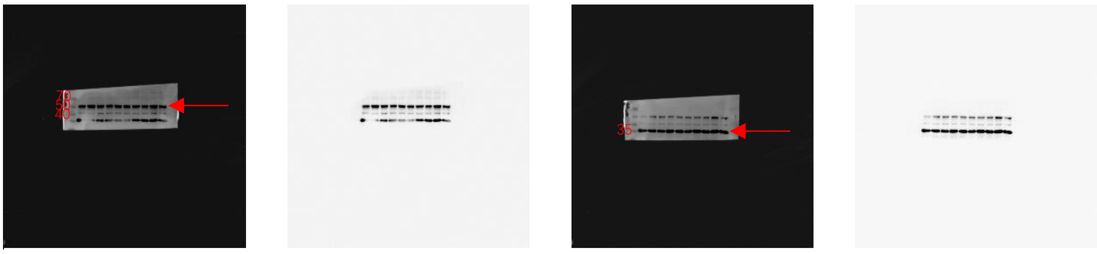

JNK JNK GAPDH GAPDH

p-JNK (From left to right, 1-2: BC; 3-4: Mod; 4-6: Pm; 7-8: P38i; 9-10: Pm+i)

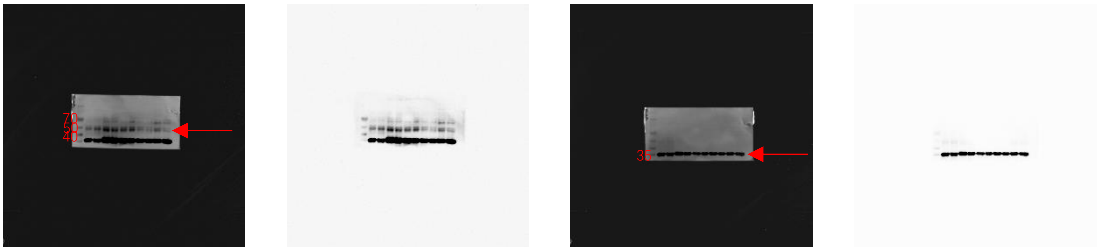

p-JNK p-JNK GAPDH GAPDH

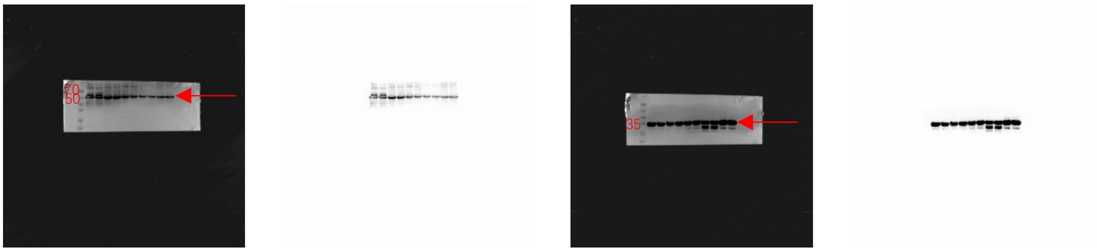

p-JNK p-JNK GAPDH GAPDH

Figure 5N PI3K (From left to right, 1-2: BC; 3-4: Mod; 4-6: Pm; 7-8: P38i; 9-10: Pm+i)

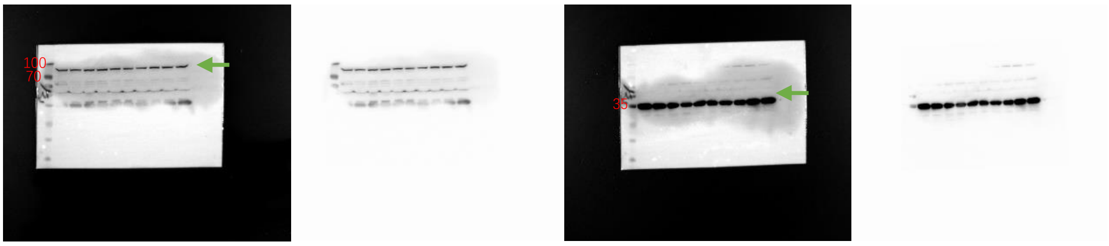

PI3K PI3K GAPDH GAPDH

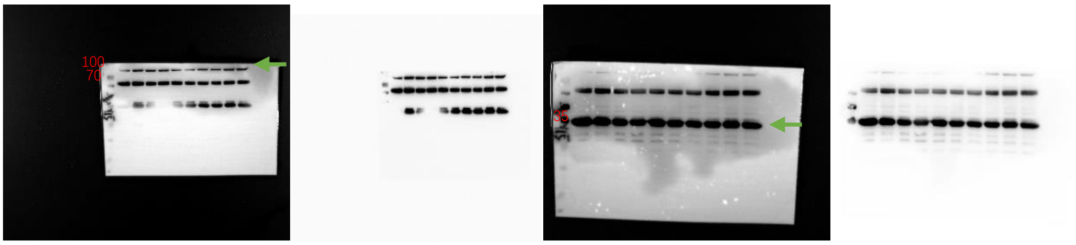

PI3K PI3K GAPDH GAPDH

p-PI3K (From left to right, 1-2: BC; 3-4: Mod; 4-6: Pm; 7-8: P38i; 9-10: Pm+i)

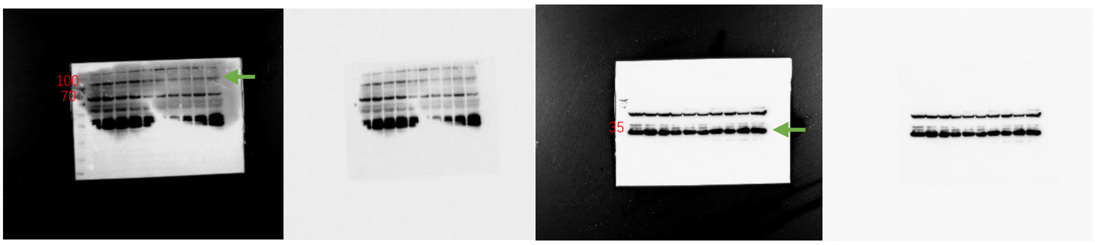

p-PI3K p-PI3K GAPDH GAPDH

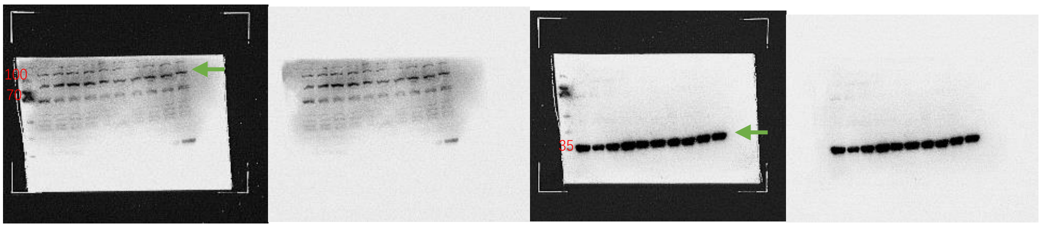

p-PI3K p-PI3K GAPDH GAPDH

Figure 5O AKT (From left to right, 1-2: BC; 3-4: Mod; 4-6: Pm; 7-8: P38i; 9-10: Pm+i)

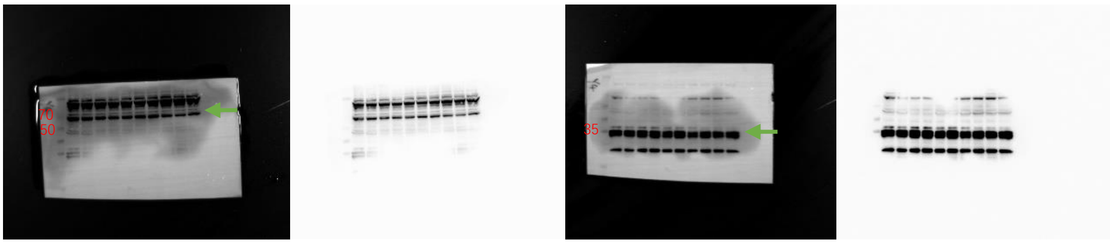

AKT AKT GAPDH GAPDH

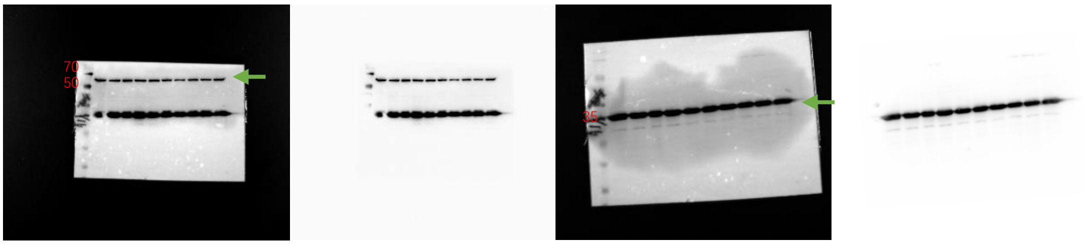

AKT AKT GAPDH GAPDH

p-AKT (From left to right, 1-2: BC; 3-4: Mod; 4-6: Pm; 7-8: P38i; 9-10: Pm+i)

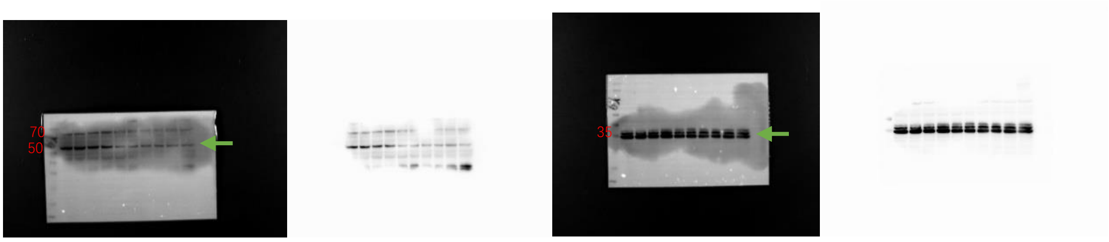

p-AKT p-AKT GAPDH GAPDH

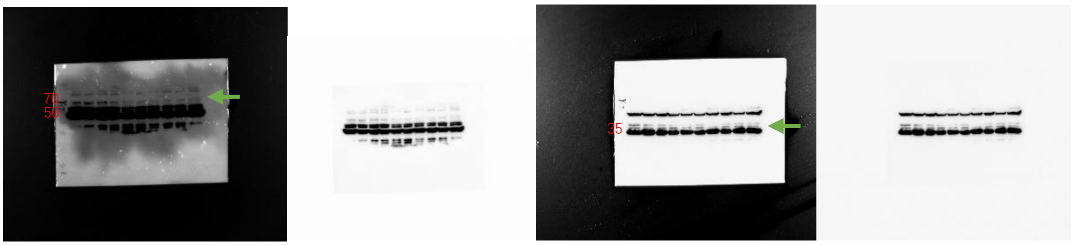

p-AKT p-AKT GAPDH GAPDH

Figure 5P mTOR (From left to right, 1-2: BC; 3-4: Mod; 4-6: Pm; 7-8: P38i; 9-10: Pm+i)

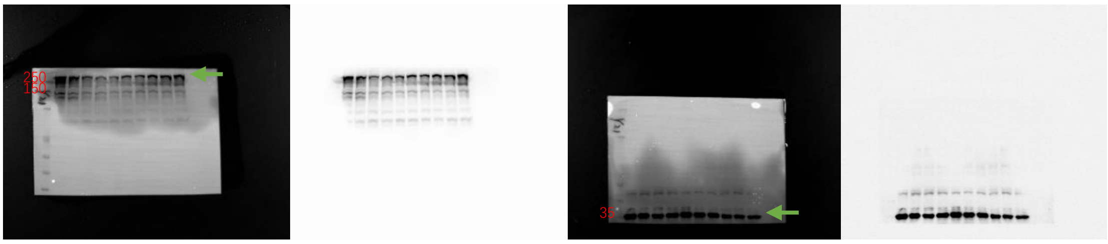

mTOR mTOR GAPDH GAPDH

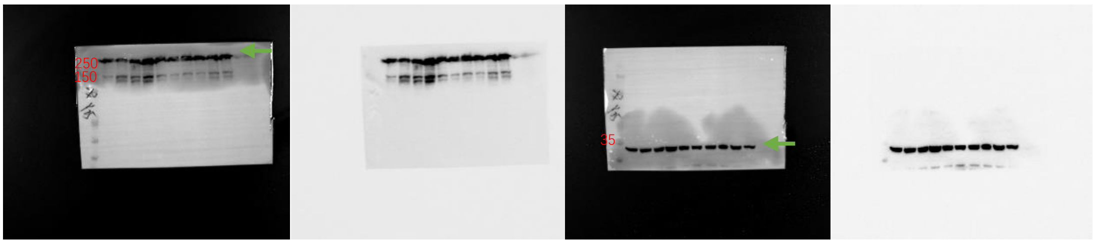

mTOR mTOR GAPDH GAPDH

p-mTOR (From left to right, 1-2: BC; 3-4: Mod; 4-6: Pm; 7-8: P38i; 9-10: Pm+i)

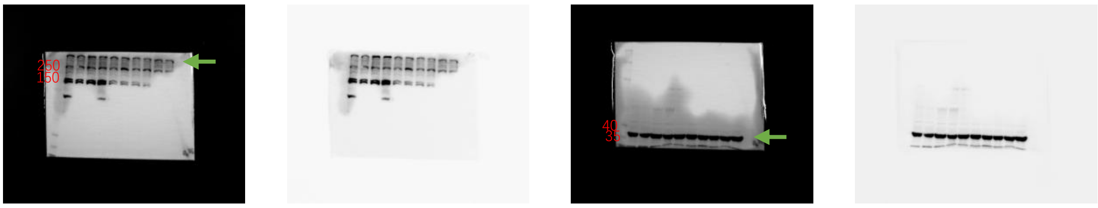

p-mTOR p-mTOR β-actin β-actin

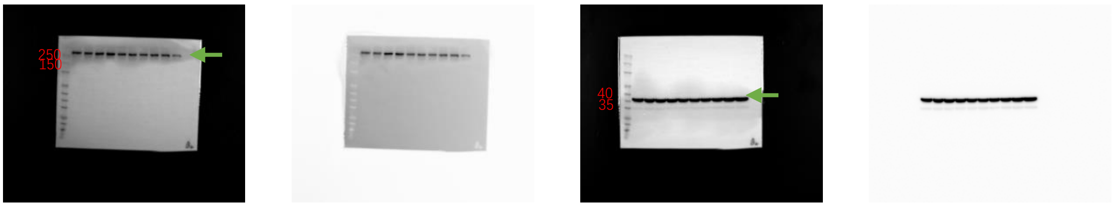

p-mTOR p-mTOR GAPDH GAPDH
