# Supplementary material for: Conditional knockdown of hepatic PCSK9 ameliorates high-fat diet-induced liver inflammation in mice
Source: Front Pharmacol. 2025 Feb 3;16:1528250. doi: 10.3389/fphar.2025.1528250 (PMC11830812; doi:10.3389/fphar.2025.1528250)
Supplement: Supplementary file 1 [file Presentation1.zip › Data Sheet 1/Supplementary Table 1.docx]

**Supplementary Table 1. Antibody information.**

| Name and molecular weight | Company | Cat number |
| --- | --- | --- |
| Rabbit polyclonal antibody against PCSK9 (70kDa) | ABclonal (Wuhan, China) | A24478 |
| Rabbit polyclonal antibody against LDLR (120kDa) | Santa Cruz (U.S.A.) | bs-0705R |
| Rabbit polyclonal antibody against TNFα (25kDa) | Santa Cruz (U.S.A.) | 3033 |
| Mouse monoclonal antibody against c-Jun N-terminal kinase (JNK) (46kDa) | Santa Cruz (U.S.A.) | sc-7345 |
| Mouse monoclonal antibody against phosphoinositide-3 kinase (PI3K) (80kDa) | Santa Cruz (U.S.A.) | sc-365290 |
| Mouse monoclonal antibody against IL-6 (21kDa) | Santa Cruz (U.S.A.) | sc-28343 |
| Mouse monoclonal antibody against IL-1β (17kDa) | Cell Signaling (U.S.A.) | 12242 |
| Rabbit monoclonal antibody against p65-NF-κB (65kDa) | Cell Signaling (U.S.A.) | 8242 |
| Rabbit monoclonal antibody against phosphorylated p65-NF-κB (65kDa) | Cell Signaling (U.S.A.) | 3033 |
| Rabbit monoclonal antibody against p38-mitogen-activated protein kinase (MAPK) (40kDa) | Cell Signaling (U.S.A.) | 8690 |
| Rabbit monoclonal antibody against phosphorylated p38-MAPK (40kDa) | Cell Signaling (U.S.A.) | 4511 |
| Rabbit monoclonal antibody against extracellular regulated protein kinase 1/2 (ERK1/2) (42kDa) | Cell Signaling (U.S.A.) | 4695 |
| Rabbit monoclonal antibody against phosphorylated ERK1/2 (42kDa) | Cell Signaling (U.S.A.) | 4370 |
| Rabbit monoclonal antibody against phosphorylated PI3K (80kDa) | Cell Signaling (U.S.A.) | AF3242 |
| Rabbit monoclonal antibody against protein kinase B (AKT/PKB) (65kDa) | Cell Signaling (U.S.A.) | 4691 |
| Rabbit monoclonal antibody against phosphorylated AKT (65kDa) | Cell Signaling (U.S.A.) | 4060 |
| Rabbit monoclonal antibody against mammalian target of rapamycin (mTOR) (289kDa) | Cell Signaling (U.S.A.) | 2983 |
| Rabbit monoclonal antibody against phosphorylated mTOR (289kDa) | Cell Signaling (U.S.A.) | 5536 |
| Rabbit polyclonal antibody against phosphorylated JNK (46kDa) | Signalway Antibody (U.S.A.) | 13371 |
| Rabbit polyclonal antibody against TLR2 (80kDa) | Affinity Biosciences (Jiangsu, China) | DF7002 |
| Rabbit polyclonal antibody against TLR4 (100kDa) | Affinity Biosciences (Jiangsu, China) | AF7017 |
| Rabbit polyclonal antibody against c-Jun/activator protein-1 (AP-1) (37kDa) | Affinity Biosciences (Jiangsu, China) | AF6090 |
| Rabbit polyclonal antibody against phosphorylated c-Jun (p-AP1) (37kDa) | Affinity Biosciences (Jiangsu, China) | AF3090 |
| Rabbit polyclonal antibody against phosphorylated MyD88 (33kDa) | Affinity Biosciences (Jiangsu, China) | AF8490 |
| Mouse monoclonal antibody against MyD88 (33kDa) | Proteintech (U.S.A.) | BC013589 |
| Goat anti-rabbit IgG, HRP conjugated | CWBIO (Beijing, China) | CW0103s |
| Goat anti-mouse IgG, HRP conjugated | CWBIO (Beijing, China) | CW0102s |
