# Supplementary material for: Conditional knockdown of hepatic PCSK9 ameliorates high-fat diet-induced liver inflammation in mice
Source: Front Pharmacol. 2025 Feb 3;16:1528250. doi: 10.3389/fphar.2025.1528250 (PMC11830812; doi:10.3389/fphar.2025.1528250)
Supplement: Supplementary file 2 [file Table1.docx]

**Supplementary Figures**


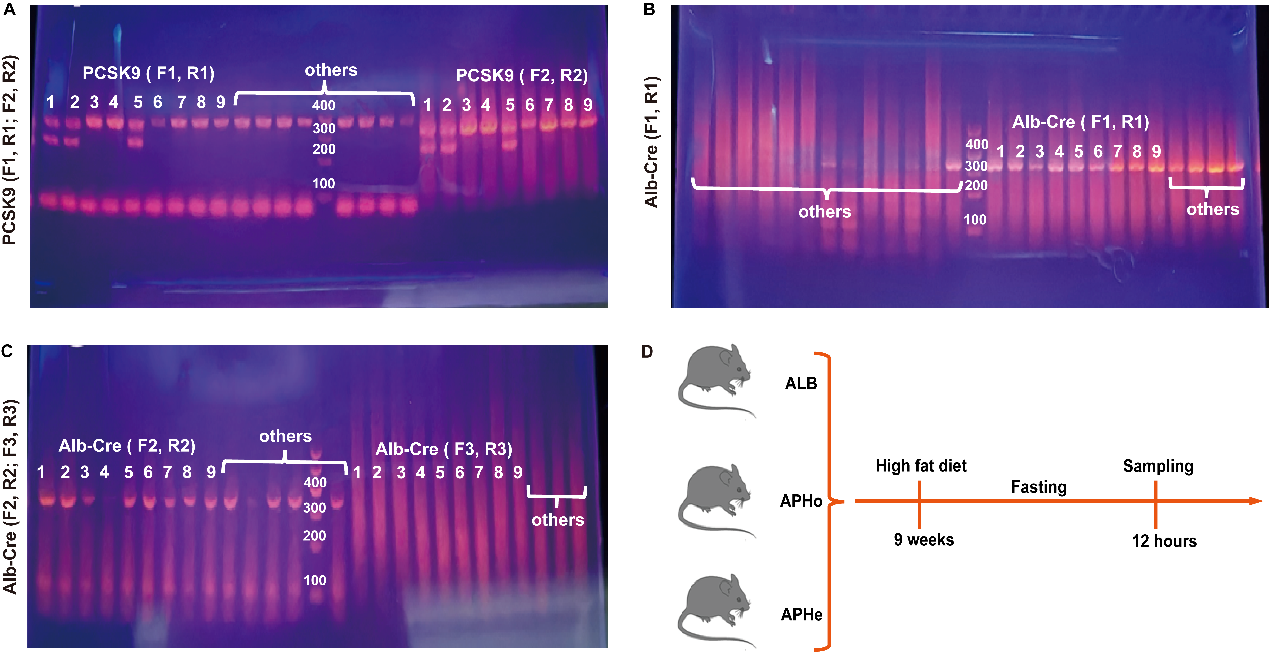


**Supplementary Figure 1.** Genotyping and experimental design for HFD-induced inflammation *in vivo*. In this figure, we only showed the genotyping results of the mice numbered as 1-9. A, results of agarose gel electrophoresis using the primer 1 and 2 for identifying PCSK9; B and C, results of agarose gel electrophoresis using the primer 1, and 2-3 for identifying Alb-Cre, respectively; D, experimental design for HFD-induced inflammation. The mice numbered as 1, 2, and 5 were determined as PCSK9^liver^*^(+/-)^* mice, and the mice numbered as 3, 4, and 6-9 were determined as PCSK9^liver^*^(-/-)^* mice based on the molecular weight of the observed bands.


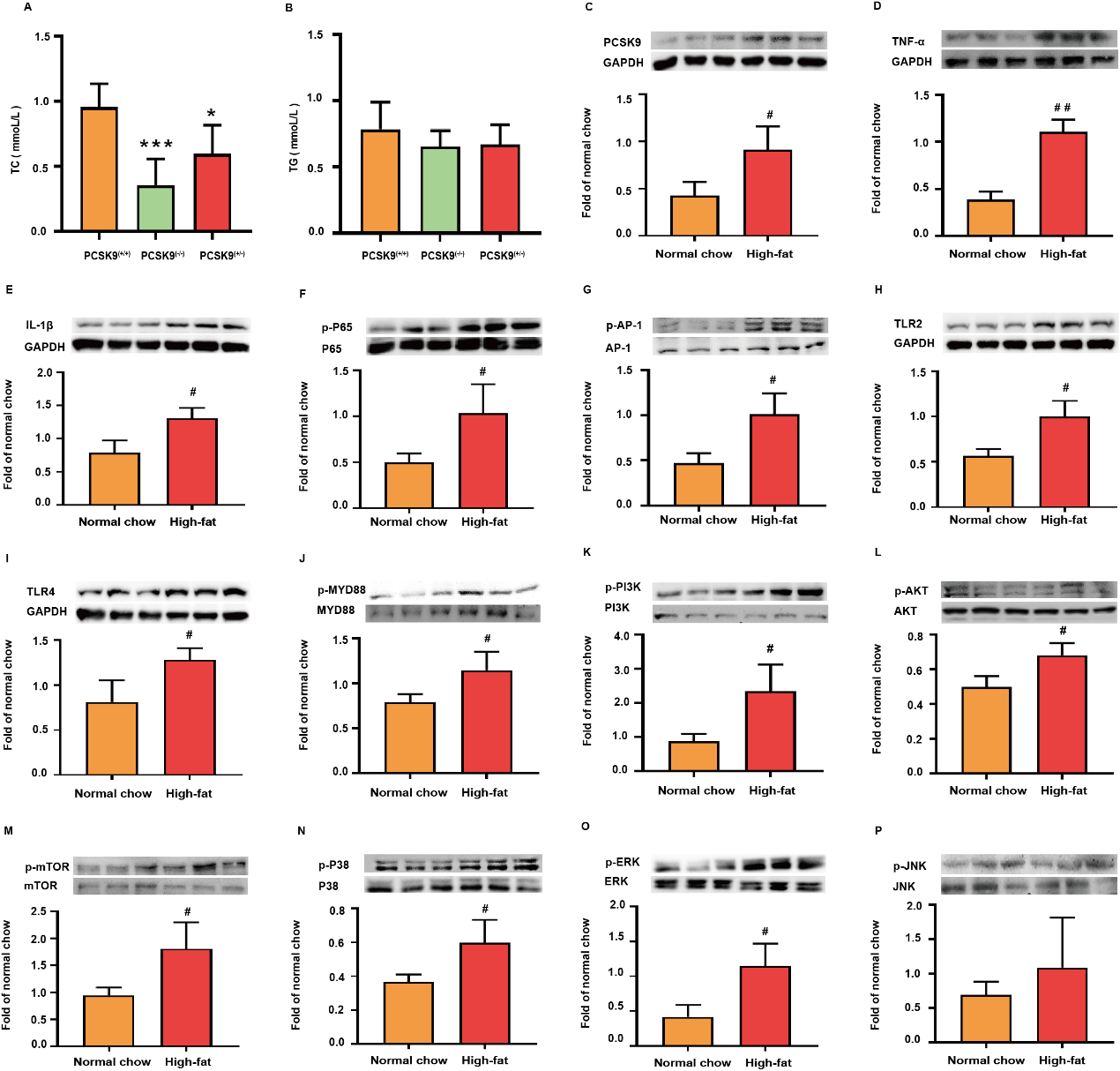


**Supplementary Figure 2.** Effects of HFD on the expression levels of inflammatory factors and the key molecules involved in the TLR/MyD88, MAPK, and PI3K/AKT signaling pathways. Plasma levels of A, TC and B, TG in PCSK9^liver^*^(+/+)^*, PCSK9^liver^*^(+/-)^*, and PCSK9^liver^*^(-/-)^* mice fed a normal chow diet (n=6). Levels of C, PCSK9; D, TNFα; and E, IL-1β, H, TLR-2, and I, TLR-4 proteins (n=3). Phosphorylated levels of F, p65-NF-κB; G, AP-1; J, MyD88; K, PI3K; L, AKT; M, mTOR; N, p38-MAPK; O, ERK1/2; P, JNK proteins (n=3).


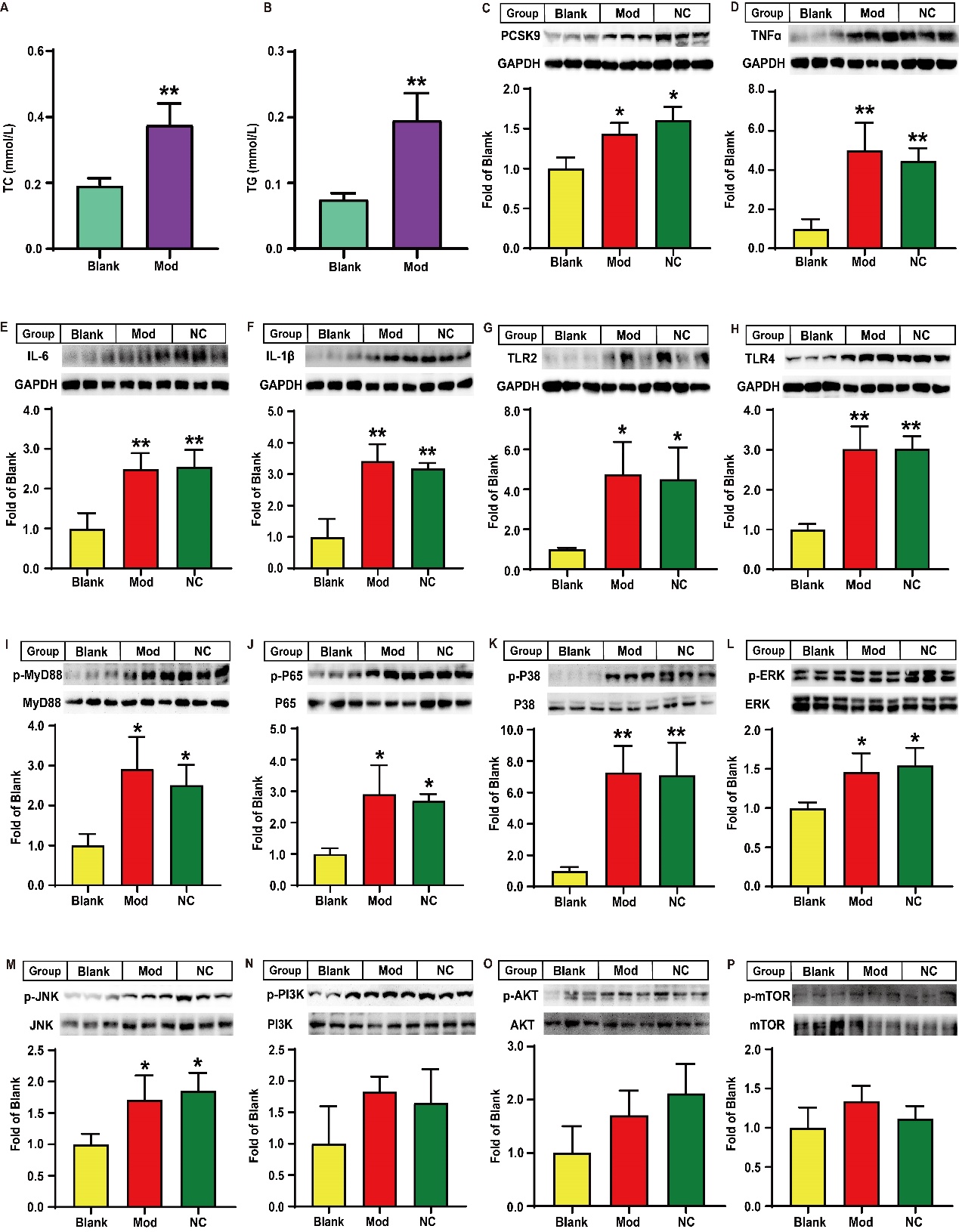


**Supplementary Figure 3.** Effects of lipid accumulation on the cellular levels of TC, TG, expression levels of PCSK9, proinflammatory factors, and key molecules involved in the TLR/MyD88, MAPK, and PI3K/AKT signaling pathways. Cellular levels of A, TC and B, TG (n=4). Levels of C, PCSK9; D, TNFα; E, IL-6; F, IL-1β; G, TLR-2; H, TLR-4 proteins (n=3). Phosphorylated levels of I, MyD88; J, p65-NF-κB; K, p38-MAPK; L, ERK1/2; M, JNK; N, PI3K; O, AKT; and P, mTOR proteins (n=3). Except for the blank control group, HepG2 cells were treated with 0.5 mM cis-9-octadecenoic acid and 0.25 mM palmitic acid for 24 h to establish a lipid-loaded cell model. Furthermore, the cells in the negative control (NC) group were transfected with the control siRNA by using Lipofectamine 3000. ^*^means *p* < 0.05 *vs* blank group; ^**^means *p* < 0.01 *vs* blank group.
